# Metal–Organic Framework Nanozymes: From Rational Design and Synthesis to Biomedical Applications

**DOI:** 10.1007/s40820-026-02168-x

**Published:** 2026-04-07

**Authors:** Shundong Cai, Mengdie Li, Songyi Wang, Xiaofei Wen, Gang Liu, Chengchao Chu

**Affiliations:** 1 Xiamen University Affiliated Xiamen Eye Center, Eye Institute of Xiamen University, School of Medicine, Xiamen University, Xiamen, 361102 People’s Republic of China; 2 Fujian Provincial Key Laboratory of Ophthalmology and Visual Science, Fujian Engineering and Research Center of Eye Regenerative Medicine, Xiamen, 361102 People’s Republic of China; 3 State Key Laboratory of Cellular Stress Biology, Innovation Center for Cell Biology, School of Life Sciences, Xiamen University, Xiamen, 361102 People’s Republic of China; 4 Department of Vascular & Tumor Interventional Radiology, The First Affiliated Hospital of Xiamen University, School of Medicine, Xiamen University, Xiamen, 361000 People’s Republic of China; 5 State Key Laboratory of Molecular Vaccinology and Molecular, Diagnostics & Center for Molecular Imaging and Translational Medicine, School of Public Health, Xiamen University, Xiamen, 361002 People’s Republic of China

**Keywords:** Nanozyme, Metal–organic framework, Tumor therapy, Anti-inflammation, Biosensing

## Abstract

Metal–organic framework (MOF) nanozymes have recently demonstrated considerable potential for biomedical applications. The catalytic activity of MOF nanozymes is primarily determined by the coordination of metal ions and organic ligands. Additionally, optimizing the morphology and pore size enhances the catalytic efficiency. However, a systematic framework bridging structural features to functional performance remains limited. To guide rational design, this review systematically analyzes how the selection of metal ions and organic ligands, as well as morphology, influences the catalytic activity of MOF nanozymes. The advantages and limitations of different synthesis methods are also summarized. Through concrete case studies, this review introduces major biomedical applications of MOF nanozymes, with a focus on their underlying catalytic mechanisms. Finally, key challenges confronting MOF nanozymes are critically analyzed, and future research directions are proposed.

## Highlights

- This review examines the influence of metal ions and organic ligands on the catalytic activity of metal–organic framework (MOF) nanozymes, thereby providing guidance for the rational design of MOF nanozymes.
- This review systematically summarizes recent advances in biomedical applications of MOF nanozymes.
- This review provides a detailed discussion on the current challenges and future research directions in the development of MOF nanozymes.

## Graphical abstract

**Figure:**
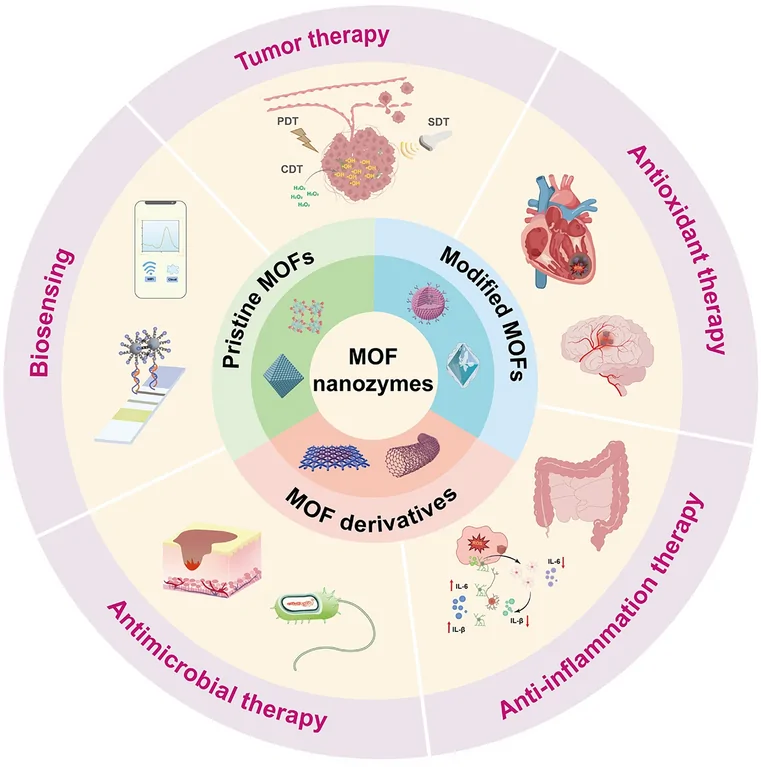

## Introduction

The human body harbors a diverse array of enzymes that catalyze biochemical reactions to maintain homeostasis [1]. However, natural enzymes exhibit inherent limitations, such as sensitivity to environmental conditions, high cost, and stringent storage requirements, which hinder their wide application [2]. In 2007, Yan group reported that magnetite nanoparticles (NPs) present enzyme-like activities similar to that of horseradish peroxidase [3]. Since then, a wide variety of nanomaterials with enzyme-like activities have been reported [4–6]. For instance, nanomaterials exhibiting catalase (CAT)-like activity can mediate the decomposition of hydrogen peroxide (H_2_O_2_) into H_2_O and O_2_ [7]. The artificial enzymes, which integrate the functions of nanomaterials and enzymes, are therefore named nanozymes [8]. Typically, nanozymes exhibit enhanced stability in harsh environments, facilitating the development of customizable catalytic platforms. Furthermore, their key advantages include scalable fabrication, cost-efficiency, and finely tunable enzyme-like activities [9].

**Fig. 1:**
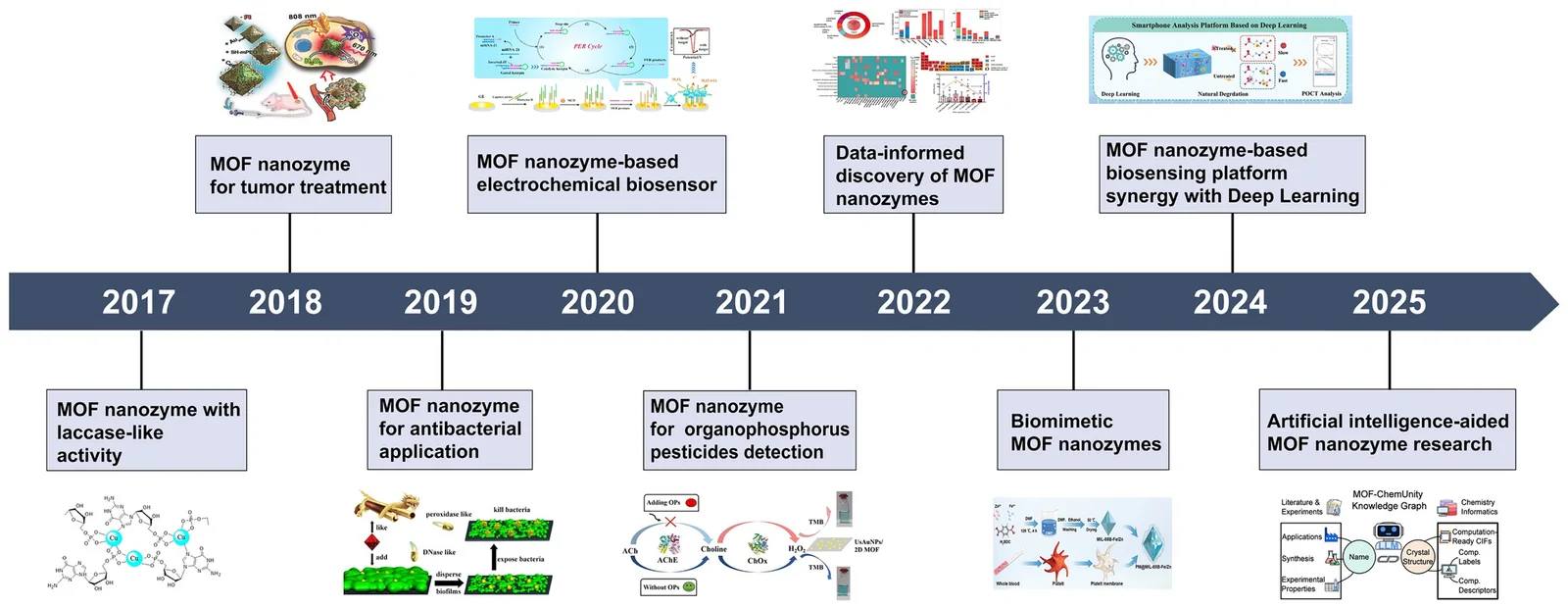
A brief timeline for the evolution of MOF nanozymes from 2017 to 2025. Figures are reproduced with permission [191–198]. Copyright 2016, American Chemical Society. Copyright 2018, Wiley–VCH. Copyright 2019, Elsevier. Copyright 2020, Elsevier. Copyright 2022, Elsevier. Copyright 2022, Springer Nature. Copyright 2023, Wiley–VCH. Copyright 2024, Wiley–VCH

Metal–organic frameworks (MOFs), formed through coordination-driven assembly of metal ions and organic ligands, represent a pivotal class of porous materials with versatile applications across multidisciplinary fields, such as catalysis [10], drug delivery [11], and biosensing [12]. Due to the ultra-high specific surface area and tunable porous architecture of MOFs, drug molecules can be encapsulated within the internal cavities, which not only shields vulnerable therapeutic agents from degradation but also enables controlled release kinetics [13]. Among the various nanomaterials with enzyme-like activities, MOF nanozymes have drawn considerable attention for biomedical applications. The first report of MOF nanozymes can be traced back to 2012. Feng et al. synthesized PCN-222(Fe) MOF and found that it exhibited peroxidase-like activity with excellent binding affinity and catalytic activity, which were even superior to hemin in aqueous media [14]. The number of publications on MOF nanozymes has increased rapidly since 2017, exhibiting an exponential growth trend and exceeding 250 publications per year by 2025 (Web of Science database), reflecting significant advancements in the field (Fig. 1). Compared with nanozymes of other materials (e.g., noble metals, metal oxides, carbon-based materials), MOF nanozymes exhibit the following advantages. Firstly, their structural tunability enabled by rational design of metal nodes and organic ligands allows precise mimicry of natural enzyme active sites [15]. In addition, introducing bimetallic nodes or heteroatoms can enable multi-enzyme synergistic catalysis. Secondly, the ultra-high specific surface area of MOFs provides abundant active sites, and the porous structure can restrict the movement of substrate molecules, therefore enhancing the contact efficiency between substrates and the active sites [16]. Moreover, drug molecules and imaging agents can be loaded in the pores of MOFs, thereby enabling the integration of diagnosis and therapy [17]. Furthermore, they offer synthetic controllability and stability. MOF nanozymes can be synthesized via hydrothermal reaction, microwave-assisted methods, or electrochemical methods under mild conditions. Commonly, they exhibit exceptional chemical and thermal stability due to crystalline coordination bonds, tolerating extreme pH and high temperatures [18]. Fourthly, their biosafety is designable. By employing biologically essential metal ions (e.g., Fe^3+^, Mn^2+^, Zn^2+^) and biodegradable ligands (e.g., endogenous nucleotides, amino acids, or simple organic acids), the prepared MOF nanozymes exhibit good biocompatibility and undergo gradual degradation into non-toxic byproducts under physiological conditions, thereby minimizing long-term toxicity concerns.

Benefiting from these advantageous properties, MOF nanozymes have been extensively explored. To date, numerous MOF nanozymes with diverse enzyme-like activities have been reported. A significant proportion of them function by modulating reactive oxygen species (ROS) levels, and these activities can be categorized into two classes: (1) ROS-scavenging activities (e.g., superoxide dismutase (SOD), CAT, glutathione peroxidase (GPx)), and (2) ROS-generating activities (e.g., oxidase (OXD), peroxidase (POD)) [19–21]. The former are commonly employed to manage diseases involving oxidative stress and inflammatory responses. MOF nanozymes mitigate pathological conditions by neutralizing excessive ROS at disease sites through the inherent enzyme-like activities. Conversely, the latter generate substantial ROS under the effect of endogenous stimuli (e.g., pH [22], H_2_O_2_ [23]) or exogenous stimuli factors (e.g., light [24], ultrasound [25]), enabling tumor cell or bacterial eradication. They are widely applied in tumor therapy and antimicrobial treatments. Additionally, some enzyme-like activities fall outside the two categories mentioned above, such as phosphatase. The phosphatase-like activity has extensive applications in the field of biosensing, such as for the detection of organophosphorus pesticides (OPs) [26]. Although numerous review articles on MOF nanozymes have been published in recent years, many focus on relatively specific application domains, such as therapy of inflammatory diseases [27], biosensing [28], and tumor therapy [29]. Moreover, several earlier comprehensive reviews predate the latest groundbreaking discoveries [30]. Consequently, there is a pressing need to provide a timely and updated review of this rapidly evolving field. More critically, a significant portion of the existing review articles pay insufficient attention to the rational structural design, which is crucial for guiding the future development of high-performance MOF nanozymes. For example, Wang et al. reviewed the applications of MOFs and their derivatives in the biomedical field [31]; Zhao et al. focused on the synthesis methods of MOF nanozymes and their applications in biomedicine [32]; Zhang et al. summarized the synthesis, activities, and broad biological applications of MOF nanozymes [33].

**Fig. 2:**
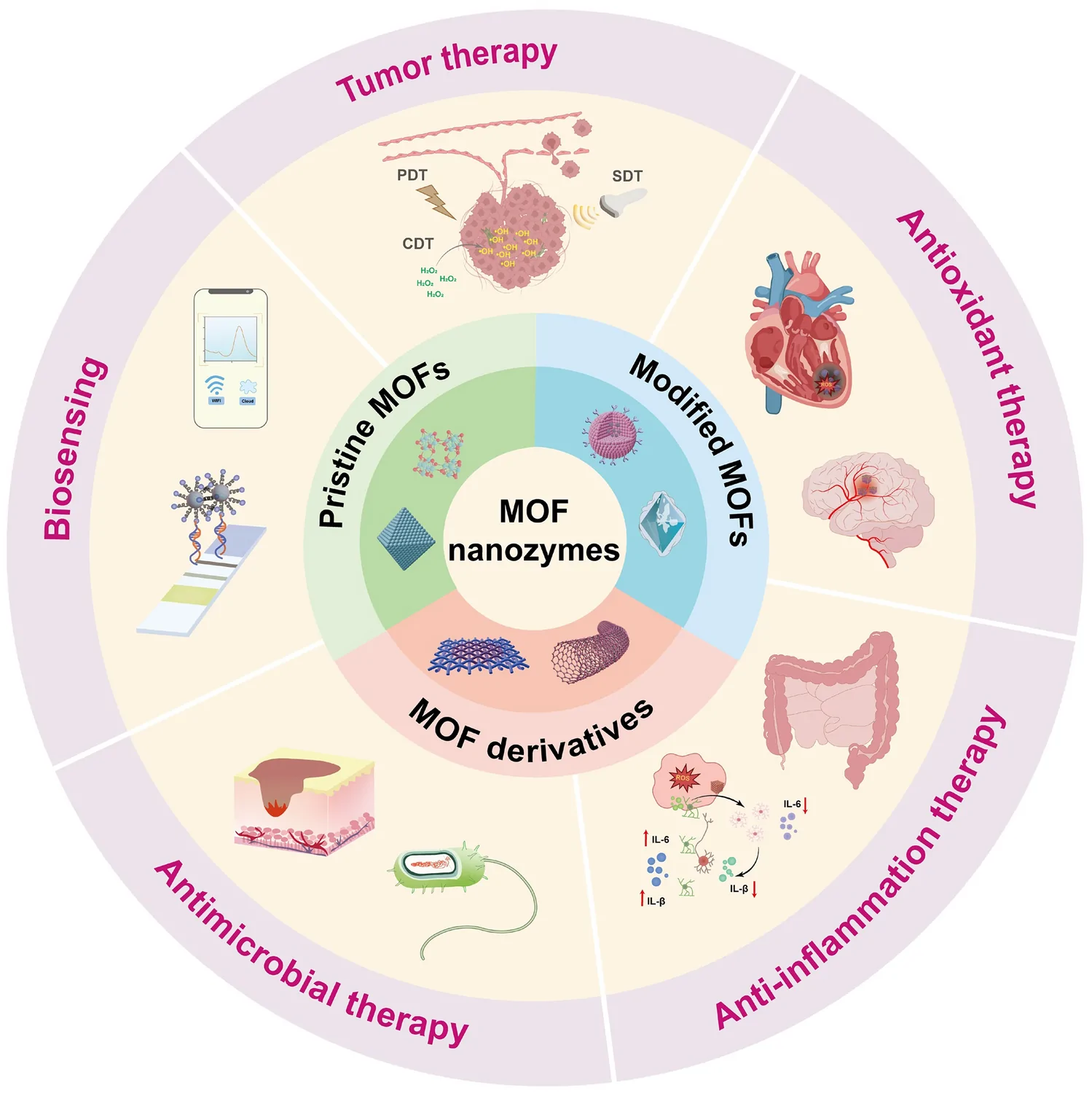
Schematic illustration of MOF nanozymes for biomedical applications

This review focuses on the structure–performance relationships of MOF nanozymes and systematically summarizes the rational design strategies, including the selection of metal ions and ligands, as well as the influence of morphology and porosity on the catalytic performance. The main synthesis methods of MOF nanozymes are summarized, and the advantages and limitations of each preparation approach are analyzed. Recent novel preparation methods for MOF nanozymes are also introduced. Through specific case studies, the biomedical applications of MOF nanozymes are reviewed, encompassing tumor therapy, oxidative stress- and inflammation-related disease therapy, antibacterial therapy, and biosensing (Fig. 2). The fundamental mechanisms by which MOF nanozymes exert their effects in these applications are elucidated. Finally, this review discusses the key challenges confronting the field and proposes promising future research directions.

## Rational Designs of MOF Nanozymes

Metal ions and organic ligands are the main components of MOF nanozymes, whose properties fundamentally determine the catalytic activity of the synthesized MOF nanozymes [34, 35]. From the perspective of rational design, this section provides a comprehensive summary of how the properties of metal ions and organic ligands influence the catalytic activity of MOF nanozymes, offering guidance for rational design. Furthermore, artificial intelligence (AI)-assisted design strategies are also introduced.

### Metal Ions

Serving as the active centers within MOFs, the metal ions play a critical role in determining the catalytic activity through their intrinsic properties, such as redox activity, Lewis acidity, metal ion size, and coordination environment. Redox-active metals (e.g., Fe^2+^/Fe^3+^) participate in electron transfer via valence state changes. Therefore, MOF nanozymes synthesized with these metal ions typically exhibit POD-like activity. For example, MIL-100(Fe) MOF can catalyze H_2_O_2_ decomposition to generate ·OH radicals in Fenton-like reactions, enabling tumor chemodynamic therapy [36]. Copper ions (Cu^+^/Cu^2+^) possess dual valence states, enabling them to mimic the activities of OXD, SOD, and POD. The electron-deficient nature of Cu^+^ facilitates its binding with O_2_ to form superoxide anions, while Cu^2+^ achieves SOD activity through the disproportionation reaction. Zhang et al. prepared Cu-based MOF (Cu-MOF) nanozyme with highly efficient ROS scavenging that alleviates oxidative stress for acute pancreatitis treatment [37]. It was found that Cu-MOF exhibited excellent SOD- and CAT-like activities, which were much higher than those of Ni and Co MOFs. This result demonstrates that the type of metal ion plays a decisive role in determining the catalytic activity of MOF nanozymes. MOF nanozymes prepared with Mn^2+^ ions (e.g., PCN-222(Mn)) typically exhibit CAT-like activity. On one hand, Mn^2+^ ions can decompose H_2_O_2_; on the other hand, the active centers of PCN-222(Mn) MOF feature an Mn-porphyrin structure, which is highly similar to the heme structure of natural CAT. Due to the magnetic properties of manganese ions, Mn-based MOF nanozymes can be traced via magnetic resonance imaging (MRI), thereby monitoring their distribution and metabolism in vivo. Additionally, manganese ions possess immunomodulatory functions. For instance, they can activate the cGAS-STING pathway to enhance the efficacy of tumor immunotherapy [38].

In addition, Lewis acidic metals (e.g., Ce^4+^, Zr^4+^, Cu^2+^) stabilize transition states by coordinating with electron-rich bonds (e.g., P = O, C = O), facilitating nucleophilic attacks on substrates. Therefore, MOF nanozymes with these Lewis acidic nodes typically exhibit hydrolase activity, and they are often applied in the construction of biosensing platforms. For example, Zhou et al. developed a Zr-MOF nanozyme platform for degradation and real-time monitoring of organophosphorus pesticides (OPs) in water [39]. The MOF nanozyme was synthesized via the solvothermal method, and then, the aptamers against OPs were modified on the surface of MOF by covalent bonds. Experimental results revealed that it was the aptamers that played the role of bionic arms and realized active grasping of the specific target objects and hindrance of nontarget objects. The enhanced phosphatase-like activity of MOF nanozyme benefited from two factors: first, the high affinity of the aptamers, and second, the cluster formation between aptamers and the MOF core, which significantly improved the affinity between the biomimetic nanozyme and OPs. The degradation process can be recorded in real time depending on the color change of the reaction solution originating from the degradation products.

Some metal ions (e.g., Ag^+^, Cu^2+^, Zn^2+^) show great sterilization effects. The primary working mechanisms involve disrupting the phospholipid bilayer structure of cell membrane and inhibiting bacterial enzyme activity, thereby affecting normal metabolic functions [40]. By synergizing with the ROS-generating capacity of POD-like activity, the antibacterial performance of MOF nanozymes can be significantly enhanced. A notable advantage of this strategy is that bacteria are less likely to develop antibiotic resistance. MOF nanozymes constructed by these metal ions can be incorporated in wound dressings to combat infections and promote wound healing [41]. Zhong et al. prepared AgAu-modified hybrid MOF nanozyme (AgAu@QMIL-53) with multiple enzyme-like activities to eradicate bacteria [42]. Benefiting from the OXD- and POD-like activities, the nanozyme can convert O_2_ and H_2_O_2_ into ·O_2_^−^ and ·OH, which can kill bacteria by oxidative stress. In vitro antibacterial experiments confirmed that after modification of the AgAu alloys, the hybrid nanozyme exhibited excellent antibacterial effects against *P. aeruginosa* (99.05%) and *MRSA* (99.17%) at a low dosage.

Introducing two metal elements into the MOF structure to create bimetallic MOF nanozymes can enhance the catalytic activity [43], and this can be attributed to the following mechanisms. First, the electronic synergy effect, where the potential difference and electronic coupling between bimetallic ions are key to enhancing catalytic activity. When two metals (e.g., Co^2+^/Ni^2+^, Fe^3+^/Cu^2+^) coexist in the MOF nanozyme, electron transfer channels are formed to promote charge transfer and lower the reaction energy barrier. Second, the multi-active-site synergistic effect. The spatial coordination environment and electron density of bimetallic ions can synergistically optimize active sites, enhancing substrate adsorption and activation efficiency. In addition, bimetallic MOFs can mimic the multi-active center structure of natural enzymes, enabling cascade catalysis or multi-substrate synergistic processing. Third, dynamic valence state transitions maintain an efficient redox cycle. The reversible valence state switching of bimetallic ions sustains dynamic redox balance and avoids active site inactivation. For instance, Ma et al. developed a single-site copper–bipyridine-based cerium MOF (Cu/Ce-MOF@M) for tumor therapy [44]. They demonstrated that this bimetallic nanozyme exhibited 32.1-fold higher catalytic activity compared to traditional MOFs loaded with Cu NPs, attributed to synergistic electronic interactions between Cu and Ce species.

### Organic Ligands

As the other core component of MOF nanozymes, organic ligands are not merely structural scaffolds but also crucial regulators of catalytic activity, biocompatibility, and stability. The chemical properties of organic ligands significantly influence the enzyme-like activities of MOF nanozymes. They directly modulate the electronic structure of metal active centers, substrate adsorption capacity, and catalytic pathway selection [45]. Therefore, the selection of organic ligands warrants careful consideration in the design of MOF nanozymes.

Phthalic acid and its derivatives are the most classic organic ligand families for MOF construction. By introducing different substituents into the benzene ring, the electronic structure of MOFs can be systematically tuned. Electron-withdrawing groups render the metal center more electron-deficient, which enhances its ability to accept electrons from substrates, significantly boosting OXD- and POD-like activities [46]. Furthermore, modulating electronic effects can adjust substrate adsorption energy, influence reaction barriers, and alter reaction rates. Wu et al. employed a ligand engineering strategy to systematically investigate the GPX-like activity of MOF nanozymes prepared by terephthalate with different substituted ligands [47]. They found that the MOF nanozyme prepared with ligands substituted with an amino group (-NH_2_) exhibited the best GPx-like activity, which can be applied as a ROS scavenger to inhibit inflammatory responses.

Some ligands themselves possess catalytic activity, and these ligands enable highly biomimetic catalysis. The most representative class is metal porphyrin ligands, such as tetrakis(4-carboxyphenyl)porphyrin (TCPP) and its metal derivatives (e.g., iron tetrakis(4-carboxyphenyl)porphyrin, Fe-TCPP) [48]. Porphyrin-based multidentate ligands stabilize metal clusters and extend π-conjugation systems. In TCPP-Fe MOFs, porphyrin ligands promote electron delocalization, boosting oxidative enzyme-like activity. Synergistic effects between metal ions and ligands via electronic coupling and spatial interactions further optimize catalytic pathways. The MOF framework immobilizes porphyrin molecules, preventing their aggregation and inactivation. Meanwhile, the large mesopores of the MOF provide channels for the free diffusion of substrates, resulting in a catalytic efficiency that even surpasses that of free porphyrin molecules.

The geometric structure of organic ligands also has a significant impact on the catalytic activity of nanozymes. This influence is primarily manifested in the regulation of MOF pore size, channel shape, and pore surface properties by the ligands’ length, rigidity, and functional group position [49]. A large pore size facilitates the diffusion of macromolecular substrates, while the chemical environment within the pores critically influences the stability of reaction intermediates. The structure of the organic ligand is crucial for the stability of MOF nanozymes. To enable application in harsh environments, rigid ligands that can form strong coordination bonds with metals should be selected, such as trimesic acid with carboxyl groups, which can form strong coordination bonds with metal ions to ensure structural stability. In contrast, if the MOF nanozyme needs to degrade in a specific environment to release loaded drug molecules, ligands with weaker coordination can be used. One representative MOF is ZIF-8 formed by 2-methylimidazole and zinc ions, which can gradually degrade in acidic environments [50]. Additionally, ligands featuring unsaturated bonds (e.g., C = C, C≡C, or C = N) are ideal choices for subsequent functionalizations. Their core advantages lie in the high reactivity of unsaturated bonds and the ability to precisely control the structure and properties of MOFs. By introducing unsaturated structures, these ligands provide active sites for subsequent covalent modifications. Meanwhile, they significantly optimize the catalytic, adsorption, or optoelectronic properties of MOFs by altering their electronic structure, pore environment, and metal–ligand interactions.

Stimuli-responsive ligands are suitable for constructing smart drug delivery systems. For instance, MOFs derived from carboxyl- or imidazole-containing ligands undergo gradual degradation in acidic environments [51]. Therefore, the loaded drugs or biomolecules can be released in a sustained or controlled manner for disease therapy. In addition, MOFs constructed with disulfide bond-containing ligands can be cleaved by endogenous glutathione (GSH), thereby achieving GSH-responsive drug release [52]. For instance, Fan et al. prepared MOF nanozymes using TCPP-Fe as the ligand for synergistic tumor photodynamic therapy and immunotherapy [53]. TCPP ligands and iron ions are continuously released from the MOF structure under the high concentration of GSH at the tumor site. TCPP-mediated PDT and iron-mediated Fenton reactions jointly promoted ferroptosis of tumor cells.

The selection of organic ligands may also affect the morphology and size of the synthesized MOF nanozymes, thereby further determining their catalytic activity. This is realized by adjusting the exposure of active sites and substrate diffusion efficiency. For example, two-dimensional MOFs expose more active sites compared to traditional three-dimensional MOF architectures, thereby shortening substrate diffusion pathways. Additionally, MOF nanozymes with nanoflower structures have been developed in recent years [54, 55], demonstrating enhanced enzymatic activity. These structural optimizations can be achieved through solvent thermal methods or microwave-assisted synthesis to tailor morphologies (e.g., nanosheets, porous frameworks), or by introducing surface-active agents to optimize pore size distribution. In future development, more attention should be paid to application-oriented design principles for MOF nanozymes. For instance, engineering ligands that not only modulate the electronic structure of metal nodes but also possess intrinsic catalytic activity and respond to specific stimuli (e.g., light, pH). This approach aims to achieve intelligent, precise catalytic applications tailored to specific needs.

### Artificial Intelligence-Assisted Design of MOF Nanozymes

Artificial intelligence (AI) technology, particularly machine learning (ML), is fundamentally transforming the research paradigm of MOF nanozymes and shifting the traditional trial-and-error experimental approach into a data-driven, predictable, and high-throughput rational design process, significantly accelerating the discovery and application of high-performance MOF nanozymes [56]. ML models are well-known for their high predictive accuracy, computational efficiency, and low cost, and this has encouraged researchers to advance this field [57]. ML excels in analyzing and identifying the structure–property relationships within complex systems, and it has emerged as an indispensable tool in the pursuit of novel material development. In this chapter, we briefly introduce the basic principles and application cases of AI-assisted MOF nanozyme design. For more in-depth summaries, please refer to recently published  relevant review articles [58–60].

In MOF nanozyme research, ML models leverage AI and vast datasets to develop increasingly accurate predictions. These predictions can be experimentally validated, with the resulting data fed back into the models to further refine their accuracy. AI-assisted MOF discovery leverages predictive models trained on large datasets to forecast synthesis outcomes, predict material properties, and even generate entirely new MOF structures with user-defined functionalities. By identifying patterns in massive datasets that would be impractical for humans to detect within a similar timeframe, ML allows scientists to focus their efforts on laboratory testing and validation of model-generated insights. The structure and application of MOFs can be finely adjusted by altering the metal nodes, organic linkers, and the reaction conditions. This high degree of tunability poses a challenge for researchers seeking the most suitable MOF for specific applications under optimal synthesis conditions. ML offers a promising solution to streamline this process by accelerating the screening of optimal MOFs based on structure and functionality. Recently, Pang group applied ML to conduct a series of studies in the design and synthesis of MOFs [61–63]. The ML-based predictive model successfully identified core parameters governing phase transition processes.

ML models can be trained using the SynMOF database, which was generated through automatic data extraction from the CoRE MOF database and literature, recording six parameters, including metal source, linker, solvent, additive, synthesis time, and temperature, for the recorded MOFs [64]. A web tool was developed based on this model, which, when given the CIF file of a MOF structure, can predict synthesis conditions including temperature, reaction time, solvent, and additive. In addition, ML can be applied in inverse design of MOFs with specific functionalities. For instance, Li et al. reported the integration of ML model with a genetic algorithm to enable inverse design of MOFs for gas separation [65]. The ML model, trained on structural features including topology, metal/organic secondary building units, and functional groups, was embedded within the genetic algorithm framework to efficiently navigate the high-dimensional chemical space. Feature importance analysis highlighted topology as the dominant factor governing selectivity, followed by linker identity and functionality. Structural realization of the genotypes led to the identification of an optimal MOF comprising an fsc net, zinc paddlewheel inorganic units, and 1-ethylnaphthalene and 2,7-dimethylphenazine as organic linkers. This study highlighted a paradigm shift from trial-and-error screening toward goal-directed MOF design.

Despite the significant application potential demonstrated by AI-assisted MOF design, several challenges remain to be addressed. First, most databases suffer from inconsistency, noise, and limited experimental validation, with issues such as incomplete structures, poor labeling, and solvent effects hampering their usability. Second, on the modeling side, many ML frameworks remain black boxes, offering limited interpretability and making it difficult to establish robust structure–property relationships that can guide synthesis. Future integration of multimodal data analytics with automated experimental platforms may realize full-chain intelligent workflows spanning “design–synthesis–application” of MOF nanozymes.

## Classification and Synthesis of MOF Nanozymes

### Classification of MOF Nanozymes

**Fig. 3:**
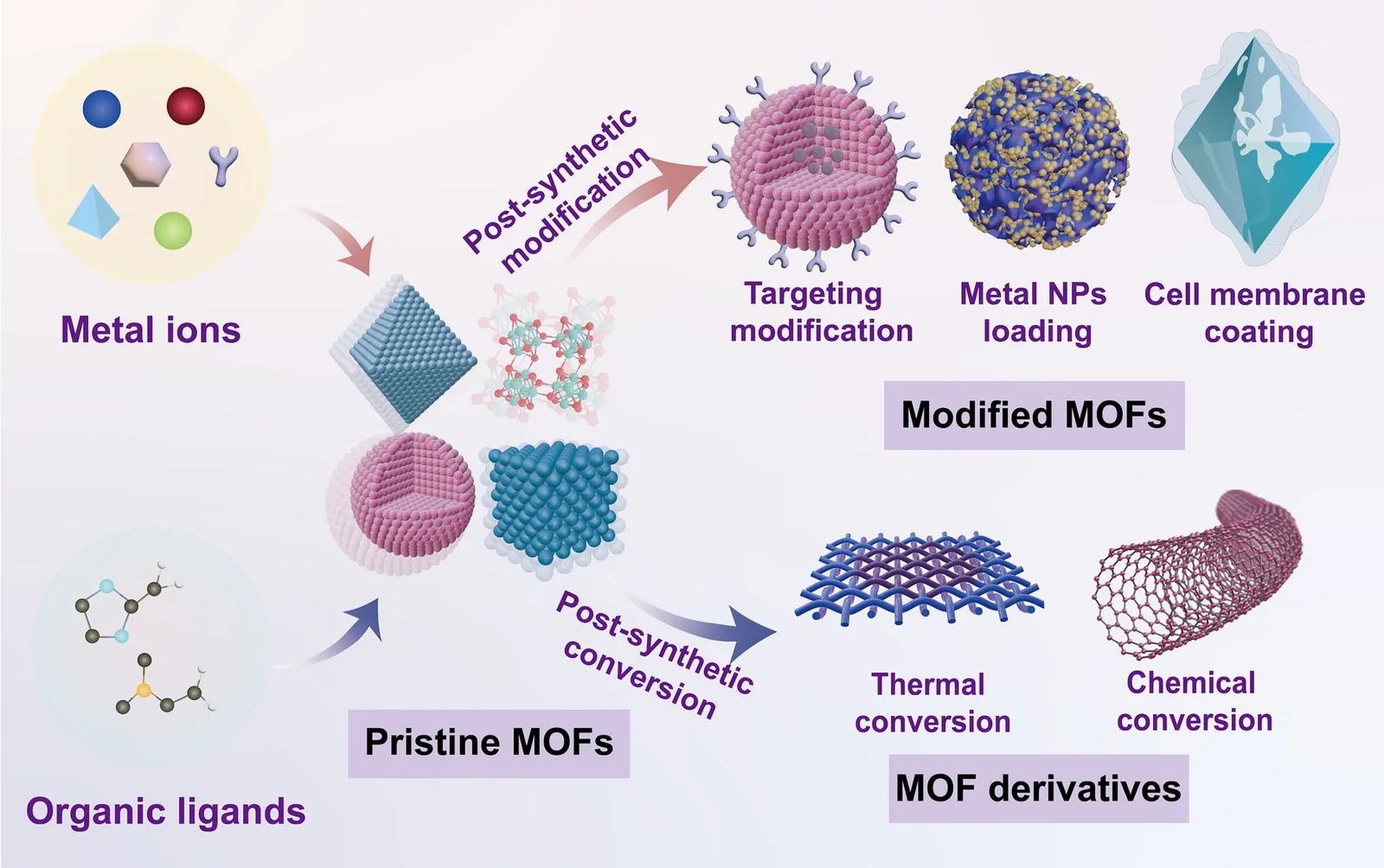
Classification and representative diagrams of MOF nanozymes

Based on the composition and structure, MOF nanozymes can be categorized into three main categories: pristine MOFs, modified MOFs, and MOF derivatives (Fig. 3). Pristine MOFs refer to crystalline porous materials formed by self-assembly of metal ions and organic ligands without chemical modification or structural alteration. They retain the original framework structure and pore characteristics of MOFs, and their catalytic activity relies on the metal centers or ligands inherent in the porous structure. The pore size of MOF nanozymes is primarily determined by the coordination environment of the metal nodes and the molecular dimensions of the organic ligands [66]. These pores can be categorized into three types: micropores (< 2 nm), mesopores (2–50 nm), and macropores (> 50 nm). Microporous MOFs typically have pore sizes ranging from 0.5 to 2 nm. For example, zeolitic imidazolate framework-8 (ZIF-8) has a pore size of approximately 1.16 nm. Similarly, UiO-66 MOF exhibits pore sizes of about 1.4–1.6 nm. Mesoporous MOFs, on the other hand, have larger pore sizes ranging from 2 to 50 nm [67]. A notable example is PCN-333, which has pore dimensions of approximately 2.0–6.2 nm. Owing to their large surface areas and accessible metal sites, some pristine MOF NPs inherently exhibit enzyme-mimicking activities [68–70]. Representative pristine MOF nanozymes include HKUST-1 [71] and UiO-66(Zr) [72], among others. Modified MOFs refer to MOFs with functional components (e.g., metal NPs, ligands, biomolecules) introduced on their surfaces or within pores to enhance existing properties or impart new functionalities while preserving the primary structural framework. This is achieved via coordination bonds, covalent bonds, or physical adsorption. Metal NPs (e.g., Au, Pt) can be loaded onto MOF surfaces via in situ reduction or physical adsorption. This strategy can enhance electron transfer kinetics within MOFs, thereby boosting the enzyme-mimicking activities [73, 74]. The loading amount of metal NPs primarily depends on the pore size of the MOFs and the size of the metal NPs, which typically ranges from 1% to 10% by weight. Furthermore, functional modifications of MOFs can be tailored to meet specific application requirements. For instance, conjugating targeting moieties onto MOFs can enhance their targeted accumulation at disease sites [75]. In addition, encapsulating MOF nanozymes within hydrogels can prolong the drug retention time [76]. MOF derivatives are obtained through the pyrolysis or chemical transformation of pristine MOFs under controlled conditions, resulting in materials such as metal oxides/sulfides, metal–carbon hybrids, and porous carbon materials. Some of these materials exhibit enhanced enzyme-like activity or even multi-enzyme functionality [77, 78]. The average specific surface area of MOF derivatives, which commonly ranges from 100 to 1500 m^2^ g^−1^, serves as a key indicator of their catalytic performance. This parameter is predominantly influenced by the derivatization methods and the type of metal ions.

### Synthesis of MOF Nanozymes

Currently, a variety of synthesis methods for MOF nanozymes have been reported. They include conventional methods such as hydrothermal and solvothermal methods, as well as a variety of novel synthesis techniques. These methods promote the coordination reaction between metal ions and organic ligands through different mechanisms. Due to differences in their intrinsic mechanisms and the equipment employed, each method possesses its own advantages and limitations. In this section, we provide a comprehensive summary of the currently reported preparation methods for MOF nanozymes. This includes their fundamental principles, cost considerations, potential for scalable production, and typical product morphologies, along with analysis of the advantages and limitations of each approach. These contents are summarized in Table 1. Additionally, we introduce the strategies for post-synthetic modification of MOF nanozymes.

**Table 1:** Summary and comparison of typical synthesis methods of MOF nanozymes

	Hydrothermal method	Solvothermal method	Ultrasonic-assisted method	Microwave-assisted method	Electrochemical synthesis	Microfluidic synthesis	Low-temperature plasma synthesis	Solvent-free mechanochemical synthesis
Process parameters	100–250 ℃, 3–48 h	100–250 ℃, 3–48 h	RT, several minutes to one hour	RT, several minutes to one hour	RT, minutes to hours, voltage/current: 0.5–10 V, 0.1–20 mA cm^−2^	Seconds to minutes	Seconds to minutes	Within minutes to tens of minutes
Cost	Low	Medium	Low	Low	Medium	High	Low	Low
Scalability potential	Low	Low	Medium	Medium	Low	High	Medium	High
Typical structural outcomes	Polyhedra NPs	Diverse morphologies	Tend to form special morphologies, such as two-dimensional nanosheets	Uniform crystal size and high crystallinity	Thin films and complex structures	Diverse morphologies that can be tuned by adjusting parameters	Diverse morphologies that can be tuned by adjusting parameters	relatively high purity, while the crystallinity may be lower than that of traditional methods
Advantages	Low cost, high safety, environmental friendliness	Diverse morphologies can be adjusted	Ultrafast reaction rate, mild reaction conditions	Ultrafast reaction rate, uniform crystal size, high crystallinity	Mild reaction conditions, fast reaction rate, high product purity	Ultrafast reaction rate, size distribution is narrow, and the morphology is controllable	Ultrafast reaction rate, diverse morphologies can be adjusted	Environmentally friendly, high efficiency, high atom economy
Limitations	Low batch production, long reaction time	High cost, toxicity, flammability	May introduce defects, challenging to scale-up production	Requires a specialized microwave reactor with precise control over power and time	Difficulty in maintaining uniformity during large-scale production	Technical complexity, high cost, potential risk of channel clogging	Equipment complexity and cost, process optimization difficulty, scalability challenges	Poor mass and heat transfer control, equipment dependency, scaling-up challenges

#### Hydrothermal and Solvothermal Method

The majority of MOF nanozymes are synthesized in solution through coordination reactions between metal ions and organic ligands. Depending on the solvent used, the methods are categorized as the hydrothermal method or the solvothermal method [79]. The former refers to synthesizing MOF nanozymes in an aqueous solution. Typically, the coordination reaction occurs with magnetic stirring and elevated reaction temperatures. This method is primarily used for preparing MOF nanozymes that are not sensitive to water. The solvothermal method​generally employs non-aqueous organic solvents such as dimethylformamide (DMF), methanol, ethanol, or mixtures of these organic solvents [80]. Among them, DMF is the most widely used. Briefly, metal ions and organic ligands are first mixed uniformly using ultrasonication. Then, the mixture is transferred into a Teflon liner, which is sealed within a stainless-steel autoclave and reacted at high temperature (100–300 °C) for a set duration (12–36 h). The resulting products are collected by centrifugation and washed with solvent to obtain pure MOF nanozymes, which typically have sizes around 50–200 nm. The morphology of the nanozymes can be adjusted by the types and ratios of metal ions and organic ligands [81, 82]. MOF nanozymes prepared by the hydrothermal method typically exhibit uniform morphology, often forming standard polyhedra such as octahedra or dodecahedra. The homogeneous crystal growth environment facilitates the synthesis of MOF nanozymes with uniform size distribution. Using water as the solvent offers several advantages, such as low cost, high safety, and environmental friendliness. However, the uniformity of heat and mass transfer may decrease during scale-up, which restricts its potential for large-scale production of MOF nanozymes. In contrast, MOF nanozymes synthesized via the solvothermal method display highly diverse morphologies. By adjusting the polarity and coordination ability of organic solvents, precise morphological controls can be achieved [83]. Nonetheless, organic solvents generally entail higher costs, and their toxicity and flammability require careful handling in large-scale production. Additionally, the recycling and treatment of these solvents add both economic and environmental burdens.

#### Ultrasonic- and Microwave-Assisted Method

Traditional hydrothermal and solvothermal methods for synthesizing MOFs typically require high reaction temperatures and long reaction times. To overcome these limitations, ultrasound- and microwave-assisted synthesis methods have been widely adopted in recent years. The ultrasound-assisted method utilizes the cavitation effect generated by ultrasonic waves, which creates localized extreme temperatures and pressures in the mixture of metal ions and organic ligands, vigorously agitating the reactants and accelerating nucleation [84]. This process can usually be conducted at room temperature (RT), significantly reducing the reaction time to several minutes. The ultrasound-assisted method facilitates the preparation of two-dimensional (2D) MOF nanozymes. Furthermore, under high-power ultrasound, 2D MOF materials can be fragmented into zero-dimensional MOF nanodots, further enhancing their catalytic activity. Nevertheless, the ultrasound-assisted method may introduce structural defects into the MOFs due to the instantaneous high-energy input. Moreover, scaling up production poses challenges for reactor design [85, 86]. Microwave-assisted synthesis utilizes the volumetric heating effect of microwaves on polar molecules, achieving rapid and uniform temperature increase at the molecular level, which promotes synchronous nucleation and crystal growth [87]. Similar to the ultrasound-assisted method, it offers an extremely fast reaction rate, often completing within several minutes to one hour. The resulting products exhibit uniform crystal size, high crystallinity, and controllable defects. By precisely adjusting the microwave power and irradiation time, the morphology and defect concentration of the obtained MOF nanozymes can be effectively regulated. However, ensuring field uniformity within large reaction chambers is challenging in large-scale production. For applications requiring specific morphologies of MOF nanozymes, the ultrasonic-assisted method is preferable. In contrast, the microwave-assisted method is more suitable to prepare products with high crystallinity and uniformity [88, 89]. We propose that combining these two methods to leverage their respective advantages presents a promising approach for preparing MOF nanozymes.

#### Electrochemical Method

Distinct from traditional hydrothermal and solvothermal methods, electrochemical synthesis offers an innovative strategy for preparing MOF nanozymes. It utilizes an electric field as the driving force to achieve controllable coordination between metal ions and organic ligands under ambient temperature and pressure, making it particularly suitable for synthesizing high-quality thin films and MOFs with complex structures [90]. The reactions typically proceed at RT and atmospheric pressure, often completing within minutes to hours. Compared with other synthesis methods, electrochemical synthesis exhibits several key advantages, including mild reaction conditions, fast reaction rate, as well as high product purity with controllable crystal size and thickness. In addition, this method has relatively low energy consumption. However, scaling up production of MOF nanozymes by electrochemical synthesis presents some challenges. First, it strongly relies on conductive substrates for the reactions, therefore narrowing the application scope of this synthesis method. Second, it is difficult to maintain uniformity of MOF nanozymes during large-scale production. Third, it may cause side reactions such as hydrogen evolution during the synthesis of MOF nanozymes.

#### Microfluidic Method

Microfluidic technology has also been applied for MOF nanozyme synthesis recently. It can precisely manipulate fluids in micro-scale channels, shifting synthesis from traditional batch reactions to continuous flow production. This approach enables extremely efficient mass and heat transfer, allowing reactions to complete within seconds to minutes [91]. Commonly, the prepared MOFs have highly uniform crystal size, controllable morphology, and narrow size distribution. Precise control over parameters such as temperature, flow rate, and residence time can be achieved in real-time, thereby controlling the morphologies of the products. Despite the advantages, microfluidic synthesis of MOF nanozymes also faces some challenges, mainly including high technical complexity and cost due to specialized device design, a persistent risk of channel clogging from particle aggregation, and engineering hurdles in scaling up production.

#### Low-Temperature Plasma Method

Low-temperature plasma technology is an efficient and energy-saving strategy for the synthesis of MOF nanozymes. Its core lies in the unique energy transfer mechanism. Under ambient temperature and pressure, a high-voltage electric field ionizes the introduced gas (e.g., argon, nitrogen, or their mixtures), generating a plasma containing high-energy electrons, ions, excited-state molecules, and free radicals. These highly reactive species effectively impact reactants in the solution or solid precursors, supplying the necessary energy for the coordination between metal ions and organic ligands [92]. Commonly, the reactions finish within one minute, thereby bypassing the prolonged high-temperature and high-pressure environments required by traditional methods. Consequently, the energy consumption of this synthesis method is significantly lower than other methods. Utilizing this technique enables the preparation of MOF nanozymes with special structures (e.g., amorphous states, nanoflowers). Key structural characteristics of the material, such as oxygen vacancy concentration and metal valence state distribution, can be controlled by precisely adjusting discharge parameters, such as the power, gas ratio, and time. Simultaneously, this method also presents certain limitations. First, equipment complexity and cost should be taken into consideration. The initial investment for specialized plasma reactors is higher than for traditional equipment. Second, the difficulty of process optimization. The discharge parameters of low-temperature plasma greatly influence the product outcome. Therefore, process development is relatively complex and requires optimization experiments. Third, maintaining plasma uniformity within large reaction chambers poses technical challenges for scaled-up production, potentially affecting batch-to-batch consistency.

#### Solvent-Free Mechanochemical Method

Solvent-free mechanochemical synthesis is a green approach for MOF nanozyme preparation. It utilizes mechanical energy (e.g., shear forces and friction generated by grinding or extrusion) to disrupt the crystal structure of reactants, promoting molecular diffusion and chemical reactions at solid–solid interfaces [93]. This process typically occurs at RT or moderately low temperatures, avoiding the need for high temperatures or pressures. The reaction rate is fast, often completing within minutes to tens of minutes. Since large amounts of solvent are not required, fewer impurities are introduced, leading to products with higher purity compared to those made by traditional methods. However, the crystallinity of the resulting products may be lower than that achieved by conventional methods, though it can sometimes produce unique crystal forms that are difficult to obtain otherwise. Solvent-free mechanochemical synthesis exhibits several key advantages. Primarily, it is green and environmentally friendly, as it minimizes or eliminates solvent use, reducing pollution and waste streams at the source. In addition, it is highly efficient and energy-saving, operating under mild conditions with short reaction times and low energy consumption. The method also achieves high atom economy, including high reaction conversion rates and efficient utilization of raw materials [94]. For large-scale production, this method has several limitations. First, mass transfer among solid reactants is less efficient than in solution, and heat released from reactions may cause local overheating, presenting challenges in heat and mass transfer control. Second, the relationship between mechanical parameters (e.g., force and frequency) and the final product structure is complex, which requires careful process optimization.

#### Post-Synthetic Modification

Post-synthetic modification of pre-synthesized MOF materials can enhance their catalytic performance or endow the nanozymes with specific functionalities. MOFs can accommodate dense metal NPs through the rigid crystalline pores, with catalytic centers precisely incorporated onto organic linkers or inorganic nodes to engineer uniform active sites [95]. In recent years, diverse metal NPs-loaded MOF nanozymes have been synthesized. The incorporation of metal NPs can enhance electron transfer within the MOF framework, thereby boosting enzyme-like activities. In addition, it can endow intrinsically non-enzymatic MOF structures with the catalytic activity derived from the metal NPs [96]. Generally, they can be synthesized via two strategies. First is in situ reduction method. This widely employed approach involves mixing metal NPs precursors (e.g., chloroplatinic acid, chloroauric acid) with pre-synthesized MOF NPs, alongside incorporation of reducing agents. Under magnetic stirring, the metal ions are reduced, and metal NPs grow in situ on the MOFs [97]. Second is one-pot solvothermal method. Pre-synthesized metal NPs are added into the solution containing metal ions and organic ligands, and the metal NPs-loaded MOF nanozymes can be synthesized via solvothermal method [98]. Surface modification of targeting agents can endow MOF nanozymes with targeting capability toward disease sites [99]. It can be achieved through two primary strategies. First is covalent conjugation. It involves forming chemical bonds between targeting molecules and the MOF surface, ensuring stable attachment and resistance to detachment during in vivo applications. For instance, introducing amino groups onto the MOF surface allows activation with EDC/NHS to couple with targeting agents (e.g., antibodies, peptides). However, challenges include potential alterations in nanozyme activity or production loss during purification, which should be taken into consideration. ​Second is physical adsorption. It leverages electrostatic interactions or confinement effects to attach targeting groups to MOF nanozymes, offering simplicity but risking partial detachment due to weak binding strength. In addition, coating the NPs with cell membrane or vesicles (CMVs) derived from specific cell sources can realize targeted accumulation of MOF nanozymes at disease sites [100, 101]. For example, encapsulating MOF nanozymes with cell membrane derived from 4T1 breast cancer cells can enhance their targeting to the tumor site, significantly improving antitumor efficacy. Zheng et al. found that after coating the nanomedicine with 4T1 cell-derived cell membranes and co-incubating them with 4T1 cells, the fluorescence intensity of intracellularly taken-up DOX was twofold stronger than that of uncoated nanomedicine, which demonstrates the excellent targeting ability of homologous cell membrane coating [102]. In addition, encapsulating MOF nanozymes into hydrogels can prolong their retention time at the disease sites, and this strategy is often applied in the preparation of wound dressings [103].

## Enzyme-Like Activities of MOF Nanozymes

To date, MOF nanozymes with diverse enzyme-like activities have been reported. According to the relationship with ROS, generally MOF nanozymes can be divided into two major categories: those possessing ROS-scavenging activities (e.g., SOD and CAT) and those exhibiting ROS-generating activities (e.g., OXD, POD) [104–106]. The former are commonly utilized in treating diseases involving oxidative stress and inflammatory responses, while the latter are often applied in cancer therapy and antibacterial treatment. Beyond the above two categories of enzyme-like activities, MOF nanozymes with phosphatase-like activity have also been reported. They are primarily applied in constructing biosensing platforms. In this chapter, we introduce the main enzyme-like activities of MOF nanozymes, providing discussions on how the structure of MOF nanozymes influences their enzyme-like activities, such as how unsaturated metal sites, ligand participation, and pore confinement effects affect catalytic activity and selectivity. The Michaelis–Menten constant (*K*_m_) and maximum reaction rate (*V*_max_) of representative MOF nanozymes are summarized in Table 2.

**Table 2:** Summary of the key catalytic parameters of representative MOF nanozymes

MOF nanozyme	*K*_m_	*V*_max_	References
ZrFe-MOF@PtNPs	0.04913 mM	6.65 × 10^–7^ M s^−1^	[185]
Cu-Pd@MIL-101	2.5 × 10^–2^ mM	8.3 × 10^–8^ M s^−1^	[111]
MIL-88B-Fe/Zn	4.70 mM	9.20 × 10^–8^ M s^−1^	[197]
D/P@ZUCO	1.061 μM	139.4 nM s^−1^	[200]
ES@Cu(II)-MO	0.31 mM	2.78 × 10^−8^ M s^−1^	[190]
RCL@Pd@CuZ	2811 mM	3.866 μM s^−1^	[201]
UIO-66-Au	8.13 mM	4.16 × 10^−8^ M s^−1^	[147]
MOF-919	0.14 mM	4.98 × 10^−7^ M s^−1^	[202]
Ti_3_C_2_ MXene/Fe-MOFs	0.5 mM	5.6 × 10^−7^ M s^−1^	[203]
GATC	1.09 mM	2.57 × 10^–8^ M s^−1^	[204]
DSAM	0.81 mM	1.58 × 10^−8^ M s^−1^	[172]
Fe_3_O_4_@MOF@Au	0.24 mM	10.1 × 10^–8^ M s^−1^	[205]
Co-TCPP(Fe)	22.595 mM	1114 × 10^–8^ M s^−1^	[206]
ZnCo-ZIFs@MIL-101(Fe)	1.96 mM	10.36 × 10^–8^ M s^−1^	[207]
ZnIrOx/ZnIrMOFs	1.31 mM	6.54 × 10^–7^ M s^−1^	[208]
Cu_2_O/Au-Pt@MOF	22.40 mM	4.05 × 10^–8^ M s^−1^	[138]
Ru@U6-Ru/Pt	6.87 mM	5.27 × 10^–8^ M s^−1^	[182]
FeTDC	0.1 mM	1.98 × 10^–8^ M s^−1^	[209]
Fe_3_O_4_@CeO_2_/Tb-MOF	0.41 mM	2.17 × 10^−7^ M s^−1^	[18]

### SOD-Like Activity

Superoxide anions (·O_2_^−^) are pivotal ROS inside the human body, which exhibit high chemical reactivity and serve as the initiating factor in ROS cascade reactions. Excessive accumulation of ·O_2_^−^ tends to induce cellular damage via different mechanisms, such as ​lipid peroxidation, DNA mutagenesis, and protein inactivation [107, 108]. Functioning as a core defense enzyme, SOD mediates the catalytic disproportionation of ·O_2_^−^ into H_2_O_2_ and O_2_ [109]. MOF nanozymes exhibiting SOD-like activity are able to disrupt oxidative damage chains by accelerating this catalytic process, thereby emerging as critical therapeutic agents for diseases related to oxidative stress and inflammation [110]. Moreover, the generated H_2_O_2_ can participate in transition metal-mediated Fenton reactions, producing hydroxyl radicals (·OH) that induce biomolecular oxidative damage. In addition, oxygen production can alleviate intrinsic hypoxia in solid tumors, thereby enhancing therapeutic efficacy [111]. Therefore, MOF nanozymes with SOD-like activity are also widely employed in chemodynamic therapy (CDT) of tumor. The SOD-like activity of MOF nanozymes relies on metal centers to undergo redox cycling for the disproportionation of superoxide anions ·O_2_^−^. Inspired by the function and structure of natural SODs, Liu et al. developed a cerium-based MOF nanozyme with SOD-like activity for ionizing radiation protection [112]. The SOD-like catalytic mechanism of Ce-MOFs involves a cycle between Ce(IV) and Ce(III). In addition, it was found that the SOD-like activity of high-valence Ce(IV) nanozyme was more effective than that of Ce(III).

### CAT-Like Activity

At low concentrations, H_2_O_2_ serves as a redox signaling mediator involved in maintaining cellular homeostasis, whereas excessive H_2_O_2_ accumulation leads to oxidative stress, DNA damage, and metabolic dysregulation. Like SOD, CAT is another important antioxidant enzyme. SOD and CAT form a cascade catalytic system, efficiently converting H_2_O_2_ into harmless H_2_O and O_2_ via dismutation reactions, thereby maintaining redox homeostasis and preventing oxidative damage [113, 114]. Similarly, MOF nanozymes with CAT-like activity are often utilized to manage diseases involving oxidative stress and inflammatory responses. Many MOF nanozymes exhibit CAT-like activity, and they can be applied in photodynamic therapy (PDT), sonodynamic therapy (SDT), or radiotherapy to relieve tumor hypoxia [115]. The CAT-like activity of MOF nanozymes primarily originates from the metal nodes or clusters. It should be noted that the level of CAT-like activity is not solely determined by the type of metal nodes, but rather by the synergistic effect of the MOF’s overall structure [116]. For instance, a hydrophobic pore environment facilitates the rapid desorption of non-polar O_2_ products, preventing bubble blockage of active sites and thereby maintaining high catalytic efficiency. Additionally, a mesoporous structure promotes the rapid diffusion of H_2_O_2_ reactants and H_2_O products, reducing mass transfer limitations. Ligands also play a regulatory role in modulating CAT-like activity. They can indirectly affect the electron density of metal nodes through electronic effects, fine-tuning their redox potentials. Electron-donating groups (e.g., -NH_2_) can enhance the electron density of metal nodes and optimize their ability to activate H_2_O_2_.

### OXD-Like Activity

OXD is a critical subclass of oxidoreductases that catalyzes substrate oxidation by directly utilizing O_2_ as the electron acceptor, producing H_2_O_2_ or H_2_O [117]. It can be categorized into multiple distinct subtypes (e.g., glucose oxidase (GOx), polyphenol oxidase, urate oxidase), and they exhibit high substrate specificity [118, 119]. MOF nanozymes with OXD-like activity are widely applied in biosensing, because they can oxidize colorless substrates (e.g., 3,3’,5,5’-tetramethylbenzidine (TMB), o-phenylenediamine (OPD)) into colored products [120, 121]. If target analytes inhibit OXD enzyme activity, their concentrations can be quantified colorimetrically according to the standard calibration curve. Additionally, MOF nanozymes with GOx-like activity have been extensively studied in tumor therapy. They can oxidize glucose at the tumor site to generate H_2_O_2_. The produced H_2_O_2_ can either undergo Fenton reactions with metal ions to yield ·OH for tumor CDT or produce oxygen to alleviate tumor hypoxia under the catalytic action of CAT [122]. The OXD-like activity of MOF nanozymes is jointly determined by the metal nodes, organic ligands, and pore structures. Metals with appropriate redox potentials serve as the foundation for efficient OXD-like activity. Cu(II)/Cu(I) and Co(III)/Co(II) are the most widely studied systems, whose facile valence changeability makes them excellent “transfer stations.” The degree of coordinative unsaturation of metal nodes directly affects their ability to adsorb O_2_ and the efficiency of electron transfer. Creating more unsaturated sites during synthesis is a key strategy to enhance activity [123]. Ligands with large π-conjugated systems (e.g., porphyrins, phthalocyanines) or those bearing electron-donating/withdrawing groups can significantly modulate the electron density of metal nodes, thereby altering their Fermi level positions and optimizing the energy level matching with the molecular orbitals of O_2_ to accelerate electron transfer. Additionally, suitable pore sizes facilitate the rapid diffusion and enrichment of O_2_ and organic substrate molecules, increasing local reaction concentrations and rates.

### POD-Like Activity

As a pivotal oxidoreductase, POD harnesses H_2_O_2_ or organic peroxides as electron acceptors to oxidize broad-spectrum substrates [124]. Under high-concentration H_2_O_2_ conditions, POD can mediate the catalytic conversion of H_2_O_2_ into ·OH for tumor CDT [125]. Therefore, MOF nanozymes with POD-like activity are often applied together with those with GOx-like activity in synergistic tumor treatment. The generated ·OH also exhibits potent antibacterial effects, which can be used for wound sterilization. Similar to OXD-like activity, MOF nanozymes possessing POD-like activity can cause the oxidation of colorless substrates into colored products in the presence of H_2_O_2_. Therefore, they can be utilized to construct biosensing platforms for target molecule detection. The POD-like activity of MOF nanozymes primarily originates from the process in which transition metal nodes with variable valence (e.g., Fe, Cu, Mn, Co) catalyze the homolytic cleavage of H_2_O_2_ to generate ·OH [126]. Similar to the OXD-like activity, the POD-like activity of MOF nanozymes is predominantly governed by three key structural factors: the intrinsic activity of the metal nodes, the electronic effects of the organic ligands, and the confinement effects of the pore channels.

GPx is one prominent subclass among the diverse POD family. It is a selenium-dependent antioxidant enzyme which can catalyze the reduction of peroxides, thereby mitigating oxidative damage to cells. Similar to CAT, GPx can decompose H_2_O_2_ into water, while this process requires GSH participation as electron donor. In the human body, high concentrations of H_2_O_2_ are primarily scavenged by CAT, whereas low concentrations of H_2_O_2_ are handled by GPx [127]. This enables efficient H_2_O_2_ clearance through complementary mechanisms. Collectively, SOD-, CAT-, and GPx-like activities of MOF nanozymes form a cascade catalytic defense system, synergistically scavenging ROS and maintaining redox homeostasis [128]. Therefore, MOF nanozymes with these enzyme-like activities are widely applied in oxidative stress and inflammation-related disease therapy.

### Phosphatase-Like Activity

Phosphatases are a class of hydrolytic enzymes that catalyze substrate dephosphorylation. They hydrolyze phosphoester bonds and generate inorganic phosphate as well as free hydroxyl groups. These enzymes regulate signal transduction, metabolic balance, and cell cycle processes in organisms, and they also demonstrate significant application value in biosensing [129]. MOF nanozymes with phosphatase-like activity can be utilized to construct biosensing platforms for target molecule detection. For instance, some biomolecules themselves lack color, while they produce colored substances under the hydrolysis catalyzed by MOF nanozymes with phosphatase-like activity. This mechanism can be applied to the detection of target molecules, where a quantitative relationship exists between their concentration and color intensity of the detection system [130]. The colorimetric method is the most commonly used detection mode, and fluorescence as well as electrochemical methods are also widely employed. In contrast to the oxidoreductases introduced above, phosphatases exhibit hydrolase activity, with their catalytic mechanism shifting from electron transfer to nucleophilic attack or acid–base catalysis. Phosphatase activity relies on the electrophilic activation of phosphorus atoms by Lewis acidic metals, along with the nucleophilic attack on the phosphorus atom [131]. The phosphatase-like activity of MOF nanozymes primarily stems from the electrophilic activation of phosphorus atoms in phosphate esters by their Lewis acidic metal nodes (e.g., Zr, Ce, Hf), along with facilitating the hydrolysis reaction by the pore microenvironment.

## Biomedical Applications of MOF Nanozymes

Due to the diverse enzyme-like activities, MOF nanozymes have great application potential in the biomedical field. The intrinsic enzyme-like activities they possess determine their specific application scenarios. As mentioned above, MOF nanozymes with ROS-scavenging activities are often employed to treat oxidative stress and inflammation-related diseases, and those with ROS-generating activities can be applied in tumor therapy and antimicrobial treatment. In addition, MOF nanozymes with phosphatase-like activity are often applied in biosensing. In this section, the primary biomedical applications of MOF nanozymes are comprehensively reviewed, including four key domains: tumor therapy, oxidative stress- and inflammation-related disease therapy, antimicrobial therapy, and biosensing. The working mechanisms of MOF nanozymes in different application cases are highlighted.

### Tumor Therapy

Tumors represent a major category of diseases confronting humanity, posing severe threats to human health and life. Though conventional treatments (e.g., surgery, radiotherapy, and chemotherapy) show certain therapeutic efficacy, they still have some limitations, including high recurrence rates and severe side effects [132]. The tumor microenvironment (TME) is characterized by hypoxia, mild acidity, overproduction of H_2_O_2_ and antioxidants [133]. Accumulating evidence shows that the regulation of TME can improve tumor therapy efficiency. In recent years, nanozyme-based catalytic therapy has been regarded as a promising strategy for site-specific tumor treatment, leveraging its dual capacity to reprogram the immunosuppressive TME and catalyze in situ generation of cytotoxic ROS, ultimately inducing cancer cell death. In addition, MOF nanozymes can serve as carriers to load drug molecules, enabling the synergistic combination of multiple therapeutic modalities. This section reviews how MOF nanozymes exert unique functions in different modes of tumor therapy, including CDT, PDT, and SDT (Table 3). The core working mechanism of nanozymes in these therapies is generating cytotoxic ROS to induce cancer cell elimination by direct oxidative damage, induction of programmed cell death, or microenvironment disruption [134, 135]. In the designed MOF nanozyme systems, synergistic effects among multiple enzyme-like activities are often incorporated, with each fulfilling its specific role.

**Table 3:** Summary of MOF nanozymes for tumor treatment

Treatment mode	MOF system	Enzyme activity combination	Tumor inhibition rate	Safety dosage	References
CDT	Cu-Pd@MIL-101	SOD/POD	80.9%	20 mg kg^−1^	[111]
	Au/FeMOF@CPT	GOx/POD	85.6%	1 mg kg^−1^ (CPT)	[137]
	Cu_2_O/Au–Pt@MOF	CAT/GOx/POD	–	10 mg mL^−1^	[138]
	Pd/Cu SAzyme@Dzy	POD	65%	5 mg kg^−1^	[210]
	CA@MOF-808(Zr/Fe)-AuNP-Mn-PEG	POD/GOx	–	–	[211]
PDT	CMZM	SOD/CAT/POD/GSHOx	98.9%	20 mg kg^−1^	[141]
	Ce6@MIL-100	POD	Nearly 100%	3 mg kg^−1^ (Ce6)	[36]
	Pt-carbon nanozyme	CAT	More than 90%	10 mg kg^−1^	[212]
	ICG-PtMGs@HGd	CAT	–	1 mg kg^−1^ (ICG)	[213]
	PCN222-Mn@GOx/HA	CAT	–	5 mg kg^−1^	[214]
SDT	Au/TF-MOF	POD/CAT/GOx	–	–	[146]
	DOX@PCN-224/Pt	CAT	84.4%	150 μL, 100 μg mL^−1^	[145]
	UIO-66-Au	POD/CAT	93.2%	100 µL, 1 mg mL^−1^	[147]
	RuO_2_@Zr-MOF	CAT/POD	100%	4 mg kg^−1^	[215]

#### CDT

**Fig. 4:**
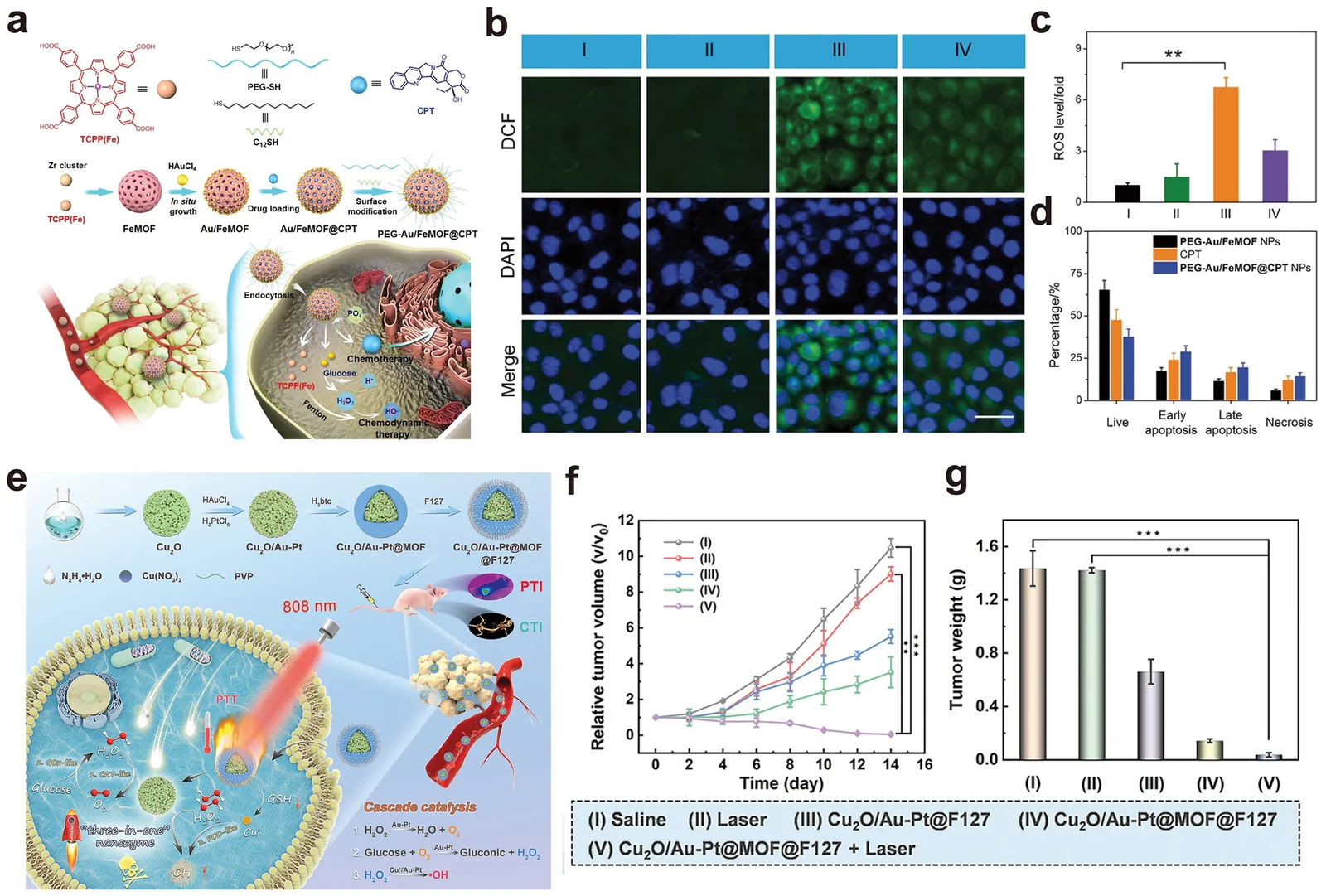
Applications of MOF nanozymes for tumor CDT. **a** Preparation method of the PEG-Au/FeMOF@CPT nanomedicine and the mechanism for tumor treatment. **b** CLSM images of HepG2 cells stained with DCFH-DA probe. **c** Quantitative analysis of ROS level. **d** Cell apoptosis analyzed by Annexin-V/PI. Figures a to d are reproduced with permission [137]. Copyright 2020, Wiley–VCH. **e** Preparation method and working mechanism of Cu_2_O/Au–Pt@MOF@F127 for tumor treatment. **f** Tumor growth curves of mice in different groups. **g** Average tumor weights in different groups. Figures **e** to **g** are reproduced with permission [138]. Copyright 2023, Wiley–VCH

CDT operates through Fenton or Fenton-like reactions catalyzed by transition metal ions (e.g., Fe^2+^, Mn^2+^, Cu^+^) to convert H_2_O_2_ into highly toxic ·OH, thereby killing cancer cells [136]. Nanozymes possessing both SOD- and POD-like activities are highly suitable for CDT, as they can cascade catalyze the conversion of ·O_2_^−^ into H_2_O_2_, which is then further catalyzed into ·OH. This process significantly enhances the efficacy of CDT. Yang et al. constructed such nanozyme (Cu-Pd@MIL-101) for tumor CDT [111]. Upon entering the tumor site, the nanozyme converts ·O_2_^−^ into H_2_O_2_ by SOD-like activity. Then, its POD-like activity catalyzes the generated H_2_O_2_ into ·OH, inducing lipid peroxidation of tumor cells. The POD-like activity of MOF nanozymes can also synergize with GOx-like activity to enhance the efficacy of CDT, because the GOx-like activity can catalyze glucose to produce H_2_O_2_, supplying the chemical fuel for Fenton reactions. As shown in Fig. 4a, Ding et al. developed Au NPs-decorated MOF platform for cancer CDT [137]. Au NPs with GOx-like activity catalyze the oxidation of glucose to produce H_2_O_2_, which is subsequently harnessed by TCPP(Fe) to drive site-specific Fenton reactions for ·OH generation. The fluorescent detection using 2,7-Dichlorofluorescin diacetate (DCFH-DA) probe confirmed a 6.74-fold elevation in intracellular ROS levels (Fig. 4b, c), leading to significant cell apoptosis and necrosis (Fig. 4d). GOx requires oxygen to exert its catalytic activity, while solid tumor sites are often hypoxic. To address this limitation, introducing CAT-like activity into the GOx/POD system is an ideal strategy. Cheng et al. constructed Au/Pt NPs-encapsulated MOF nanozyme for augmented CDT [138]. Cu_2_O NPs served as a structural scaffold for anchoring ultrafine Au-Pt NPs while simultaneously supplying copper for in situ growth of a porous MOF coating (Fig. 4e). Once inside tumor cells, GSH-triggered degradation of the MOF shell concurrently depletes GSH reserves and liberates Cu^+^ ions for Fenton-like reactions. The exposed Au-Pt nanozyme then demonstrates cascaded catalytic activities: It decomposes H_2_O_2_ into O_2_ via CAT-like activity, subsequently facilitates H_2_O_2_ production through GOx-like activity, and finally converts H_2_O_2_ into ·OH via POD-like activity, thereby enabling self-enhanced CDT. The “three-in-one” MOF nanozyme significantly inhibited the growth of tumor volume and weight in U87 xenograft models (Fig. 4f, g).

#### PDT

PDT leverages photosensitizing agents activated by specific-wavelength laser to generate cytotoxic ROS within malignant tissues. It triggers selective tumor destruction through apoptosis or necrosis while enabling precise spatiotemporal control [139]. Due to oxygen supply–demand imbalance driven by accelerated tumor cell proliferation and compounded by dysfunctional vasculature impairing oxygen delivery, hypoxia is a hallmark pathological feature of solid tumors, and this often undermines the therapeutic efficacy of PDT [140].

**Fig. 5:**
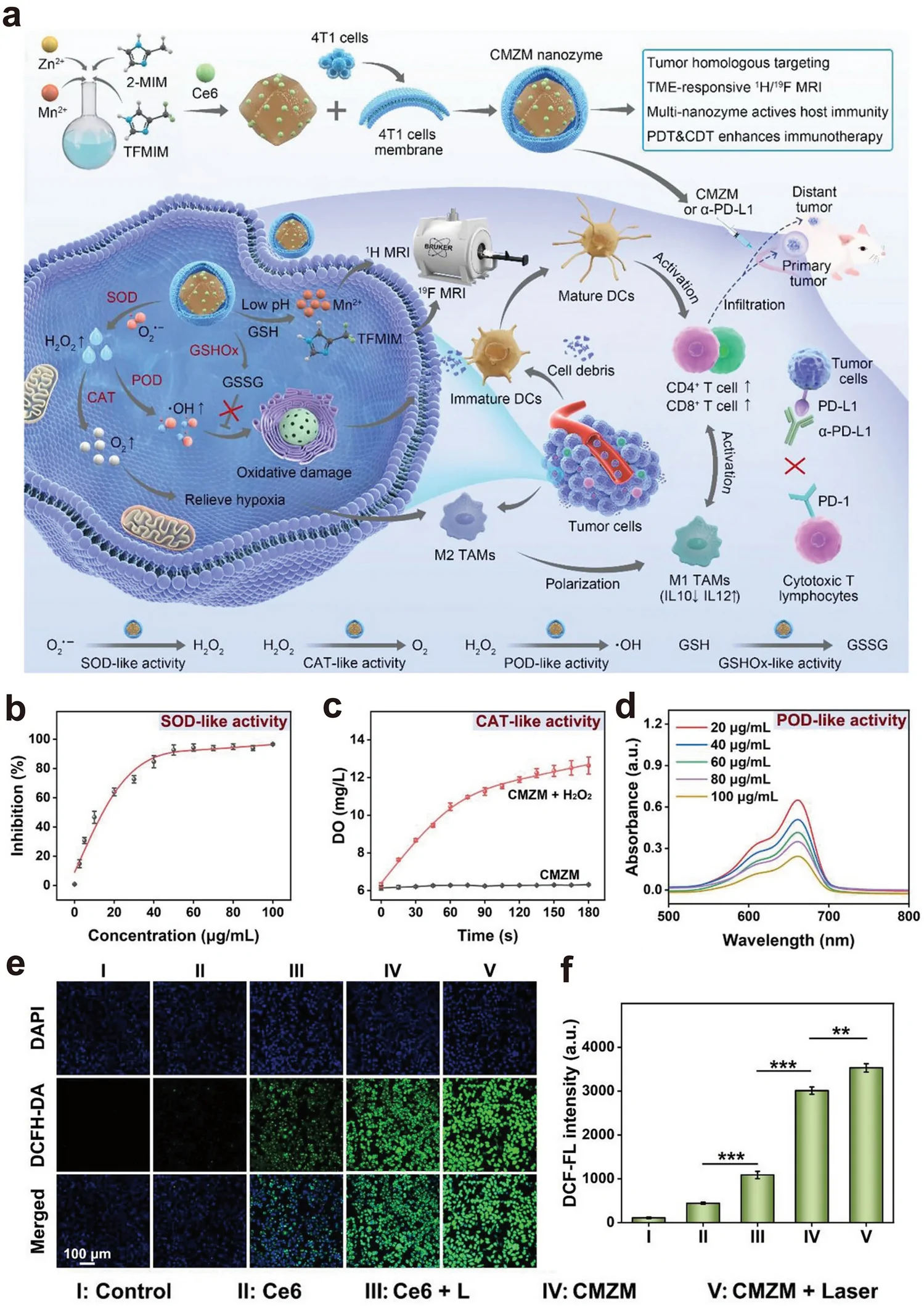
Applications of MOF nanozymes for tumor PDT. **a** Synthesis process of the bioinspired MOF nanozyme and the mechanism for tumor treatment. **b** Evaluation of SOD-like activity of the prepared MOF nanozyme. **c** Evaluation of CAT-like activity of the prepared MOF nanozyme. **d** Evaluation of POD-like activity of the prepared MOF nanozyme. **e** CLSM images of ROS detected by DCFH-DA probe inside 4T1 cells. **f** Quantitative analysis of DCF fluorescence intensity. Figures a to f are reproduced with permission [141]. Copyright 2023, Wiley–VCH

MOF nanozymes with CAT-like activity can decompose the abundant H_2_O_2_ within TME into O_2_, therefore augmenting oxygen supply for efficient PDT. As shown in Fig. 5a, Wang et al. developed MOF nanozyme with multiple enzyme-like activities for synergistic CDT, PDT, and immunotherapy of tumor [141]. The prepared MOF nanozyme was demonstrated to exhibit SOD-, CAT-, and POD-like activities (Fig. 5b-d). After internalization by tumor cells, it leverages the SOD-like activity to convert ·O_2_^−^ into H_2_O_2_. Subsequently, it decomposes H_2_O_2_ into O_2_ via the CAT-like activity, alleviating tumor hypoxia while supplying oxygen for PDT. Furthermore, it can directly transform H_2_O_2_ into toxic ·OH by the POD-like activity, ultimately achieving synergistic integration of multiple therapeutic modalities. DCFH-DA probe further confirmed that the prepared MOF nanozyme can generate abundant ROS inside tumor cells under 650 nm laser irradiation (Fig. 5e, f). Apart from hypoxia, overexpressed antioxidants (e.g., GSH) in TME also compromise PDT efficiency. To address this problem, Pan et al. developed a bimetallic MOF nanozyme with both CAT- and glutathione oxidase (GSHOx)-like activities for TME regulation and enhanced PDT [142]. The consuming of GSH by MOF nanozyme made the cancer cells more sensitive to ROS and further improved the antitumor efficacy.

#### SDT

**Fig. 6:**
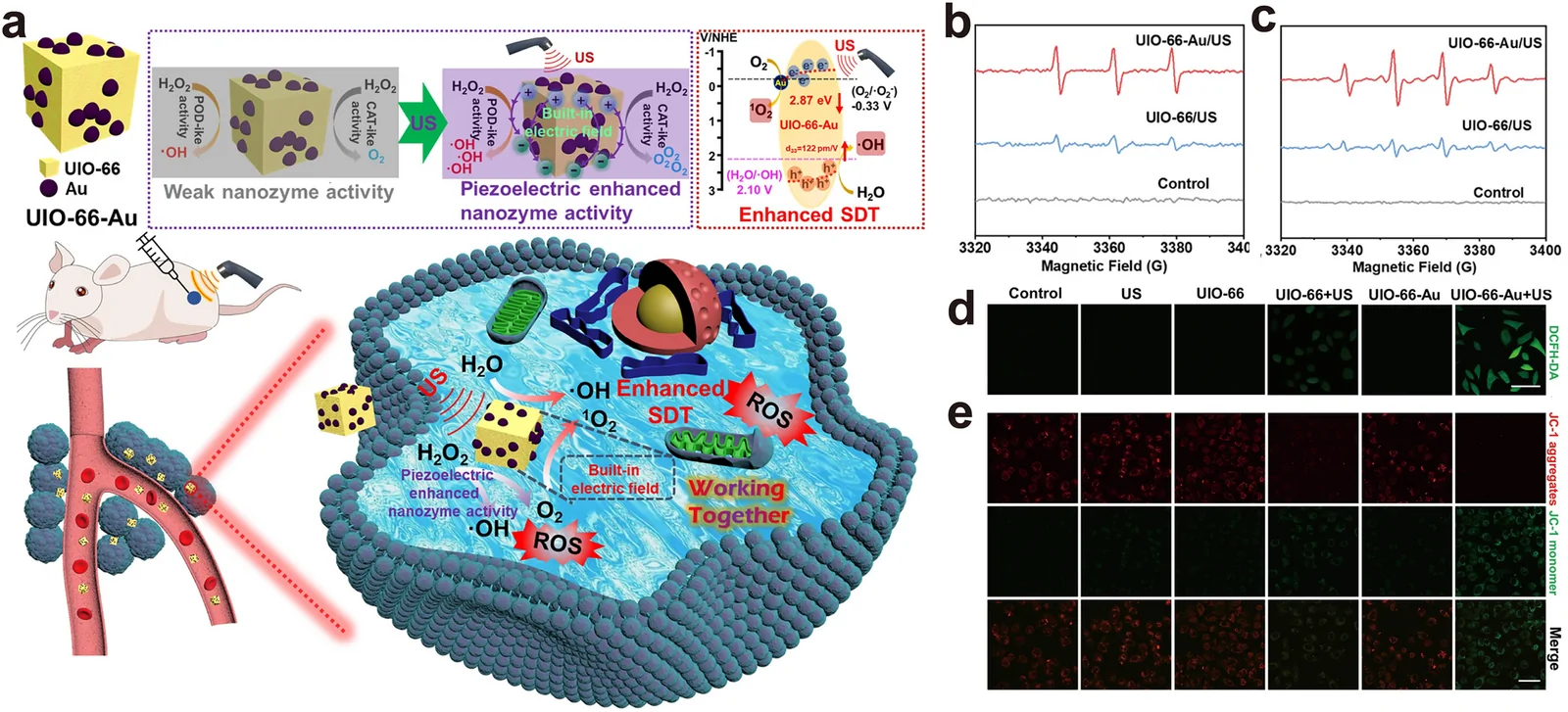
Applications of MOF nanozymes for tumor SDT. **a** Mechanism of UIO-66-Au NPs for tumor SDT. **b** Detection of ^1^O_2_ in different groups under ultrasonic irradiation. **c** Detection of ·OH in different groups under ultrasonic irradiation. **d** CLSM images of MCF-7 cells stained with DCFH-DA probe after different treatments. **e** CLSM images of MCF-7 cells stained with JC-1 probe after different treatments. Figures a to e are reproduced with permission [147]. Copyright 2023, American Chemical Society

SDT utilizes low-intensity ultrasound to activate tumor-accumulating sonosensitizers, triggering localized generation of cytotoxic ROS to induce cancer cell death [143]. It offers superior tissue penetration compared to light-dependent therapy modalities, enabling precise targeting of deep-seated malignancies while mitigating the intrinsic constraints and off-target effects of photoirradiation-based treatments [144]. MOF nanozymes enhance SDT efficacy primarily through two interconnected mechanisms, with the first focusing on modulating the TME and enhancing catalytic activity. Nanozymes exhibiting CAT-like activity can decompose excess H_2_O_2_ in TME to generate O_2_, thereby supplying the essential O_2_ substrate required for SDT. In addition, nanozymes with SOD-like activity can convert ·O_2_^−^ produced during SDT into H_2_O_2_ and O_2_. The generated H_2_O_2_ can then serve as a substrate for POD-like activity to generate ·OH, while the O_2_ can be applied in SDT. This synergy establishes an efficient network for the generation and transformation of ROS. On the other hand, MOF nanozymes weaken the body’s defense against ROS and achieve sensitization of SDT. For instance, nanozymes possessing GPx-like activity can deplete GSH, the most critical antioxidant within tumor cells. This weakens the cellular capacity to scavenge ROS, thereby enhancing the efficacy of SDT. Although SDT demonstrates distinct advantages in tumor treatment, its actual therapeutic efficacy is often limited by multiple factors, such as hypoxia in TME and insufficient generation of ROS. To address this problem, Ren et al. decorated Pt NPs on PCN-224 MOF to improve oxygen evolution for enhanced SDT [145]. The immobilized Pt NPs enabled cascade catalytic conversion of intratumoral H_2_O_2_ into O_2_, simultaneously driving ultrasound-triggered production of cytotoxic ^1^O_2_. Adopting a similar strategy, Xu et al. reported self-healing hydrogel embedding Au NPs-decorated Ti/Fe bimetallic MOF nanozyme for multimodal synergistic therapy of tumor [146]. Au NPs in the nanozyme can consume glucose to achieve ST and generate abundant H_2_O_2_. Then, the nanozyme can convert H_2_O_2_ into ·OH for CDT by the POD-like activity. In addition, the CAT-like activity can improve the generation of O_2_ to support SDT. Apart from utilizing CAT-like activity to catalyze oxygen generation for SDT, MOF nanozymes fabricated from piezoelectric materials can themselves serve as sonosensitizers. As shown in Fig. 6a, Cai et al. prepared UiO-66 MOF-based sonosensitizer for tumor SDT [147]. UiO-66 MOF generated a built-in electric field under US irradiation, which promoted the production of ^1^O_2_ and ·OH (Fig. 6b, c). The DCFH-DA probe detected abundant ROS inside MCF-7 cells treated with UIO-66-Au NPs together with US (Fig. 6d). Furthermore, detection using the JC-1 probe showed that this treatment also caused a significant reduction in mitochondrial membrane potential (Fig. 6e), demonstrating the ideal therapeutic efficacy.

### Oxidative Stress and Inflammation-Related Disease Therapy

ROS are a class of highly reactive oxygen-containing substances in living organisms, primarily including ·O_2_^−^, H_2_O_2_, ·OH, and ^1^O_2_. They play a dual role in physiological and pathological processes: At low concentrations, they act as crucial signaling molecules regulating cellular functions, whereas excessive accumulation elicits oxidative stress [148]. In addition, overactivation of oxidative stress induces inflammatory reactions, while the resulting inflammation further amplifies oxidative stress, forming a mutually reinforcing vicious loop [149]. Thus, maintaining the equilibrium between ROS production and elimination is crucial for minimizing oxidative damage to the human body. MOF nanozymes with ROS-scavenging activities (e.g., SOD-, CAT-, and GPx-like activities) show great promise for treatment of oxidative stress and inflammation-related diseases [150]. It is noteworthy that MOF systems combining diverse enzyme-like activities can simulate complete natural enzymatic antioxidant pathways, thereby enabling more efficient clearance of ROS.

**Fig. 7:**
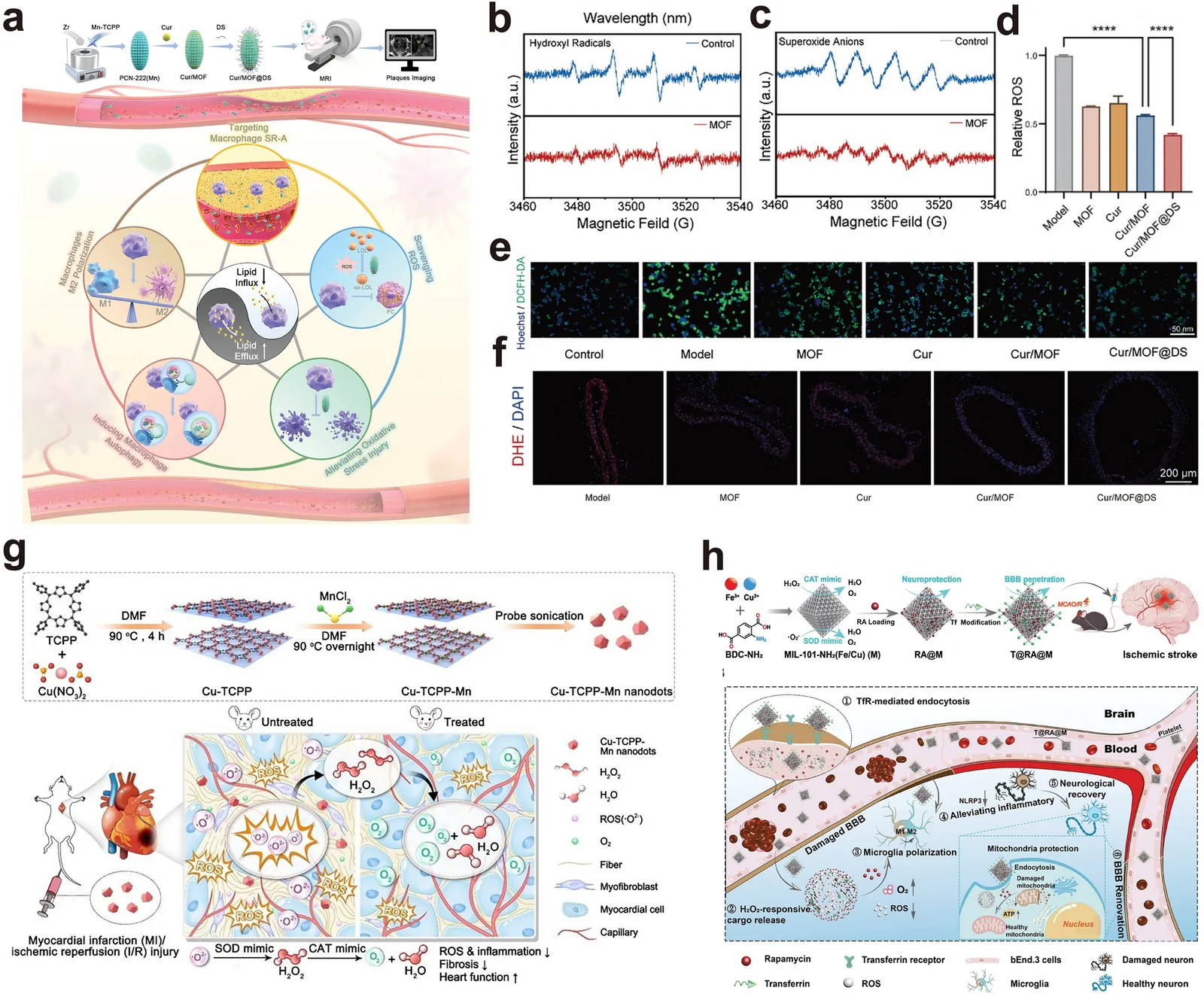
Applications of MOF nanozymes for cardiovascular and cerebrovascular disease therapy. **a** Synthesis process of Cur/MOF@DS for targeted AS treatment and the illustration of potential mechanisms. **b** ESR spectroscopy evaluations of the radical scavenging efficacy of MOF nanozyme against ·OH. **c** ESR spectroscopy evaluation of the radical scavenging efficacy of MOF nanozyme against O_2_·⁻. **d** Quantitative analysis of relative ROS levels. **e** CLSM images of Raw264.7 cells stained with DCFH-DA probe after different treatments. **f** DHE staining of aortic tissues in different groups, with red fluorescence representing ROS labeled with DHE. Figures **a** to **f** are reproduced with permission [152]. Copyright 2024, Wiley–VCH. **g** Synthesis of Cu-TCPP-Mn nanozyme for myocardial injury treatment. Reproduced with permission [153]. Copyright 2023, Ivyspring International Publisher. **h** Synthetic protocol of T@RA@M nanozyme and the working mechanism for targeted IS therapy. Reproduced with permission [154]. Copyright 2024, Wiley–VCH

**Fig. 8:**
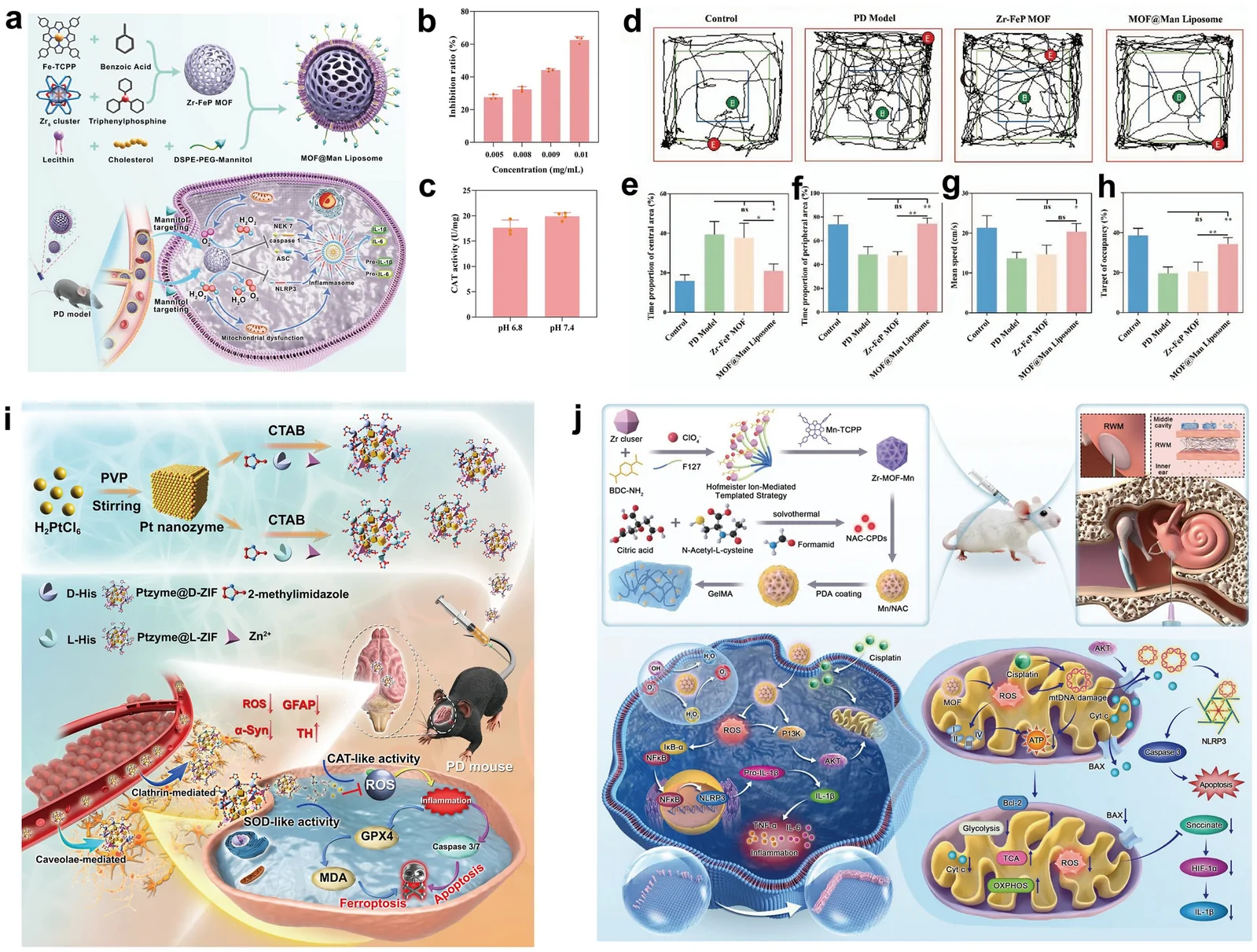
Applications of MOF nanozymes for neurological disease therapy. **a** Preparation method of MOF nanozyme and the working mechanism for PD therapy. **b** Detection of the SOD-like activity of the nanozyme under different conditions. **c** Detection of the CAT-like activity of the nanozyme at different conditions. **d** Results of open-field tests of PD model mice after treatment. **e** Time spent in the central zone in open-field test. **f** Time spent in the peripheral zone in open-field test. **g** Mean swimming speed in the target quadrant of PD mouse models post-treatment with nanozyme systems in Morris water maze test. **h** Relative time spent in the target quadrant of PD mouse models post-treatment with nanozyme systems in Morris water maze test. Figures **a** to **h** are reproduced with permission [155]. Copyright 2024, Wiley–VCH. **i** Preparation method of MOF nanozyme and the therapeutic mechanism in PD model mice. Reproduced with permission [156]. Copyright 2023, Springer Nature. **j** Preparation method and the mechanisms of Gel/Mn/NAC/PDA in protecting cisplatin-induced hearing loss. Reproduced with permission [157]. Copyright 2024, Wiley–VCH

MOF nanozymes have demonstrated multifaceted therapeutic efficacy against cardiovascular and cerebrovascular diseases. They enable targeted ROS scavenging, modulate the inflammatory microenvironment, and promote tissue repair [151]. As shown in Fig. 7a, Lv et al. prepared curcumin (Cur)- and dextran sulfate (DS)-loaded PCN-222(Mn) nanozyme for targeted atherosclerosis (AS) treatment [152]. It was demonstrated that the nanozyme displayed noteworthy CAT-like activity. In addition, electron spin resonance (ESR) analyses revealed that the nanozyme can effectively eliminate ·OH and ·O_2_^−^ (Fig. 7b, c). Furthermore, the ROS-scavenging capacity of the nanozyme was confirmed by DCFH-DA probe (Fig. 7d, e). It was found that the relative ROS fluorescence intensity decreased from 1.7 in the model group to approximately 1.4 in the MOF nanozyme-treated group, corresponding to a reduction of about 17.65%. In vivo anti-AS efficacy of the nanozyme was evaluated in AS model mice. After treatment, the ROS level in aortic frozen sections was stained with dihydroethidium (DHE) probe. As shown in Fig. 7f, Cur/MOF@DS treatment significantly attenuated DHE fluorescence, with the relative ROS intensity decreased to around 40%. In addition, Xiang et al. developed a bimetallic nanozyme platform for protection of myocardial injury [153]. Cu-TCPP was prepared by solvothermal method, then manganese ions were incorporated into Cu-TCPP frameworks to construct bimetallic Cu-TCPP-Mn nanosheets, which were subsequently processed into nanodots via ultrasonic fragmentation (Fig. 7g). In vitro evaluations revealed enhanced SOD/CAT-mimetic activities in these nanodots compared to Cu-TCPP, which can be ascribed to the Mn-N active sites. For treatment of brain diseases, effective blood–brain barrier (BBB) penetration remains a big challenge of nanomedicines. Post-synthetic modification of MOF nanozymes can enhance their targeting capability, thereby improving therapy efficacy. As shown in Fig. 7h, Chen et al. developed MOF nanozyme with transferrin modification (T@RA@M) for targeted ischemic stroke therapy [154]. In vitro assessments demonstrated that T@RA@M presented both SOD- and CAT-like activities. In vivo studies confirmed the remarkable capability of MOF nanozyme in traversing the BBB due to the active targeting effect of Tf modification. In addition, mannitol (Man) modification can also improve the BBB penetration efficiency of nanozymes. As shown in Fig. 8a, Li et al. developed Zr-FeP MOF nanozyme for neuroinflammatory regulation in PD therapy [155]. The nanozyme with both SOD- and CAT-like activities (Fig. 8b, c) can exert antioxidant and anti-inflammatory functions to alleviate oxidative damage to nerve cells. Notably, post-intervention assessments with open-field and Morris water maze tests demonstrated that tail vein injection of the nanozyme significantly ameliorated the behavioral and associated mobility impairments in PD model mice (Fig. 8d-h). In addition, Jiang et al. reported chiral-dependent BBB transendocytosis MOF nanozyme with excellent antioxidant activity for PD treatment [156]. The D-histidine-functionalized nanozyme realized superior brain accumulation in PD model mice, which can be ascribed to extended plasma half-life and diverse BBB transendocytosis pathways (Fig. 8i). MOF nanozymes can also be applied in hearing loss therapy due to the outstanding antioxidant properties [157]. Loading them into hydrogel extended the retention time in the middle ear, thereby improving the therapy efficacy (Fig. 8j).

**Fig. 9:**
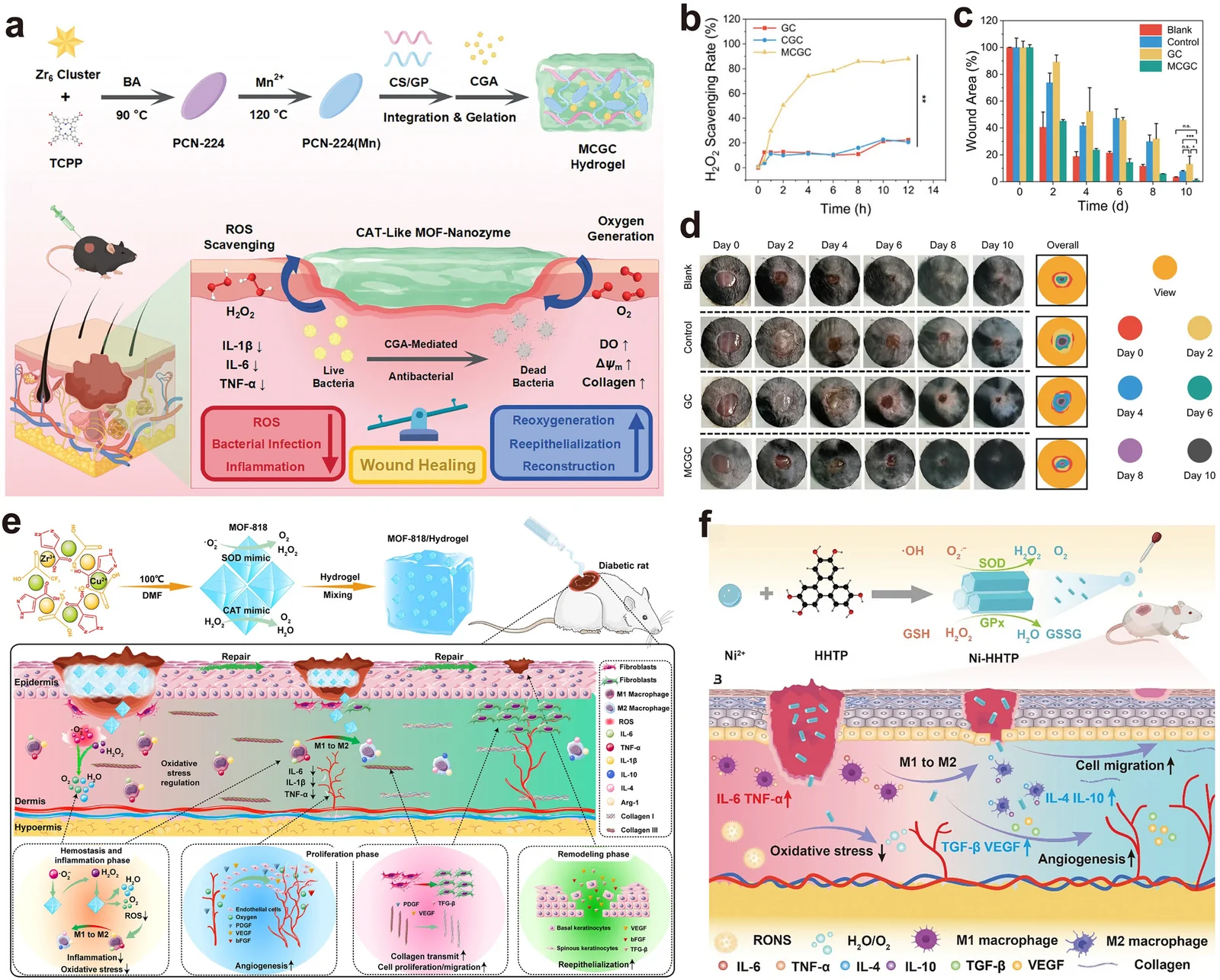
Applications of MOF nanozymes for promoting wound healing. **a** The synthetic routes of PCN-224(Mn) MOF nanozyme-loaded hydrogel and the mechanism for promoting the healing of diabetic wounds. **b** H_2_O_2_ scavenging rates of different formulations of hydrogels. **c** Statistics of wound areas treated with or without hydrogels. **d** The wound-healing tracks of wound areas treated with or without the hydrogels. Figures a to d are reproduced with permission [159]. Copyright 2024, Wiley–VCH. **e** The preparation method of MOF-818-embedded thermosensitive hydrogel and the mechanisms in promoting diabetic wound healing. Reproduced with permission [160]. Copyright 2022, American Chemical Society. **f** Synthesis process and therapeutic mechanisms of Ni-HHTP nanozyme for diabetic wound treatment. Reproduced with permission [161]. Copyright 2024, Wiley–VCH

Wounds caused by mechanical injuries or systemic diseases are common clinical conditions that impair normal life of patients. Excessive ROS at the wound sites may induce persistent inflammatory responses, thereby impeding the healing progression [158]. This is particularly evident in chronic wounds such as diabetic foot ulcers. Wei et al. prepared bimetallic MOF nanozyme-embedded hydrogels for the therapy of infected diabetic wounds [159]. The MOF nanozyme promotes wound healing primarily through dual synergistic mechanisms: First is scavenging excessive ROS, and second is leveraging the CAT-like activity to decompose local H_2_O_2_, thereby alleviating hypoxia condition at the wound site (Fig. 9a). In vitro evaluations showed that H_2_O_2_ scavenging rates of the nanozyme reached 87.9% within 12 h and 97.7% within 24 h, respectively, demonstrating the excellent CAT-like activity (Fig. 9b). The wound-healing capacity of the nanozyme was further confirmed in a mouse model of infected diabetic wounds (Fig. 9c, d). In addition, Chao et al. developed MOF-818 nanozyme-loaded thermosensitive hydrogel for diabetic chronic wounds therapy (Fig. 9e) [160]. It was found that MOF-818 exhibited both SOD- and CAT-like activities. The prepared hydrogel exhibited significant thermosensitivity, maintaining a fluidic state at 4 °C and undergoing rapid gelation at physiological temperature. This property enabled its prolonged retention at wound sites. Emulating natural antioxidant enzymes, Liu et al. engineered Ni-HHTP MOF nanozyme with SOD- and GPx-like activities to promote wound healing [161]. It was found that the nanozyme not only can eliminate ·OH and H_2_O_2_ but also can promote angiogenesis by the released Ni^2+^ ions. Further investigations revealed that the MOF nanozyme promoted healing of diabetic wounds by the combination of three coordinated mechanisms: mitigation of oxidative stress, suppression of inflammatory cascades, and potentiation of neovascularization in diabetic wound tissues (Fig. 9f).

**Fig. 10:**
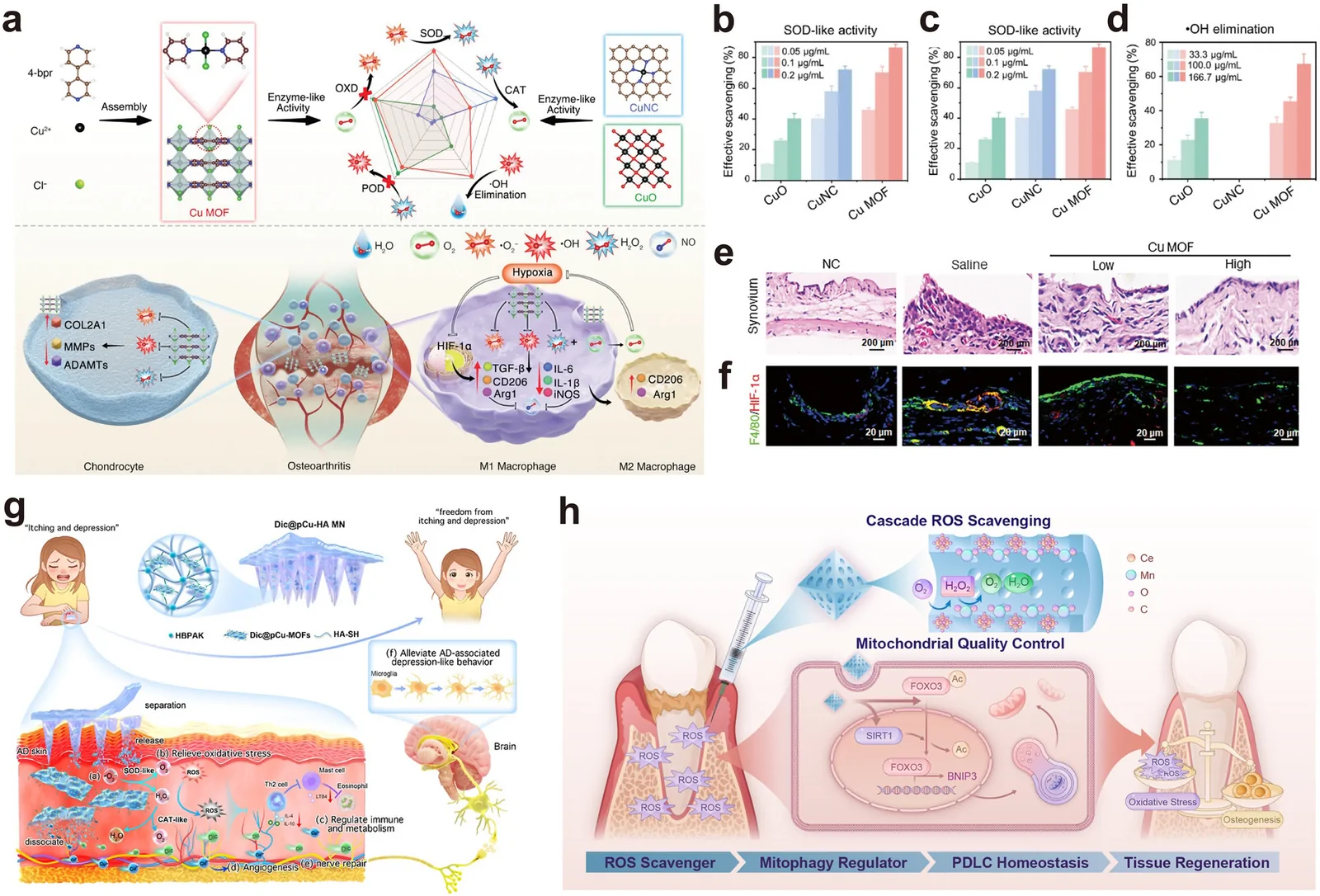
Applications of MOF nanozymes for inflammatory disease therapy. **a** Synthesis method of MOF nanozyme and the mechanism for OA treatment. **b** SOD-like activity of Cu-MOF. **c** CAT-like activity of Cu-MOF. **d** Elimination capacity of Cu-MOF against ·OH. **e** H&E staining images of knee joint synovium in osteoarthritis mice after treatment. **f** Immunofluorescence staining images of knee joint synovium in osteoarthritis mice after treatment. Figures **a** to **f** are reproduced with permission [164]. Copyright 2024, Wiley–VCH. **g** Mechanism illustration of Dic@pCu-HA MN for atopic dermatitis treatment. Reproduced with permission [199]. Copyright 2025, American Chemical Society. **h** Schematic of MnO_2_@UiO-66(Ce) nanozyme to alleviate alveolar bone resorption in periodontitis. Reproduced with permission [165]. Copyright 2025, Elsevier

MOF nanozymes also demonstrate significant application potential in the treatment of inflammatory diseases, primarily through two core mechanisms. First, they leverage the multi-enzyme activities to scavenge excessive ROS at inflammatory sites. Second, they exert immunomodulatory functions by driving macrophage polarization from the pro-inflammatory M1 phenotype toward the anti-inflammatory M2 phenotype [162, 163]. Yu et al. synthesized Cu-based MOF nanozyme for treatment of osteoarthritis (OA) [164]. The nanozyme exhibited powerful SOD- and CAT-like activities, and its efficient oxygen-generating capacity alleviated hypoxia in OA microenvironments, which further facilitated macrophage repolarization from M1 to M2 phenotypes (Fig. 10a). The enzyme-like activities and ROS removal capacity of Cu-MOF were validated in vitro (Fig. 10b-d). In addition, in vivo OA therapeutic efficiency of the nanozyme was assessed in collagenase-induced OA mice. The H&E staining images indicated that after nanozyme treatment, synovial hyperplasia was attenuated from pathological thickening (7–8 layers) to near-physiological levels (2–3 layers) (Fig. 10e). Immunofluorescence staining images further confirmed that the treatment can reduce the expression of HIF-1α in macrophages (Fig. 10f). Apart from acting as ROS scavenger, MOF nanozyme can also load anti-inflammatory agents by virtue of its pore structures, thereby increasing the therapeutic efficacy. Recently, Liu et al. developed Cu-MOF nanozyme-loaded microneedle patch for atopic dermatitis treatment. The MOF nanozyme exhibited dual functionality as cargo to load dictamnine and ROS scavenger by multiple enzyme-mimicking activities (Fig. 10g). ROS-scavenging capacity of the microneedle patch was validated in both HaCaT cells and RAW264.7 macrophages. In addition, iNOS immunofluorescent staining confirmed the potent anti-inflammatory properties of the microneedle patch. Similarly, Zhu et al. synthesized MnO_2_@UiO-66(Ce) nanozyme for periodontitis treatment [165]. MnO_2_ was introduced within UiO-66 MOFs via coupling with the Ce clusters (Fig. 10h). The synergistic antioxidant intervention restored mitochondrial dynamics and osteogenic functions in periodontal ligament cells. In addition, it also attenuated inflammation-driven alveolar bone resorption in ligature-induced periodontitis model mice.

### Antibacterial Therapy

Bacterial infections represent predominant drivers of mortality in burn-injured patients, with antimicrobial agents constituting the main therapeutic intervention in contemporary clinical practice [166]. However, the pervasive employment of antibiotics has caused the emergence of multidrug-resistant bacteria and compromised therapeutic efficacy [167]. Encouragingly, MOF nanozymes have shown great potential for antibacterial therapy in recent years. They exert antibacterial effects primarily via the following mechanisms: (1) Mimicking natural enzymes (e.g., POD) to catalyze the generation of highly toxic free radicals, which directly oxidize and damage the bacterial cell membrane; (2) Releasing metal ions (e.g., Ag⁺, Cu^2+^, Zn^2+^) from the MOF structure or loaded metal NPs, disrupting bacterial membrane integrity or interfering with their metabolism; (3) Utilizing the GPx-like activity to consume the GSH within bacteria, thereby weakening their antioxidant defense capacity and increasing their susceptibility to ROS; (4) Taking advantage of the intense heat generated under near-infrared light irradiation to denature bacterial membrane proteins, thereby directly killing the bacteria [168, 169]. Compared to traditional antibiotic drugs, MOF nanozymes offer several advantages in antibacterial applications. The most significant one is that they hardly induce bacterial resistance. This is because MOF nanozymes can exert synergistic effects through multiple mechanisms and cause physical disruption of bacterial structures, providing a novel solution to the global challenge of antibiotic resistance [170].

**Fig. 11:**
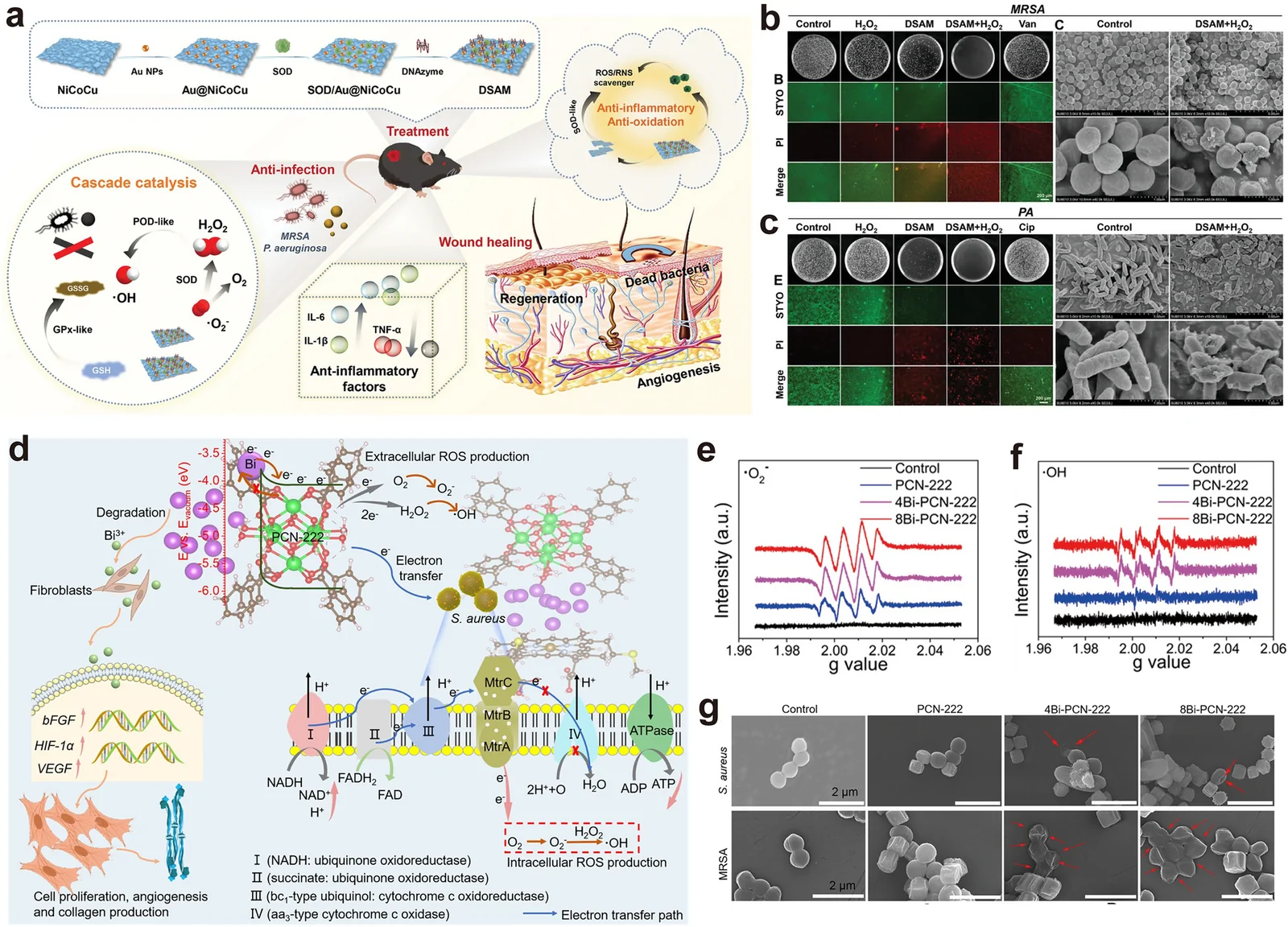
Applications of MOF nanozymes for antibacterial therapy. **a** Synthesis method of multi-enzyme-integrated nanoplatform and the mechanism of treatment of bacteria-infected wounds. **b** Antibacterial effects, live/dead double-staining images, and corresponding SEM images of MRSA after different treatments. **c** Antibacterial effects, live/dead double-staining images, and corresponding SEM images of PA after different treatments. Figures a to c are reproduced with permission [172]. Copyright 2023, Wiley–VCH. **d** Mechanism illustration of the antibacterial effect and promotion of wound healing. **e** Detection of ·O_2_^−^ generated by Bi-PCN-222 via ESR. **f** Detection of ·OH generated by Bi-PCN-222 via ESR. **g** FESEM pictures of *S. aureus* and MRSA after different treatments. Figures **d** to **g** are reproduced with permission [173]. Copyright 2023, American Chemical Society

For instance, Zhang et al. prepared thermosensitive hydrogel (Ce6@MOF-Gel) for accelerated healing of infected wounds [171]. They evaluated the potential risk of drug resistance by comparing the time before the logarithmic phase of bacteria after treatment of Ce6@MOF-Gel and penicillin, respectively. It was found that the time before logarithmic growth remained stable in the group of Ce6@MOF-Gel even after six passages, while the time needed to reach the logarithmic growth phase in the penicillin group was significantly reduced after three passages. These results demonstrate that Ce6@MOF-Gel treatment is not prone to induce bacterial resistance. Mo et al. synthesized NiCoCu-MOF nanozyme for enhanced treatment of bacteria-infected wounds [172]. The nanozyme was synthesized via one-pot method at RT, followed by the loading of Au NPs, natural SOD, and DNAzyme (Fig. 11a). After implanting at the site of infected wounds, SOD can convert ·O_2_^−^ into O_2_ and H_2_O_2_, and the generated H_2_O_2_ is subsequently decomposed by Au NPs and DNAzyme, leading to the release of ·OH and bacterial death. In addition, due to the GPx-like activity of released Cu^2+^, the nanoplatform reinforces antibacterial efficacy through concurrent GSH depletion and ·OH preservation, amplifying oxidative stress-mediated bacterial eradication. The potent antibacterial capacity of nanozyme was demonstrated by several different means, including colony formation, live/dead staining, and SEM observation (Fig. 11b, c). Electron transfer is fundamental to bacterial bioenergetics, enabling membrane–respiratory processes which are essential for cellular proliferation. Disruption of the electron transport chain can induce intracellular ROS overproduction by impairing redox homeostasis, thereby hindering bacterial growth. Based on this, Wu et al. developed bismuth (Bi) NPs-doped PCN-222 MOF nanozyme for treatment of bacteria-infected wounds [173]. Bi NPs functioned as electron donors, enabling spontaneous electron migration to both the PCN-222 matrix and bacteria, thereby ​elevating intracellular ROS levels and enhancing antibacterial efficacy (Fig. 11d). In addition, the nanozyme can effectively catalyze O_2_ and H_2_O_2_ to further increase the generation of ROS (Fig. 11e, f). The prepared nanozyme achieved efficient elimination of both *S. aureus* and MRSA (Fig. 11g).

### Biosensing

Leveraging advantages such as tunable structure and catalytic activity, MOF nanozymes have been widely applied in biosensing recently (Table 4). The basic working principle is that they can induce changes in specific parameters of the detection system (e.g., color, UV–Vis absorption, fluorescence signals, and electrochemical signals) in the presence of target analytes, which exhibit a quantitative relationship with the concentration of target substances [174, 175]. By detecting these signals with specialized instruments, quantitative analysis of the target substances can be realized. MOF nanozymes with OXD- and POD-like activities catalyze electron transfer processes that oxidize chromogenic indicators (e.g., TMB, OPD, ABTS), resulting in the production of visible color changes [176]. Colorimetric methods offer significant practical advantages, including rapid response, cost-effectiveness, and relatively convenient visual detection, making them ideal for biosensing applications [177]. Fluorescence-based biosensing platforms stand out for rapid response kinetics, exceptional sensitivity, and multi-parametric resolution capabilities [178]. In addition, MOF nanozymes can also be utilized in electrochemical detection. They combine catalytic reactions with electrochemical signal conversion, offering ultra-high sensitivity and specificity, which enables the direct detection of trace disease markers and pathogens in complex samples.

**Table 4:** Summary of MOF nanozymes for biosensing applications

Metal ions/ligands	Synthesis method	Enzyme-like activities	Detection mode	Targets	Linear Range	LOD	References
Zr_6_ cluster, H_3_BTC	Solvothermal method	Phosphatase	Colorimetric method	OPs	0.6–300 μM	0.18 μM	[39]
Fe^3+^, NH_2_-BDC	Solvothermal method	POD	Colorimetric method	Arsenic	5–600 μg L^−1^	2.78 μg L^−1^	[216]
Zr^4+^, Fe^3+^, H_2_BDC-NH₂, Pt NPs	Solvothermal method, in situ growth	POD	Colorimetric method	Aflatoxins	0.0963–0.3971 mg L^−1^	0.0636 μg L^−1^	[185]
Zn^2+^, Co^2+^, Fe^3+^, 2-methylimidazole, H_2_BDC	Solvothermal method	POD	Colorimetric method	Glyphosate	0.02–40 mg L^−1^	1 μg L^−1^	[207]
Zn^2+^, Ir^3+^, NDC	Solvothermal method	OXD/POD	Colorimetric method	Glucose	2.66–319 μM	1.9 μM	[208]
Zr^4+^, Ru^2+^, BDC-NH_2_, Pt NPs	Solvothermal method, sonication	POD	Colorimetric and electrochemiluminescence method	Monkeypox virus	60–3 × 10^11^ copies μL^−1^	0.1 pM and 10 aM	[182]
Fe^3+^, H_2_TDC	Solvothermal method	POD	Colorimetric method	Pyrophosphate	0.10–2.0 μM	0.03 μM	[209]
Fe^3+^, Ce^3+^, Tb^3+^, BBDC	Solvothermal method	POD	Fluorescent method	Caffeic acid	50–500 μM	18.9 nM	[18]
Zr^4+^, H_2_FDC	Solvothermal method	Phosphatase	Fluorescent method	Ascorbic acid	0.08–11 mg L^−1^	0.025 mg L^−1^	[183]
Ag^+^, PMA, 4,4’-bipyridine	Solvothermal method	POD	Electrochemical method	Superoxide and nitric oxide	1–1000 μM and 1–850 μM	0.27 and 0.34 nM	[217]
Fe^2+^, BDC	Ultrasonic method	OXD	Colorimetric method	Methidathion	0.01–1.4 mM	5.48 nM	[218]
Zr^4+^, TCPE	Solvothermal method, ultrasonication	Phosphatase	Colorimetric/fluorescence approach	Paraoxon	1.82–181.69 μM	0.178 μM	[184]
Mn^2+^, triethanolamine, SiO_2_	Chemical sedimentation	Phosphatase	Colorimetric method	Glyphosate	0.49–750 μM	0.61 μM	[219]
Mn^2+^, glutaric acid	Solvothermal method	OXD	Colorimetric method	Nitrite ions	5–45 μM	0.15 μM	[220]
Cu^2+^, 2-Mim	Solvothermal method	Laccase	Colorimetric method	Phenolic compounds	0.1–100 μM	0.068 μM	[221]

Compared with biosensing platforms constructed by other materials, MOF nanozymes offer the following key advantages. First, owing to the synergistic effects between porous structures and active sites, they exhibit broader linear ranges. Additionally, high-density active site distribution and enhanced electron transfer efficiency significantly lower their limit of detection (LOD) [179]. Second, structural tunability facilitates performance optimization in MOF nanozyme-based sensing platforms [180]. Adjusting metal ions and ligands can enhance catalytic activity and selectivity toward target substances. Furthermore, the mesoporous architectures promote substrate mass transfer, while surface functional groups (e.g., amino, carboxyl) are introduced to mitigate interference from sample impurities. Third, they demonstrate superior stability and reusability. MOF nanozyme-based biosensing platforms retain activity across a broad pH and temperature range, with minimal activity loss upon repeated use [181]. Nevertheless, biosensing platforms based on MOF nanozymes also have their own limitations. First, the catalytic efficiency of nanozymes is generally inferior to that of natural enzymes, and they often lack sufficient selectivity. Second, the mesoporous structure of MOF nanozymes may lead to mass transfer resistance, potentially resulting in longer response times.

**Fig. 12:**
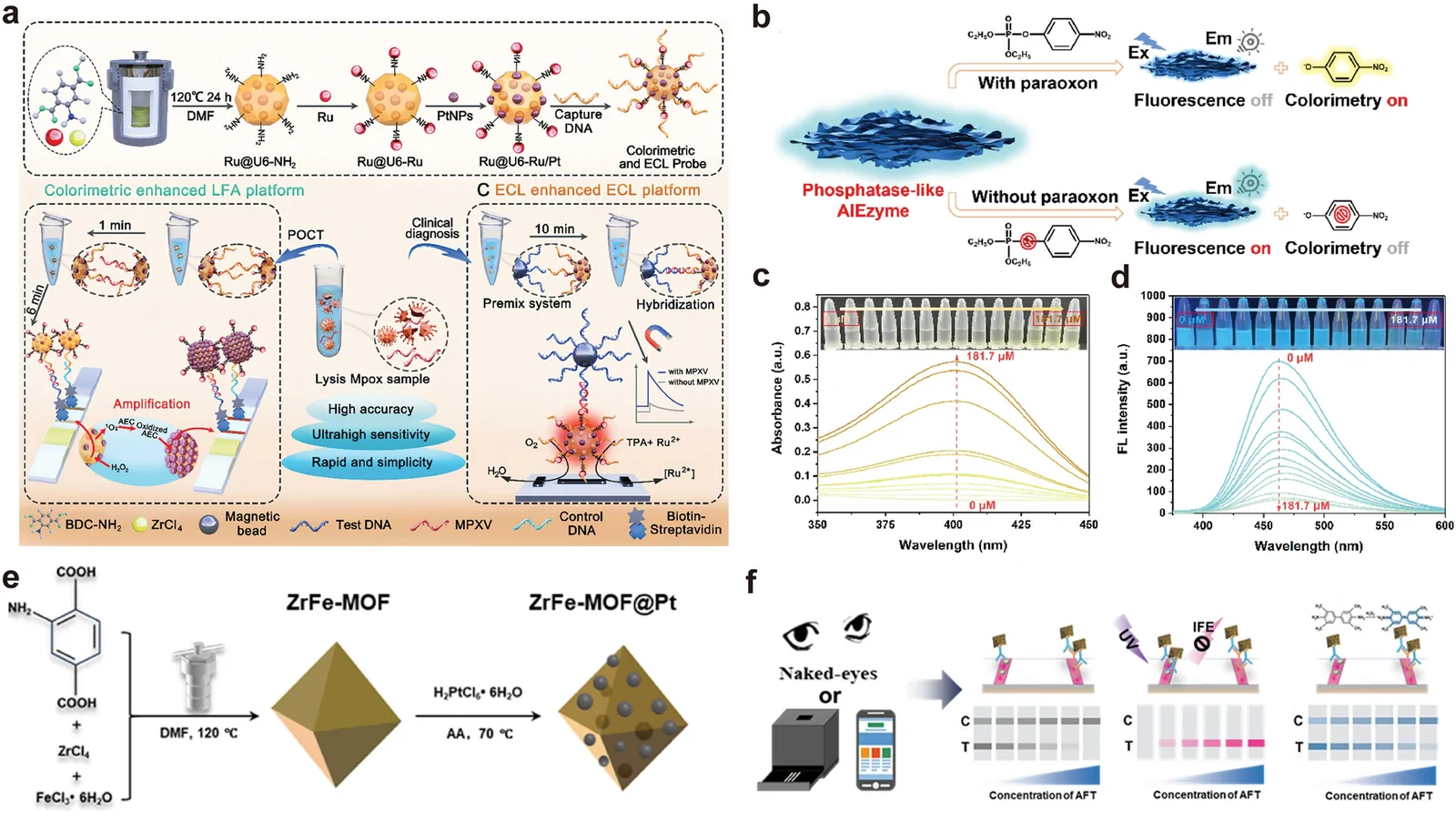
Applications of MOF nanozymes for biosensing. **a** Synthesis process of Ru@U6-Ru/Pt sensing platform and the mechanism of monkeypox virus detection. Reproduced with permission [182]. Copyright 2024, Wiley–VCH. **b** Schematic illustration of the bimodal detection mechanism of paraoxon. **c** UV–Vis absorption curves of AIEzyme with paraoxon at different concentrations. **d** Fluorescence spectra of AIEzyme with paraoxon at different concentrations. Figures **b** to **d** are reproduced with permission [184]. Copyright 2025, Elsevier. **e** Synthesis process of the ZrFe-MOF@Pt platform. **f** Mechanism illustration of aflatoxin detection based on the MOF nanozyme sensing platform. Figures e and f are reproduced with permission [185]. Copyright 2025, Elsevier

Yang et al. developed Zr-MOF-based colorimetric and electrochemiluminescence (ECL) dual-mode biosensing platform for diagnosis of Mpox [182]. The biosensing platform was constructed by integrating double-layered ECL luminophores and Pt NPs using Zr-MOF as the carrier (Fig. 12a). The colorimetric mode realized the screening of monkeypox virus with a LOD of 0.1 pM and the ECL mode achieved quantitative detection of monkeypox virus with a LOD of 10 aM. The sensitivity and accuracy of the sensing platform were validated with detection of 50 clinical samples, which was 100% concordant to the results of quantitative polymerase chain reaction (qPCR). Wu and coworkers designed and prepared a MOF nanozyme-based fluorescent sensing platform for ascorbic acid (AA) detection [183]. The Zr-CAU-28 MOF synthesized via one-pot solvothermal method demonstrated potent phosphatase-mimicking capability, catalyzing 4-methylumbelliferyl phosphate (4-MUP) dephosphorylation to yield fluorescent 4-methylumbelliferone (4-MU). AA competitively adsorbs onto zirconium oxo-clusters via multidentate hydrogen-bonding interactions, thereby occupying catalytic sites and attenuating the fluorescence intensity. This inhibition mechanism enabled selective AA quantification with a wide linear range (0.08–11 μg mL⁻^1^) and low LOD (0.025 μg mL⁻^1^), circumventing interference from coexisting reductants in complex matrices.

Nanozyme-based organophosphorus pesticides (OPs) sensing platforms predominantly rely on chromogenic substrate oxidation to generate target-dependent color signals, yet they face dual constraints: First is susceptibility to redox interferents ubiquitously present in environmental matrices, and second is inherent error risks from single-signal readouts that restrict application scope. To mitigate the limitations, Zhu et al. developed a Zr-based MOF sensing platform integrating phosphatase-like activity and aggregation induced emission (AIE) fluorescence for facile and rapid detection of paraoxon [184]. The MOF nanozyme was prepared by solvothermal method, with Zr^4+^ and TCPE functioning as metal nodes and AIE ligands (Fig. 12b). The phosphatase-like activity can hydrolyze P-O bond in paraoxon, releasing the colored* p*-nitrophenol. When no paraoxon is in the system, the fluorescence is “on” and no *p*-nitrophenol is generated. With the increase in paraoxon concentration, the UV–Vis absorbance intensity at 402 nm increased accordingly (Fig. 12c). In contrast, fluorescence quenching intensified proportionally with elevated paraoxon concentrations, demonstrating a concentration-dependent attenuation profile (Fig. 12d). Recently, Sun et al. developed a triple-signal MOF biosensing platform for sensitive aflatoxins detection [185]. ZrFe-MOF was first synthesized via hydrothermal method, and platinum metal precursors were subsequently reduced on the MOF to prepare ZrFe-MOF@PtNPs nanozyme (Fig. 12e). By further modifying with aflatoxin antibodies, the prepared immunoprobe achieved highly sensitive detection of aflatoxin through triple-signal co-output (Fig. 12f).

Very recently, You et al. reported a TCPP(Fe)-Zn-based sensing platform for electrochemical and colorimetric detection of sarcosine (Sar) [186]. The sensor was fabricated using a nanocomposite consisting of riboflavin (Rf) and reduced graphene oxide nanoribbons (rGONRs), which served as a support for the MOF nanozyme. The rGONRs significantly increased the active surface area and facilitated electron transfer, thereby enhancing the POD-like activity of the TCPP(Fe)-Zn MOF. This catalytic activity promoted the oxidation of o-phenylenediamine (OPD) in the presence of H_2_O_2_ generated via an Sar-specific oxidase-mimetic reaction mediated by Rf. The sensor exhibited a broad linear range of 10.0–1000.0 μmol L^−1^ for colorimetric detection, and 0.001–500.0 μmol L^−1^ for electrochemical detection, achieving detection limits of 2.0 μmol L^−1^ and 0.04 nmol L^−1^, respectively.

### Biosafety Assessment of MOF Nanozymes

Before applying MOF nanozymes in living organisms, it is crucial to conduct a comprehensive assessment of their biosafety. The biosafety of MOF nanozymes depends not only on their components (e.g., metal ions, organic ligands), but also on their size, morphology, surface charge, aggregation state, and intrinsic catalytic activities [187]. Therefore, the biosafety evaluation constitutes a complex and multi-parameter system. In this section, we focus primarily on two key aspects of MOF nanozymes: First is the metabolic pathway within the body, and second is the potential immune response they may cause.

After intravenous injection, MOF nanozymes distribute throughout the body via the bloodstream and accumulate in organs or tissues rich in the mononuclear phagocyte system, such as the liver and spleen. Additionally, due to the enhanced permeability and retention (EPR) effect, they tend to accumulate in solid tumors [188]. Currently, the primary method for tracing the in vivo distribution of MOF nanozymes relies on fluorescent labeling techniques. Though this method provides valuable information on the distribution process, its accuracy can be affected by factors such as the detachment of fluorescent labels and the quenching of fluorescence signals in the complex physiological environment. Therefore, accurately assessing the distribution of MOF nanozymes in living organisms remains a major challenge. Upon completion of experimental observations, detecting metal ion concentrations in tissues or organs using methods such as the inductively coupled plasma optical emission spectrometer (ICP-OES) represents a more reliable approach for evaluating the distribution and accumulation of MOF nanozymes. Furthermore, MOF nanozymes enter cells primarily through endocytosis pathways mediated by clathrin and caveolin. Within the acidic environment of cellular lysosomes and under the action of enzymes, the MOF framework gradually disassembles, releasing metal ions and organic ligands, which are ultimately excreted from the body via the kidneys or the hepatobiliary system. Although numerous studies have reported that MOF nanozymes exhibit a favorable biosafety profile, after nearly one decade, the potential long-term accumulation toxicity of metal ions deserves greater attention and further research.

The interaction between MOF nanozyme and the immune system is a double-edged sword. While they can modulate immune responses for therapeutic purposes, they may also trigger unintended immune activation or suppression. Therefore, close attention should be paid to the potential immune reactions elicited by MOF nanozymes in the body. They can mediate immunostimulatory effects through interactions with the complement system, acting as haptens that bind to endogenous proteins, or by inducing inflammatory responses [189]. The pyrogenicity of MOF NPs, for instance due to endotoxin contamination, can lead to fever and severe organ damage via immune activation. Conversely, the cytotoxicity on immune cells may result in immunosuppression. The biosafety assessments of MOF nanozymes primarily include evaluations of hemocompatibility, immunocytotoxicity, and acute and chronic toxicity tests. Currently, surface modification strategies, such as PEGylation and liposome encapsulation, are often employed to reduce the immunogenicity of MOF nanozymes [190].

## Summary and Outlook

MOF nanozymes integrating designable and tunable structures with multiple enzyme-like activities have demonstrated tremendous application prospects in the biomedical field. In this review, we first focused on the rational design of MOF nanozymes, with particular emphasis on the impact of metal ions, organic ligands, and morphology on their enzymatic activity, providing guidance for their tailored development. Subsequently, we summarized the primary synthesis methods for MOF nanozymes and discussed the advantages and limitations associated with different synthesis strategies. Furthermore, we systematically outlined the enzyme-like activities exhibited by MOF nanozymes, paying special attention to ROS-generating activities (e.g., OXD- and POD-like) and ROS-scavenging activities (e.g., SOD- and CAT-like), as well as phosphatase-like activity. Finally, through specific case studies, we highlighted the applications of MOF nanozymes in the biomedical field, including tumor therapy, management of oxidative stress and inflammatory diseases, antibacterial therapy, and biosensing. A key focus is placed on elucidating the mechanisms by which MOF nanozymes leverage their enzyme-like activities to exert biological effects. After nearly a decade of intensive investigation, substantial progress has been made in the development of MOF nanozymes, particularly in elucidating catalytic mechanisms, optimizing enzyme-like activities, and expanding biomedical applications. Nevertheless, MOF nanozymes still face the following challenges in both fundamental research and clinical translation.

(1) Scalability and reproducibility of MOF nanozymes. As summarized in Sect. 3, there are currently various methods for preparing MOF nanozymes. However, most of these methods are suitable for small-scale production in laboratory settings and face significant challenges when applied to industrial-scale manufacturing. Key issues include non-uniform mass and heat transfer, as well as the inability to effectively control product morphology. While microfluidic synthesis and solvent-free mechanochemical preparation show high potential for scale-up, they also possess their own limitations. Therefore, finding a balance between large-scale production and a suitable preparation method is a critical consideration. In addition, more reliable synthesis strategies for MOF nanozymes should be developed, for instance, by transitioning from batch to continuous preparation schemes and refining heating methods.

(2) The correlations between structure and properties of MOF nanozymes remain to be further elucidated. Although MOF nanozymes with diverse natural enzyme-like activities have been developed, ​the underlying structure–property relationships are still inadequately elucidated. Current studies predominantly follow a reverse research paradigm: synthesizing MOF materials first and subsequently screening their enzyme-like activities. Deciphering these correlations would enable rational forward-design of MOF nanozymes. With the rapid advancement of AI technologies, deciphering the 3D structures of MOF nanozymes and predicting their enzyme-mimicking properties will become increasingly feasible and efficient.

(3) The enzymatic activity and specificity of MOF nanozymes still cannot match those of natural enzymes. As summarized in Sect. 4, although a variety of MOF nanozymes with diverse enzyme-like activities have been developed, the catalytic performance of most nanozymes remains inferior to that of natural enzymes. Consequently, this determines that nanozymes cannot replace natural enzymes in exerting biological effects in the short term. Furthermore, the specificity of MOF nanozymes is insufficient. Some of them simultaneously possess multiple enzyme-like activities, such as exhibiting both CAT- and POD-like activities. When applied in antioxidant stress therapy, while they can scavenge free radicals, they may also generate ·OH at the same time, which may compromise the therapeutic efficacy. Therefore, in the design stage of MOF nanozymes, fully considering the enhancement of their catalytic activity and specificity represents a crucial direction for improving the practical utility of MOF nanozymes.

(4) Clinical translation hurdles. Despite the numerous advantages of MOF nanozymes and their encouraging therapeutic outcomes in preclinical studies, the pace of their clinical translation remains slow. To date, no MOF nanozyme drug has been officially approved for clinical use. Several key challenges are hindering this transition. First is the potential cytotoxicity of metal ions. Although many cellular and animal level studies suggest that MOF materials have favorable biosafety profile, the metal ions released within the body post-administration may exhibit unpredictable toxicity profiles. Furthermore, the vast variety of metal ions and organic ligands make it extremely difficult to make a comprehensive safety assessment of MOF nanozymes. Second is the risk of in vivo degradation. The complex physiological environment poses a risk of degradation for MOF nanozymes, which further heightens safety concerns. Moreover, research on the metabolic pathways and pharmacokinetics of MOF nanozymes within the body is still not thorough. In conclusion, safety concerns represent the primary obstacle preventing the clinical translation of MOF nanozymes.

To promote the further development of MOF nanozymes in the biomedical field, we propose the following research directions with transformative potential.

(1) The development of MOF nanozyme-based intelligent robots. Although some stimuli-responsive MOF nanoplatforms have been reported, which enable controlled drug release triggered by internal or external stimuli, their spatiotemporal precision and adaptive intelligence require further enhancement. To address this issue, in vivo self-assembling MOF nanozymes can be engineered to augment targeting accuracy through TME-activated structural reorganization. Concurrently, AI-aided MOF nanozyme robot design may optimize the catalytic activity and improve therapeutic efficacy. Furthermore, theranostic nanorobots integrating real-time imaging and on-demand therapy represent a transformative frontier for precision medicine.

(2) The development of MOF nanozyme-based commercial and portable detection devices. Although numerous MOF nanozyme-based biosensing platforms have been reported, most remain confined to the laboratory validation phase, with significant gaps to bridge before achieving commercial-scale manufacturing and daily use by ordinary people. We advocate for accelerating the transition of these validated sensing platforms toward commercial detection devices or diagnostic kits. Such advancement will not only broaden accessibility to cutting-edge detection technologies but also catalyze widespread adoption and sustained evolution of MOF nanozymes in practical settings.

(3) Standardizing evaluation criteria for catalytic activity of MOF nanozymes. Currently, a large number of MOF nanozymes have been extensively studied and reported. However, there is a notable lack of unified evaluation criteria​ for their catalytic activity. Therefore, it is imperative to establish standardized evaluation criteria that encompass key parameters such as substrate selection, reactant concentration, temperature, and other relevant variables. Implementing such standards would significantly enhance the comparability of data across different studies, drive the rational design and optimization of MOF nanozymes, and ultimately accelerate their clinical translation.

(4) The development of AI-driven full-industry chain for MOF nanozymes from design to synthesis. While AI technology currently provides valuable guidance in the design of MOF nanozymes, its potential in the full industrial chain remains vastly underutilized. We anticipate that AI technology will find broader application in the synthesis of MOF nanozymes, with the introduction of AI-powered robotic platforms to enhance production efficiency. It moves beyond traditional trial-and-error methods, enabling the de novo design of MOF nanozymes with customized functions. This integrated “design-to-production” pipeline would significantly accelerate MOF nanozyme development, reduce costs, and enhance reproducibility, paving the way for precision nanomedicine.

In summary, although some challenges persist in MOF nanozyme research, we foresee that converging advances in nanomedicine, materials science, biology, and AI technology will systematically overcome these barriers. MOF nanozymes will present greater value and better contributions to human health in the near future.

## References

[1] S.J. Benkovic, S. Hammes-Schiffer, A perspective on enzyme catalysis. Science **301**(5637), 1196–1202 (2003). 10.1126/science.1085515 12947189 10.1126/science.1085515

[2] M. Liang, X. Yan, Nanozymes: from new concepts, mechanisms, and standards to applications. Acc. Chem. Res. **52**(8), 2190–2200 (2019). 10.1021/acs.accounts.9b00140 31276379 10.1021/acs.accounts.9b00140

[3] L. Gao, J. Zhuang, L. Nie, J. Zhang, Y. Zhang et al., Intrinsic peroxidase-like activity of ferromagnetic nanoparticles. Nat. Nanotechnol. **2**(9), 577–583 (2007). 10.1038/nnano.2007.260 18654371 10.1038/nnano.2007.260

[4] J. Wu, X. Wang, Q. Wang, Z. Lou, S. Li et al., Nanomaterials with enzyme-like characteristics (nanozymes): next-generation artificial enzymes (II). Chem. Soc. Rev. **48**(4), 1004–1076 (2019). 10.1039/c8cs00457a 30534770 10.1039/c8cs00457a

[5] M. Tang, J. Ni, Z. Yue, T. Sun, C. Chen et al., Polyoxometalate-nanozyme-integrated nanomotors (POMotors) for self-propulsion-promoted synergistic photothermal-catalytic tumor therapy. Angew. Chem. Int. Ed. **63**(6), e202315031 (2024). 10.1002/anie.202315031 10.1002/anie.202315031 38117015

[6] H. Gu, J. Li, P. Dai, T. Sun, C. Chen et al., Polyphenol oxidase-like nanozymes. Adv. Mater. **37**(43), e09346 (2025). 10.1002/adma.202509346 40847695 10.1002/adma.202509346

[7] Q. Wu, H. Zhou, B. Xu, H. Yang, Y. Wang et al., A hollow nucleobase coordinated nanozyme for tumor catalytic therapy. Small Methods **10**(3), e2500931 (2026). 10.1002/smtd.202500931 40685939 10.1002/smtd.202500931

[8] H. Wei, E. Wang, Nanomaterials with enzyme-like characteristics (nanozymes): next-generation artificial enzymes. Chem. Soc. Rev. **42**(14), 6060–6093 (2013). 10.1039/c3cs35486 23740388 10.1039/c3cs35486e

[9] X. Huang, S. Zhang, Y. Tang, X. Zhang, Y. Bai et al., Advances in metal–organic framework-based nanozymes and their applications. Coord. Chem. Rev. **449**, 214216 (2021). 10.1016/j.ccr.2021.214216

[10] C. Zhang, Y. Wu, D. Li, H.-L. Jiang, Recent advances in MOF composites for photocatalysis. Chem. Sci. **16**(29), 13149–13172 (2025). 10.1039/d5sc03065j 40620342 10.1039/d5sc03065j PMC12227101

[11] T. Wang, J. Yang, J. Ding, Y. Yuan, Y. Wu et al., A metal-organic framework-based immune-regulating nanocarrier depot for enhanced combination cancer immunotherapy. ACS Nano **19**(26), 23629–23646 (2025). 10.1021/acsnano.5c01678 40552914 10.1021/acsnano.5c01678

[12] N. Feng, Y. Wei, P. Li, Y. Zheng, X. Li et al., Dual-signal amplified self-powered biosensor integrating DNAzyme Walker and Dumbbell *HCR* for attomolar detection of TATA-28 DNA in thalassemia gene screening. Biosens. Bioelectron. **287**, 117683 (2025). 10.1016/j.bios.2025.117683 40516431 10.1016/j.bios.2025.117683

[13] Y. Sun, S.-L. Ding, X. Zhao, D. Sun, Y. Yang et al., Self-reinforced MOF-based nanogel alleviates osteoarthritis by long-acting drug release. Adv. Mater. **36**(39), e2401094 (2024). 10.1002/adma.202401094 38684182 10.1002/adma.202401094

[14] D. Feng, Z.-Y. Gu, J.-R. Li, H.-L. Jiang, Z. Wei et al., Zirconium-metalloporphyrin PCN-222: mesoporous metal-organic frameworks with ultrahigh stability as biomimetic catalysts. Angew. Chem. Int. Ed. **51**(41), 10307–10310 (2012). 10.1002/anie.201204475 10.1002/anie.201204475 22907870

[15] J. Liu, Y. Wang, M. He, Y. Gao, Q. Pan et al., Cerium-based metal–organic framework nanozyme with high oxidase-like activity at neutral pH for discrimination and detection of antioxidants. Biosens. Bioelectron. **285**, 117608 (2025). 10.1016/j.bios.2025.117608 40419415 10.1016/j.bios.2025.117608

[16] Z. Chi, J. Gu, H. Li, Q. Wang, Recent progress of metal-organic framework-based nanozymes with oxidoreductase-like activity. Analyst **149**(5), 1416–1435 (2024). 10.1039/d3an01995k 38334683 10.1039/d3an01995k

[17] Y. Liu, S. Sun, C. Shang, R. Liu, C. Zhang et al., MOF-derived nanozymes loaded with botanicals as multifunctional nanoantibiotics for synergistic treatment of intracellular antibiotic-resistant bacterial infection. Nanoscale Horiz. **10**(7), 1377–1389 (2025). 10.1039/d5nh00137d 40327007 10.1039/d5nh00137d

[18] X. Liao, B. Li, L. Wang, Y. Chen, Boric acid functionalized Fe_3_O_4_@CeO_2_/Tb-MOF as a luminescent nanozyme for fluorescence detection and degradation of caffeic acid. Biosens. Bioelectron. **264**, 116637 (2024). 10.1016/j.bios.2024.116637 39146768 10.1016/j.bios.2024.116637

[19] J. Shan, L. Du, X. Wang, S. Zhang, Y. Li et al., Ultrasound trigger Ce-based MOF nanoenzyme for efficient thrombolytic therapy. Adv. Sci. **11**(20), 2304441 (2024). 10.1002/advs.202304441 10.1002/advs.202304441 PMC11132072 38576170

[20] Y. Liu, Y. Cheng, H. Zhang, M. Zhou, Y. Yu et al., Integrated cascade nanozyme catalyzes *in vivo* ROS scavenging for anti-inflammatory therapy. Sci. Adv. **6**(29), eabb2695 (2020). 10.1126/sciadv.abb2695 10.1126/sciadv.abb2695 PMC7439611 32832640

[21] Y. Cai, Y. Wu, Y. Tang, W. Xu, Y. Chen et al., *In situ* defect engineering of Fe-MIL for self-enhanced peroxidase-like activity. Small **20**(46), 2403354 (2024). 10.1002/smll.202403354 10.1002/smll.202403354 39101616

[22] Z. Feng, G. Chen, M. Zhong, L. Lin, Z. Mai et al., An acid-responsive MOF nanomedicine for augmented anti-tumor immunotherapy *via* a metal ion interference-mediated pyroptotic pathway. Biomaterials **302**, 122333 (2023). 10.1016/j.biomaterials.2023.122333 37738743 10.1016/j.biomaterials.2023.122333

[23] J. Bao, J. Wang, S. Chen, S. Liu, Z. Wang et al., Coordination self-assembled AuTPyP-Cu metal–organic framework nanosheets with pH/ultrasound dual-responsiveness for synergistically triggering cuproptosis-augmented chemotherapy. ACS Nano **18**(12), 9100–9113 (2024). 10.1021/acsnano.3c13225 38478044 10.1021/acsnano.3c13225

[24] J. Sun, Z. Zhao, X. Wei, J. Yang, D. Li et al., Multi-bioactive poly(amino acid)-metal-organic framework nanocomposite for reinforced cascading photodynamic immunotherapy of cancer. Biomaterials **324**, 123488 (2026). 10.1016/j.biomaterials.2025.123488 40554219 10.1016/j.biomaterials.2025.123488

[25] R. Wang, Z. Yue, W. Feng, Y. Sun, X. Hai et al., Hybridization of polyoxometalates and metal-organic frameworks for effective tumor chemodynamic therapy and sonodynamic therapy. Nano Mater. Sci. **8**(1), 244–253 (2026). 10.1016/j.nanoms.2024.10.015

[26] Z. Shen, D. Xu, G. Wang, L. Geng, R. Xu et al., Novel colorimetric aptasensor based on MOF-derived materials and its applications for organophosphorus pesticides determination. J. Hazard. Mater. **440**, 129707 (2022). 10.1016/j.jhazmat.2022.129707 35986944 10.1016/j.jhazmat.2022.129707

[27] H. Zhao, C. Serre, N. Steunou, Metal-organic frameworks for the therapy of inflammatory diseases. Adv. Healthc. Mater. **14**(26), e2404334 (2025). 10.1002/adhm.202404334 40116524 10.1002/adhm.202404334

[28] S.S. Mohammed Ameen, A. Bedair, M. Hamed, F.R. Mansour, K.M. Omer, Recent advances in metal–organic frameworks as oxidase mimics: a comprehensive review on rational design and modification for enhanced sensing applications. ACS Appl. Mater. Interfaces **17**(1), 110–129 (2025). 10.1021/acsami.4c17397 10.1021/acsami.4c17397 39772422

[29] D. Ni, J. Lin, N. Zhang, S. Li, Y. Xue et al., Combinational application of metal–organic frameworks-based nanozyme and nucleic acid delivery in cancer therapy. WIREs Nanomed. Nanobiotechnol. **14**(3), e1773 (2022). 10.1002/wnan.1773 10.1002/wnan.1773 35014211

[30] L. Yang, S. Dong, S. Gai, D. Yang, H. Ding et al., Deep insight of design, mechanism, and cancer theranostic strategy of nanozymes. Nano-Micro Lett. **16**(1), 28 (2023). 10.1007/s40820-023-01224-0 10.1007/s40820-023-01224-0 PMC10663430 37989794

[31] D. Wang, D. Jana, Y. Zhao, Metal–organic framework derived nanozymes in biomedicine. Acc. Chem. Res. **53**(7), 1389–1400 (2020). 10.1021/acs.accounts.0c00268 32597637 10.1021/acs.accounts.0c00268

[32] Y. Zhao, J. Cheng, Z. Li, J. Wang, X. Chen, Nanozymes in biomedical applications: innovations originated from metal-organic frameworks. Adv. Healthc. Mater. **14**(8), e2402066 (2025). 10.1002/adhm.202402066 39319491 10.1002/adhm.202402066

[33] Y. Zhang, C. Zhang, W. Qian, F. Lei, Z. Chen et al., Recent advances in MOF-based nanozymes: synthesis, activities, and bioapplications. Biosens. Bioelectron. **263**, 116593 (2024). 10.1016/j.bios.2024.116593 39059178 10.1016/j.bios.2024.116593

[34] L. Ma, F. Jiang, X. Fan, L. Wang, C. He et al., Metal-organic-framework-engineered enzyme-mimetic catalysts. Adv. Mater. **32**(49), e2003065 (2020). 10.1002/adma.202003065 33124725 10.1002/adma.202003065

[35] H. Park, Y. Kang, J. Kim, Enhancing structure–property relationships in porous materials through transfer learning and cross-material few-shot learning. ACS Appl. Mater. Interfaces **15**(48), 56375–56385 (2023). 10.1021/acsami.3c10323 37983088 10.1021/acsami.3c10323

[36] S. Sheng, F. Liu, L. Lin, N. Yan, Y. Wang et al., Nanozyme-mediated cascade reaction based on metal-organic framework for synergetic chemo-photodynamic tumor therapy. J. Control. Release **328**, 631–639 (2020). 10.1016/j.jconrel.2020.09.029 32950593 10.1016/j.jconrel.2020.09.029

[37] J. Zhang, M. Guo, Q. He, Z. Zhang, B. Wu et al., Precise control of metal active sites of metal–organic framework nanozymes for achieving excellent enzyme-like activity and efficient pancreatitis therapy. Small **20**(32), 2310675 (2024). 10.1002/smll.202310675 10.1002/smll.202310675 38488710

[38] W. Zhang, Y. Liu, X. Wang, K. Qu, W. Zhu et al., A self-amplifying MOF nanoplatform for cancer immunotherapy synergizing starvation-enhanced cuproptosis and cGAS-STING activation. ACS Appl. Mater. Interfaces **17**(28), 40129–40142 (2025). 10.1021/acsami.5c07234 40592770 10.1021/acsami.5c07234

[39] J. Zhou, D. Xiong, H. Zhang, J. Xiao, R. Huang et al., Targeted enrichment of nucleic acid bionic arms enhances the hydrolysis activity of nanozymes for degradation and real-time monitoring of organophosphorus pesticides in water. Environ. Sci. Technol. **59**(3), 1844–1853 (2025). 10.1021/acs.est.4c13849 39813103 10.1021/acs.est.4c13849

[40] D. Gao, F. Wang, C. Zheng, B. Lv, J. Ma, A durable Ag@MOF-545/QCM-cotton fabric with “intelligent bacteria-capturing and dual antibacterial” properties. ACS Appl. Mater. Interfaces **17**(4), 6134–6143 (2025). 10.1021/acsami.4c21171 39813137 10.1021/acsami.4c21171

[41] F. Wang, M. Sun, D. Li, X. Qin, Y. Liao et al., Multifunctional asymmetric bacterial cellulose membrane with enhanced anti-bacterial and anti-inflammatory activities for promoting infected wound healing. Small **19**(48), 2303591 (2023). 10.1002/smll.202303591 10.1002/smll.202303591 37568253

[42] S. Zhong, F. Mo, L. Chen, W. Qin, L. Zhang et al., AgAu-modified quasi-MIL-53 hybrid nanozymes with triple enzyme-like activities for boosting biocatalytic disinfection. J. Colloid Interface Sci. **661**, 520–532 (2024). 10.1016/j.jcis.2024.01.190 38308892 10.1016/j.jcis.2024.01.190

[43] L. Liu, D. Chao, Q. Dong, X. Zhang, K. Zhang et al., Bimetallic NiCu-MOF protects DOX-induced myocardial injury and cardiac dysfunction by suppressing ferroptosis and inflammation. Adv. Healthc. Mater. **14**(11), 2405175 (2025). 10.1002/adhm.202405175 10.1002/adhm.202405175 40099577

[44] M. Ma, W. Yuan, W. Zhong, Y. Cheng, H. Yao et al., *In-situ* activation of biomimetic single-site bioorthogonal nanozyme for tumor-specific combination therapy. Biomaterials **312**, 122755 (2025). 10.1016/j.biomaterials.2024.122755 39151270 10.1016/j.biomaterials.2024.122755

[45] X. Jiang, W. Wang, J. Tang, M. Han, Y. Xu et al., Ligand-screened cerium-based MOF microcapsules promote nerve regeneration *via* mitochondrial energy supply. Adv. Sci. **11**(6), 2306780 (2024). 10.1002/advs.202306780 10.1002/advs.202306780 PMC10853750 38037294

[46] F. Yan, F. Cheng, C. Guo, G. Liang, S. Zhang et al., Curcumin-regulated constructing of defective zinc-based polymer-metal-organic framework as long-acting antibacterial platform for efficient wound healing. J. Colloid Interface Sci. **641**, 59–69 (2023). 10.1016/j.jcis.2023.03.050 36924546 10.1016/j.jcis.2023.03.050

[47] J. Wu, Y. Yu, Y. Cheng, C. Cheng, Y. Zhang et al., Ligand-dependent activity engineering of glutathione peroxidase-mimicking MIL-47(V) metal–organic framework nanozyme for therapy. Angew. Chem. Int. Ed. **60**(3), 1227–1234 (2021). 10.1002/anie.202010714 10.1002/anie.202010714 33022864

[48] S. Liu, Q. Meng, Z. Liu, J. Wang, J. Li et al., Engineered metal–organic framework with stereotactic anchoring and spatial separation of porphyrins for amplified ultrasound-mediated pyroptosis and cancer immunotherapy. Angew. Chem. Int. Ed. **64**(10), e202421402 (2025). 10.1002/anie.202421402 10.1002/anie.202421402 39573847

[49] Z. Zeng, M. Islamov, Y. He, B.A. Day, N.L. Rosi et al., Size-based norfentanyl detection with SWCNT@UiO-MOF composites. ACS Appl. Mater. Interfaces **16**(1), 1361–1369 (2024). 10.1021/acsami.3c17503 38147588 10.1021/acsami.3c17503 PMC10788826

[50] B. Ding, H. Chen, J. Tan, Q. Meng, P. Zheng et al., ZIF-8 nanoparticles evoke pyroptosis for high-efficiency cancer immunotherapy. Angew. Chem. Int. Ed. **62**(10), e202215307 (2023). 10.1002/anie.202215307 10.1002/anie.202215307 36629270

[51] J. Dong, X. Chai, Y. Xue, S. Shen, Z. Chen et al., ZIF-8-encapsulated pexidartinib delivery *via* targeted peptide-modified M1 macrophages attenuates MDSC-mediated immunosuppression in osteosarcoma. Small **20**(29), 2309038 (2024). 10.1002/smll.202309038 10.1002/smll.202309038 38456768

[52] H. Zhang, X.-B. Yin, Mixed-ligand metal–organic frameworks for all-in-one theranostics with controlled drug delivery and enhanced photodynamic therapy. ACS Appl. Mater. Interfaces **14**(23), 26528–26535 (2022). 10.1021/acsami.2c06873 35641317 10.1021/acsami.2c06873

[53] Z. Fan, S. Wu, H. Deng, G. Li, L. Huang et al., Light-triggered nanozymes remodel the tumor hypoxic and immunosuppressive microenvironment for ferroptosis-enhanced antitumor immunity. ACS Nano **18**(19), 12261–12275 (2024). 10.1021/acsnano.4c00844 38683132 10.1021/acsnano.4c00844

[54] S. Zhao, Y. Long, Y. Su, S. Wang, Z. Zhang et al., Cobalt-enhanced mass transfer and catalytic production of sulfate radicals in MOF-derived CeO_2_ · Co_3_O_4_ nanoflowers for efficient degradation of antibiotics. Small **17**(43), 2101393 (2021). 10.1002/smll.202101393 10.1002/smll.202101393 34160908

[55] L. Wang, Y. Wang, Y. Zhou, Bimetallic MOF-derived three-dimensional nanoflowers PdCoOx as peroxidase mimic activity for determining total antioxidant capacity. Food Chem. **457**, 140120 (2024). 10.1016/j.foodchem.2024.140120 38936126 10.1016/j.foodchem.2024.140120

[56] Y.-F. Xia, A. Sandra, H. Yu, H.-Q. Yuan, Z.-Q. Cai et al., Machine learning-assisted Eu-MOF fluorescent material for simultaneous monitoring and removal of malachite green. Biosens. Bioelectron. **287**, 117737 (2025). 10.1016/j.bios.2025.117737 40592262 10.1016/j.bios.2025.117737

[57] J. Liu, X. Meng, C. Hu, S. Wang, J. Tang et al., Metal covalent organic frameworks-based multi-signal nanozymes sensor array with machine learning for the intelligent recognition of sulfur-containing metallic salts. Sens. Actuators B Chem. **444**, 138426 (2025). 10.1016/j.snb.2025.138426

[58] Y. Song, J. Li, D. Chi, Z. Xu, J. Liu et al., AI-driven advances in metal–organic frameworks: from data to design and applications. Chem. Commun. **61**(82), 15972–16001 (2025). 10.1039/d5cc04220h 10.1039/d5cc04220h 41017480

[59] Z. Han, Y. Yang, J. Rushlow, J. Huo, Z. Liu et al., Development of the design and synthesis of metal–organic frameworks (MOFs)–from large scale attempts, functional oriented modifications, to artificial intelligence (AI) predictions. Chem. Soc. Rev. **54**(1), 367–395 (2025). 10.1039/d4cs00432a 39582426 10.1039/d4cs00432a

[60] A. Ozcan, F.-X. Coudert, S.M.J. Rogge, G. Heydenrych, D. Fan et al., Artificial intelligence paradigms for next-generation metal-organic framework research. J. Am. Chem. Soc. **147**(27), 23367–23380 (2025). 10.1021/jacs.5c08214 40551706 10.1021/jacs.5c08214 PMC12273595

[61] G. Zhang, W. Feng, G. Du, Y. Zhang, Y. Yang et al., Thermodynamically-driven phase engineering and reconstruction deduction of medium-entropy Prussian blue analogue nanocrystals. Adv. Mater. **37**(26), e2503814 (2025). 10.1002/adma.202503814 40223453 10.1002/adma.202503814

[62] Q. Li, Y. Zhang, X. Guo, Z. Yang, Y. Wang et al., Nucleation and growth mechanisms of micro/nano structural manganese-trimesic acid coordinations for aqueous zinc-ion batteries. Angew. Chem. Int. Ed. **64**(31), e202509741 (2025). 10.1002/anie.202509741 10.1002/anie.202509741 40406804

[63] Q. Li, Y. Zhang, W. Feng, J. Huang, S. Wei et al., Manganese–based metal–organic coordination for aqueous zinc–ion batteries with varying mechanical adaptability and machine learning–assisted performance decoding. Adv. Mater. **37**(35), 2507951 (2025). 10.1002/adma.202507951 10.1002/adma.202507951 40525761

[64] Y. Luo, S. Bag, O. Zaremba, A. Cierpka, J. Andreo et al., MOF synthesis prediction enabled by automatic data mining and machine learning. Angew. Chem. Int. Ed. **61**(19), e202200242 (2022). 10.1002/anie.202200242 10.1002/anie.202200242 PMC9310626 35104033

[65] K. Neikha, A. Puzari, Metal-organic frameworks through the lens of artificial intelligence: a comprehensive review. Langmuir **40**(42), 21957–21975 (2024). 10.1021/acs.langmuir.4c03126 39382843 10.1021/acs.langmuir.4c03126

[66] W. Gong, Z. Chen, J. Dong, Y. Liu, Y. Cui, Chiral metal–organic frameworks. Chem. Rev. **122**(9), 9078–9144 (2022). 10.1021/acs.chemrev.1c00740 35344663 10.1021/acs.chemrev.1c00740

[67] M. Qiao, Y. Li, Y. Li, M. Chang, X. Zhang et al., Unlocking of hidden mesopores for enzyme encapsulation by dynamic linkers in stable metal-organic frameworks. Angew. Chem. Int. Ed. **63**(51), e202409951 (2024). 10.1002/anie.202409951 10.1002/anie.202409951 39177482

[68] L. Chen, Y. Wang, E. Yuan, X. Hu, Y. Shu et al., An electron transfer regulation strategy to enhance the catalytic activity of perovskite fluorescent nanozyme by incorporation of Fe metal-organic framework for biomimetic cascade catalysis. Small **21**(35), 2505502 (2025). 10.1002/smll.202505502 10.1002/smll.202505502 40643062

[69] M. Xie, S. Chen, K. Chen, Q. Gao, J. Li et al., Metal-organic framework-based nanozymes: types, activity regulation and analytical applications. Analyst **150**(14), 3011–3025 (2025). 10.1039/d5an00365b 40583583 10.1039/d5an00365b

[70] Q. Li, Y. Cha, Y. Zhan, X. Miao, G. He et al., Atomic Ce-induced adaptive synergism for self-optimized multi-enzymatic nanozyme design for soil amendment. Small **21**(39), 2503939 (2025). 10.1002/smll.202503939 10.1002/smll.202503939 40511703

[71] C.H. Hendon, A. Walsh, Chemical principles underpinning the performance of the metal-organic framework HKUST-1. Chem. Sci. **6**(7), 3674–3683 (2015). 10.1039/c5sc01489a 28706713 10.1039/c5sc01489a PMC5496192

[72] K.E. DeKrafft, W.S. Boyle, L.M. Burk, O.Z. Zhou, W. Lin, Zr- and Hf-based nanoscale metal–organic frameworks as contrast agents for computed tomography. J. Mater. Chem. **22**(35), 18139–18144 (2012). 10.1039/c2jm32299d 23049169 10.1039/C2JM32299D PMC3462458

[73] S. Yu, H. Kang, S. Jee, W. Moon, D. Jang et al., MOF-based single-atom and metal cluster catalysts by room-temperature synthesis for tumor therapy. Adv. Healthc. Mater. **14**(18), e2501058 (2025). 10.1002/adhm.202501058 40424066 10.1002/adhm.202501058

[74] R. Zhang, S. Xie, X. Yang, Y. Liu, K. Lei et al., Au-Ag alloy nanourchins as a highly efficient SERS tag synergistically with MOF@Au for the ultrasensitive detection of oxytetracycline. J. Colloid Interface Sci. **695**, 137840 (2025). 10.1016/j.jcis.2025.137840 40359632 10.1016/j.jcis.2025.137840

[75] J. Xu, H. Chen, Y. Tao, H. Wang, Z. Wang et al., A dual-target recognition system based on acid-degradable Ni-MOF and aptamer guidance for precise tumor diagnosis and combined therapy. Adv. Sci. **12**(42), e12838 (2025). 10.1002/advs.202512838 10.1002/advs.202512838 PMC12622455 40990536

[76] C.-E. Choi, C. Liang, Y. Shamiya, S.J. Lee, A. Paul, Co-delivery of Ca-MOF and Mg-MOF using nanoengineered hydrogels to promote *in situ* mineralization and bone defect repair: *in vitro* and *in vivo* analysis. Adv. Healthc. Mater. **14**(32), e02630 (2025). 10.1002/adhm.202502630 40888230 10.1002/adhm.202502630 PMC12716217

[77] M. Kim, R. Xin, J. Earnshaw, J. Tang, J.P. Hill et al., MOF-derived nanoporous carbons with diverse tunable nanoarchitectures. Nat. Protoc. **17**(12), 2990–3027 (2022). 10.1038/s41596-022-00718-2 36064756 10.1038/s41596-022-00718-2

[78] A.F. Payam, S. Khalil, S. Chakrabarti, Synthesis and characterization of MOF-derived structures: recent advances and future perspectives. Small **20**(32), 2310348 (2024). 10.1002/smll.202310348 10.1002/smll.202310348 38660830

[79] S. Rojas-Buzo, B. Bohigues, C.W. Lopes, D.M. Meira, M. Boronat et al., Tailoring Lewis/Brønsted acid properties of MOF nodes *via* hydrothermal and solvothermal synthesis: simple approach with exceptional catalytic implications. Chem. Sci. **12**(29), 10106–10115 (2021). 10.1039/d1sc02833b 34349973 10.1039/d1sc02833b PMC8317639

[80] Z. Zheng, H.L. Nguyen, N. Hanikel, K.K. Li, Z. Zhou et al., High-yield, green and scalable methods for producing MOF-303 for water harvesting from desert air. Nat. Protoc. **18**(1), 136–156 (2023). 10.1038/s41596-022-00756-w 36289405 10.1038/s41596-022-00756-w

[81] X.-Y. Wang, K.-N. Jiao, Ying-Li, Y.-F. Huang, Z.-F. Wang, Mixed-valence Ce-Fe bimetallic MOFs with multi-enzyme-like activities for colorimetric biosensing and catalytic degradation. J. Colloid Interface Sci. **695**, 137784 (2025). 10.1016/j.jcis.2025.137784 10.1016/j.jcis.2025.137784 40354734

[82] B. Yu, Y. Bai, W. Gao, J. Wei, C. Gao et al., Colorimetric sensor array constructed based on bimetallic porphyrin-based metal organic framework nanozyme for the detection and recognition of tannic acid. Food Chem. **486**, 144592 (2025). 10.1016/j.foodchem.2025.144592 40334488 10.1016/j.foodchem.2025.144592

[83] W. Liang, P. Wied, F. Carraro, C.J. Sumby, B. Nidetzky et al., Metal–organic framework-based enzyme biocomposites. Chem. Rev. **121**(3), 1077–1129 (2021). 10.1021/acs.chemrev.0c01029 33439632 10.1021/acs.chemrev.0c01029

[84] J. Yi, G. Lee, S.S. Park, Solvent-induced structural rearrangement in ultrasound-assisted synthesis of metal-organic frameworks. Small Methods **8**(12), e2400363 (2024). 10.1002/smtd.202400363 38803311 10.1002/smtd.202400363

[85] L.-G. Qiu, Z.-Q. Li, Y. Wu, W. Wang, T. Xu et al., Facile synthesis of nanocrystals of a microporous metal–organic framework by an ultrasonic method and selective sensing of organoamines. Chem. Commun. **31**, 3642–3644 (2008). 10.1039/b804126a 10.1039/b804126a 18665285

[86] H. Heydarinasab, F.H. Sadeghi, H.E. Mohammadloo, B. Ramezanzadeh, Multi-metal/ligand MOFs: transformative materials for energy storage, photocatalysis, and sensor technologies. Adv. Colloid Interface Sci. **344**, 103592 (2025). 10.1016/j.cis.2025.103592 40627871 10.1016/j.cis.2025.103592

[87] M. Wen, N. Sun, L. Jiao, S.-Q. Zang, H.-L. Jiang, Microwave-assisted rapid synthesis of MOF-based single-atom Ni catalyst for CO_2_ electroreduction at ampere-level current. Angew. Chem. Int. Ed. **63**(10), e202318338 (2024). 10.1002/anie.202318338 10.1002/anie.202318338 38230982

[88] C.-J. Chang, Y.-C. Wang, Y.-H. Yu, Y.-C. Pu, W.-L. Kan, Morphology control and enhanced activity of (Cu–S)nMOF@ZnS heterostructures for photocatalytic H_2_ production. J. Colloid Interface Sci. **683**, 166–181 (2025). 10.1016/j.jcis.2024.12.166 39731861 10.1016/j.jcis.2024.12.166

[89] A. Ramos Corona, J. Rodríguez López, R. Rangel Segura, M.M. Martínez Garcia, E. Flores et al., Microwave-assisted synthesis of CdS-MOF MIL-101 (Fe) composite: characterization and photocatalytic performance. Inorg. Chem. **63**(42), 19536–19552 (2024). 10.1021/acs.inorgchem.4c02104 10.1021/acs.inorgchem.4c02104 PMC11497211 39374176

[90] Y. Liu, Y. Wei, M. Liu, Y. Bai, X. Wang et al., Electrochemical synthesis of large area two-dimensional metal–organic framework films on copper anodes. Angew. Chem. Int. Ed. **60**(6), 2887–2891 (2021). 10.1002/anie.202012971 10.1002/anie.202012971 33300656

[91] J.-O. Kim, W.-T. Koo, H. Kim, C. Park, T. Lee et al., Large-area synthesis of nanoscopic catalyst-decorated conductive MOF film using microfluidic-based solution shearing. Nat. Commun. **12**, 4294 (2021). 10.1038/s41467-021-24571-1 34257304 10.1038/s41467-021-24571-1 PMC8277906

[92] C. Liu, Q. Huang, LTP-assisted fabrication of laccase-like Cu-MOF nanozyme-encoded array sensor for identification and intelligent sensing of bioactive components in food. Biosens. Bioelectron. **267**, 116784 (2025). 10.1016/j.bios.2024.116784 39288708 10.1016/j.bios.2024.116784

[93] T. Friščić, C. Mottillo, H.M. Titi, Mechanochemistry for synthesis. Angew. Chem. Int. Ed. **59**(3), 1018–1029 (2020). 10.1002/anie.201906755 10.1002/anie.201906755 31294885

[94] S. Hwang, S. Grätz, L. Borchardt, A guide to direct mechanocatalysis. Chem. Commun. **58**(11), 1661–1671 (2022). 10.1039/d1cc05697b 10.1039/d1cc05697b PMC8812528 35023515

[95] K. Wang, Y. Zhang, Z. Gong, A. Li, Y. Ma et al., Monolayered metal–organic framework unlocks integration of shaped nanoparticles for synergistic photocatalysis. J. Am. Chem. Soc. **147**(28), 24241–24247 (2025). 10.1021/jacs.5c09403 40621780 10.1021/jacs.5c09403

[96] D. Chen, B. Chu, F. Li, Y.-T. Zheng, Y. Lu et al., Synergistic catalysis by Cu single atoms and atomically Cu-doped Au nanoparticles in a metal–organic framework for photocatalytic CO_2_ reduction to C_2_H_6_. J. Am. Chem. Soc. **147**(26), 22705–22713 (2025). 10.1021/jacs.5c04364 40526037 10.1021/jacs.5c04364

[97] J. Chen, G. Xu, R. Shen, J. Xu, C. Lu et al., Communications among neurocytes in Parkinson’s disease regulated by differential metabolism and blood-brain barrier traversing of chiral gold cluster-MOF integrated nanoparticles. Adv. Sci. **12**(23), 2500026 (2025). 10.1002/advs.202500026 10.1002/advs.202500026 PMC12199330 40365769

[98] J. Bujalance Fernández, V. de la Asunción-Nadal, B. Jurado Sánchez, A. Escarpa, Self-disassembling macroporous metal–organic framework-based micromotors with magnetically controlled motion for sequential drug release. Small Meth. **9**(10), 2500724 (2025). 10.1002/smtd.202500724 10.1002/smtd.202500724 PMC12536394 40459508

[99] W. Li, F. You, J. Yang, D. Gu, Y. Li et al., Antimicrobial peptide-targeted photodynamic therapy for preventing periodontal plaque biofilm formation through the disruption of quorum sensing system. Mater. Today Bio. **33**, 101970 (2025). 10.1016/j.mtbio.2025.101970 10.1016/j.mtbio.2025.101970 PMC12268586 40677408

[100] W. Yao, W. Liu, F. Su, J. Wang, H. Li et al., Hybrid membrane-camouflaged biomimetic immunomodulatory nanoturrets with sequential multidrug release for potentiating T cell-mediated anticancer immunity. J. Am. Chem. Soc. **146**(27), 18592–18605 (2024). 10.1021/jacs.4c04840 38943624 10.1021/jacs.4c04840

[101] Y. Zhang, J. Li, Q. Jing, Z. Chen, K. Wang et al., An erythrocyte membrane-derived nanosystem for efficient reversal of endothelial injury in sepsis. Adv. Healthc. Mater. **13**(3), 2302320 (2024). 10.1002/adhm.202302320 10.1002/adhm.202302320 37883686

[102] X. Zheng, Y. Zhao, Y. Jia, D. Shao, F. Zhang et al., Biomimetic co-assembled nanodrug of doxorubicin and berberine suppresses chemotherapy-exacerbated breast cancer metastasis. Biomaterials **271**, 120716 (2021). 10.1016/j.biomaterials.2021.120716 33621894 10.1016/j.biomaterials.2021.120716

[103] Y. Yao, X. Dong, Z. Pang, J. Shao, Z. He et al., A zinc-citrate metal–organic framework-based adaptable hydrogen sulfide delivery system for regulating neuroregeneration microenvironment in spinal cord injury. ACS Nano **19**(25), 22798–22819 (2025). 10.1021/acsnano.4c18918 40524455 10.1021/acsnano.4c18918

[104] G. An, H. Zheng, L. Guo, J. Huang, C. Yang et al., A metal-organic framework (MOF) built on surface-modified Cu nanoparticles eliminates tumors *via* multiple cascading synergistic therapeutic effects. J. Colloid Interface Sci. **662**, 298–312 (2024). 10.1016/j.jcis.2024.02.055 38354557 10.1016/j.jcis.2024.02.055

[105] B. Yang, H. Yao, J. Yang, C. Chen, J. Shi, Construction of a two-dimensional artificial antioxidase for nanocatalytic rheumatoid arthritis treatment. Nat. Commun. **13**(1), 1988 (2022). 10.1038/s41467-022-29735-1 35418125 10.1038/s41467-022-29735-1 PMC9008001

[106] M. Yin, T. Wang, Z. Wang, L. Lu, B. Ding et al., Synergistic self-calibration strategy based on nano-cornucopia MOFs for accurate HER-2 detection in precision breast cancer diagnosis. Biosens. Bioelectron. **288**, 117813 (2025). 10.1016/j.bios.2025.117813 40712488 10.1016/j.bios.2025.117813

[107] A. Tikhomirova, M.M. Rahman, S.P. Kidd, R.L. Ferrero, A. Roujeinikova, Cysteine and resistance to oxidative stress: implications for virulence and antibiotic resistance. Trends Microbiol. **32**(1), 93–104 (2024). 10.1016/j.tim.2023.06.010 37479622 10.1016/j.tim.2023.06.010

[108] L. Piacenza, A. Zeida, M. Trujillo, R. Radi, The superoxide radical switch in the biology of nitric oxide and peroxynitrite. Physiol. Rev. **102**(4), 1881–1906 (2022). 10.1152/physrev.00005.2022 35605280 10.1152/physrev.00005.2022

[109] Y. Ma, J. Pan, C. Ju, X. Yu, Y. Wang et al., Antioxidant nanozymes: current status and future perspectives in spinal cord injury treatments. Theranostics **15**(13), 6146–6183 (2025). 10.7150/thno.114836 40521206 10.7150/thno.114836 PMC12159832

[110] Y. Yu, P. Li, L. Bao, F. Liu, Z. Zeng et al., An injectable, self-adaptive hydrogel with metallic-functionalized metal organic frameworks for enhanced wound healing in dynamic infectious and inflammatory microenvironment. Small Methods **9**(8), 2500015 (2025). 10.1002/smtd.202500015 10.1002/smtd.202500015 40181600

[111] P. Yang, J. Tao, F. Chen, Y. Chen, J. He et al., Multienzyme-mimic ultrafine alloyed nanoparticles in metal organic frameworks for enhanced chemodynamic therapy. Small **17**(7), 2005865 (2021). 10.1002/smll.202005865 10.1002/smll.202005865 33502106

[112] Y. Liu, H. Li, W. Liu, J. Guo, H. Yang et al., Design of monovalent cerium-based metal organic frameworks as bioinspired superoxide dismutase mimics for ionizing radiation protection. ACS Appl. Mater. Interfaces **14**(49), 54587–54597 (2022). 10.1021/acsami.2c17358 36468174 10.1021/acsami.2c17358

[113] Y. Liu, R. Niu, Y. Wang, H. Zhang, Y. Zhao, Preparation and biomedical applications of single-metal atom catalysts. Nat. Protoc. **21**(2), 775–807 (2026). 10.1038/s41596-025-01199-9 40542217 10.1038/s41596-025-01199-9

[114] T. Rajarathinam, S. Jayaraman, C.-S. Kim, J.-H. Yoon, S.-C. Chang, Two-dimensional nanozyme nanoarchitectonics customized electrochemical bio diagnostics and lab-on-chip devices for biomarker detection. Adv. Colloid Interface Sci. **341**, 103474 (2025). 10.1016/j.cis.2025.103474 40121951 10.1016/j.cis.2025.103474

[115] Y. Gong, Y. Xiao, C. Zhao, H. Deng, H. Liu et al., Ultrasmall PtIr bimetallic nanozyme treats myocardial infarction *via* ischemic/inflammatory cardiac microenvironment remodeling. ACS Nano **19**(14), 13723–13739 (2025). 10.1021/acsnano.4c14869 40175295 10.1021/acsnano.4c14869

[116] B. Yu, W. Sun, J. Lin, C. Fan, C. Wang et al., Using Cu-based metal–organic framework as a comprehensive and powerful antioxidant nanozyme for efficient osteoarthritis treatment. Adv. Sci. **11**(13), 2307798 (2024). 10.1002/advs.202307798 10.1002/advs.202307798 PMC10987124 38279574

[117] N.L. Reed, T.P. Yoon, Oxidase reactions in photoredox catalysis. Chem. Soc. Rev. **50**(5), 2954–2967 (2021). 10.1039/d0cs00797h 33491681 10.1039/d0cs00797h PMC8571993

[118] Z. Li, M. Shen, F. Meng, Y. Zhang, W. Duan et al., Engineering oxidase-based cascade nanoreactors design, catalytic efficiency, and applications in disease monitoring. Small **21**(27), e2501976 (2025). 10.1002/smll.202501976 40351055 10.1002/smll.202501976

[119] L. Zheng, Y. Zhang, R. Shi, X. Xue, K. Li et al., Nanohybrid urate oxidase with magnetically switchable catalytic potential for precise gout therapy. Biomaterials **320**, 123277 (2025). 10.1016/j.biomaterials.2025.123277 40127507 10.1016/j.biomaterials.2025.123277

[120] X. Zhou, Z. Shao, S. Yan, Y. Lin, Y. Liu et al., Coencapsulating TMB probes and bimetallic MOF nanozymes in a hydrogel patch for fabricating reusable visual VC sensors. Anal. Chem. **96**(43), 17310–17318 (2024). 10.1021/acs.analchem.4c03665 39412411 10.1021/acs.analchem.4c03665

[121] Z. Wei, L. Yu, Y. Feng, Z. Gan, Y. Shen et al., Bioinspired heterocoordination in adaptable cobalt metal-organic framework for DNA epigenetic modification detection. Anal. Chem. **96**(24), 9984–9993 (2024). 10.1021/acs.analchem.4c01377 38833588 10.1021/acs.analchem.4c01377

[122] X. Wan, L. Song, W. Pan, H. Zhong, N. Li et al., Tumor-targeted cascade nanoreactor based on metal–organic frameworks for synergistic ferroptosis–starvation anticancer therapy. ACS Nano **14**(9), 11017–11028 (2020). 10.1021/acsnano.9b07789 32786253 10.1021/acsnano.9b07789

[123] R. He, L. Guo, X. Kou, R. Gao, W. Huang et al., Hierarchically macroporous Ce-MOF nanozyme with enhanced phosphoester hydrolase- and oxidase-like activities for self-cascade colorimetric detection of profenofos on-site. Anal. Chem. **97**(21), 11221–11230 (2025). 10.1021/acs.analchem.5c01247 40405505 10.1021/acs.analchem.5c01247

[124] G. Li, H. Liu, T. Hu, F. Pu, J. Ren et al., Dimensionality engineering of single-atom nanozyme for efficient peroxidase-mimicking. J. Am. Chem. Soc. **145**(30), 16835–16842 (2023). 10.1021/jacs.3c05162 37487021 10.1021/jacs.3c05162

[125] X. Li, X. Zhang, L. Song, Y. Li, A. Liu et al., Nanozyme as tumor energy homeostasis disruptor to augment cascade catalytic therapy. ACS Nano **18**(51), 34656–34670 (2024). 10.1021/acsnano.4c09982 39661982 10.1021/acsnano.4c09982

[126] Y.-J. Wei, J. Li, Z.-E. Hu, X. Xing, Z.-W. Zhou et al., A porphyrin-MOF-based integrated nanozyme system for catalytic cascades and light-enhanced synergistic amplification of cellular oxidative stress. J. Mater. Chem. B **11**(28), 6581–6594 (2023). 10.1039/d3tb00681f 37358033 10.1039/d3tb00681f

[127] C. Hu, S. Gao, X. Li, K. Yang, Y. Cheng et al., Crosstalk of autophagy and ferroptosis in cardiovascular diseases: from pathophysiology to novel therapy. Redox Biol. **84**, 103705 (2025). 10.1016/j.redox.2025.103705 40450834 10.1016/j.redox.2025.103705 PMC12164230

[128] Q. Lu, Y. Ding, W. Liu, S. Liu, Viral infections and the glutathione peroxidase family: mechanisms of disease development. Antioxid. Redox Signal. **42**(10–12), 623–639 (2025). 10.1089/ars.2024.0645 39446976 10.1089/ars.2024.0645

[129] Y. Wu, W. Chen, C. Wang, D. Xing, Overview of nanozymes with phosphatase-like activity. Biosens. Bioelectron. **237**, 115470 (2023). 10.1016/j.bios.2023.115470 37413827 10.1016/j.bios.2023.115470

[130] X. Mao, F. He, D. Qiu, S. Wei, R. Luo et al., Efficient biocatalytic system for biosensing by combining metal-organic framework (MOF)-based nanozymes and G-quadruplex (G4)-DNAzymes. Anal. Chem. **94**(20), 7295–7302 (2022). 10.1021/acs.analchem.2c00600 35549161 10.1021/acs.analchem.2c00600

[131] K. Ma, Y.H. Cheung, K.O. Kirlikovali, H. Xie, K.B. Idrees et al., Fibrous Zr-MOF nanozyme aerogels with macro-nanoporous structure for enhanced catalytic hydrolysis of organophosphate toxins. Adv. Mater. **36**(10), 2300951 (2024). 10.1002/adma.202300951 10.1002/adma.202300951 37310697

[132] Y. Zhu, D. He, Z. Hou, M. Lan, Y. Zhang et al., Clinical features, molecular biology, and the metastatic microenvironment in lung cancer brain metastases: implications for treatment decisions. Adv. Sci. **12**(33), e02626 (2025). 10.1002/advs.202502626 10.1002/advs.202502626 PMC12412593 40719284

[133] Y. Lu, J. Zheng, P. Lin, Y. Lin, Y. Zheng et al., Tumor microenvironment-derived exosomes: a double-edged sword for advanced T cell-based immunotherapy. ACS Nano **18**(40), 27230–27260 (2024). 10.1021/acsnano.4c09190 39319751 10.1021/acsnano.4c09190

[134] Z. Hu, H. Tan, Y. Ye, W. Xu, J. Gao et al., NIR-actuated ferroptosis nanomotor for enhanced tumor penetration and therapy. Adv. Mater. **36**(49), e2412227 (2024). 10.1002/adma.202412227 39370589 10.1002/adma.202412227

[135] Y. Li, W. Chen, Y. Kang, X. Zhen, Z. Zhou et al., Nanosensitizer-mediated augmentation of sonodynamic therapy efficacy and antitumor immunity. Nat. Commun. **14**(1), 6973 (2023). 10.1038/s41467-023-42509-7 37914681 10.1038/s41467-023-42509-7 PMC10620173

[136] L. Li, J. Li, R. Hu, X. Zhang, L. Ding et al., Tumor cell targeting and responsive nanoplatform for multimodal-imaging guided chemodynamic/photodynamic/photothermal therapy toward triple negative breast cancer. ACS Appl. Mater. Interfaces **15**(23), 27706–27718 (2023). 10.1021/acsami.3c04709 37261936 10.1021/acsami.3c04709

[137] Y. Ding, H. Xu, C. Xu, Z. Tong, S. Zhang et al., A nanomedicine fabricated from gold nanoparticles-decorated metal–organic framework for cascade chemo/chemodynamic cancer therapy. Adv. Sci. **7**(17), 2001060 (2020). 10.1002/advs.202001060 10.1002/advs.202001060 PMC7507500 32995124

[138] Y. Cheng, Y.-D. Xia, Y.-Q. Sun, Y. Wang, X.-B. Yin, “Three-in-one” nanozyme composite for augmented cascade catalytic tumor therapy. Adv. Mater. **36**(8), e2308033 (2024). 10.1002/adma.202308033 37851918 10.1002/adma.202308033

[139] Y.-Y. Zhao, L. Lu, H. Jeong, H. Kim, X. Li et al., Enhancing biosafety in photodynamic therapy: progress and perspectives. Chem. Soc. Rev. **54**(17), 7749–7768 (2025). 10.1039/d5cs00054h 40734631 10.1039/d5cs00054h

[140] R. Bai, Y. Li, L. Jian, Y. Yang, L. Zhao et al., The hypoxia-driven crosstalk between tumor and tumor-associated macrophages: mechanisms and clinical treatment strategies. Mol. Cancer **21**(1), 177 (2022). 10.1186/s12943-022-01645-2 36071472 10.1186/s12943-022-01645-2 PMC9454207

[141] R. Wang, M. Qiu, L. Zhang, M. Sui, L. Xiao et al., Augmenting immunotherapy *via* bioinspired MOF-based ROS homeostasis disruptor with nanozyme-cascade reaction. Adv. Mater. **35**(49), 2306748 (2023). 10.1002/adma.202306748 10.1002/adma.202306748 37689996

[142] M.-M. Pan, P. Li, Y.-P. Yu, M. Jiang, X. Yang et al., Bimetallic ions functionalized metal–organic-framework nanozyme for tumor microenvironment regulating and enhanced photodynamic therapy for hypoxic tumor. Adv. Healthc. Mater. **12**(26), 2300821 (2023). 10.1002/adhm.202300821 10.1002/adhm.202300821 37199497

[143] M. Yang, X. Wang, M. Peng, F. Wang, S. Hou et al., Nanomaterials enhanced sonodynamic therapy for multiple tumor treatment. Nano-Micro Lett. **17**(1), 157 (2025). 10.1007/s40820-025-01666-8 10.1007/s40820-025-01666-8 PMC11850698 39992547

[144] J. Liang, X. Qiao, L. Qiu, H. Xu, H. Xiang et al., Engineering versatile nanomedicines for ultrasonic tumor immunotherapy. Adv. Sci. **11**(3), 2305392 (2024). 10.1002/advs.202305392 10.1002/advs.202305392 PMC10797440 38041509

[145] Q. Ren, N. Yu, L. Wang, M. Wen, P. Geng et al., Nanoarchitectonics with metal-organic frameworks and platinum nanozymes with improved oxygen evolution for enhanced sonodynamic/chemo-therapy. J. Colloid Interface Sci. **614**, 147–159 (2022). 10.1016/j.jcis.2022.01.050 35091144 10.1016/j.jcis.2022.01.050

[146] S. Xu, H. Zhang, Z. Qian, W. Yuan, pH-responsive injectable self-healing hydrogels loading Au nanoparticles-decorated bimetallic organic frameworks for synergistic sonodynamic-chemodynamic-starvation-chemo therapy of cancer. J. Colloid Interface Sci. **675**, 746–760 (2024). 10.1016/j.jcis.2024.07.039 38996704 10.1016/j.jcis.2024.07.039

[147] L. Cai, J. Du, F. Han, T. Shi, H. Zhang et al., Piezoelectric metal-organic frameworks based sonosensitizer for enhanced nanozyme catalytic and sonodynamic therapies. ACS Nano **17**(8), 7901–7910 (2023). 10.1021/acsnano.3c01856 37052950 10.1021/acsnano.3c01856

[148] W. Zhen, T. Zhao, X. Chen, J. Zhang, Unlocking the potential of disulfidptosis: nanotechnology-driven strategies for advanced cancer therapy. Small **21**(23), 2500880 (2025). 10.1002/smll.202500880 10.1002/smll.202500880 40269657

[149] Z. Zhang, Y. Pan, X. Fan, N. Du, Q. Pan et al., A colon-targeted oral nanosystem disrupts the inflammatory loop in enteric *Glia* to alleviate ulcerative colitis. ACS Nano **19**(31), 28242–28256 (2025). 10.1021/acsnano.5c05032 40719097 10.1021/acsnano.5c05032

[150] J. Liu, J. Liang, J. Xue, K. Liang, Metal–organic frameworks as a versatile materials platform for unlocking new potentials in biocatalysis. Small **17**(32), 2100300 (2021). 10.1002/smll.202100300 10.1002/smll.202100300 33949785

[151] C. Zhang, X. Zhang, F. Li, B. Li, M. Zhang et al., Thermosensitive hydrogel integrated with bimetallic nano-enzymes for modulating the microenvironment in diabetic wound beds. Adv. Sci. **12**(6), 2411575 (2025). 10.1002/advs.202411575 10.1002/advs.202411575 PMC11809323 39686701

[152] F. Lv, H. Fang, L. Huang, Q. Wang, S. Cao et al., Curcumin equipped nanozyme-like Metal−Organic framework platform for the targeted atherosclerosis treatment with lipid regulation and enhanced magnetic resonance imaging capability. Adv. Sci. **11**(26), 2309062 (2024). 10.1002/advs.202309062 10.1002/advs.202309062 PMC11234396 38696653

[153] K. Xiang, H. Wu, Y. Liu, S. Wang, X. Li et al., MOF-derived bimetallic nanozyme to catalyze ROS scavenging for protection of myocardial injury. Theranostics **13**(8), 2721–2733 (2023). 10.7150/thno.83543 37215581 10.7150/thno.83543 PMC10196836

[154] Q. Chen, J. Wang, X. Xiong, J. Chen, B. Wang et al., Blood-brain barrier-penetrating metal-organic framework antioxidant nanozymes for targeted ischemic stroke therapy. Adv. Healthc. Mater. **14**(26), 2402376 (2025). 10.1002/adhm.202402376 10.1002/adhm.202402376 39373278

[155] Q. Li, X. Ding, Z. Chang, X. Fan, J. Pan et al., Metal–organic framework based nanozyme system for NLRP3 inflammasome-mediated neuroinflammatory regulation in Parkinson’s disease. Adv. Healthc. Mater. **13**(10), 2303454 (2024). 10.1002/adhm.202303454 10.1002/adhm.202303454 38031989

[156] W. Jiang, Q. Li, R. Zhang, J. Li, Q. Lin et al., Chiral metal-organic frameworks incorporating nanozymes as neuroinflammation inhibitors for managing Parkinson’s disease. Nat. Commun. **14**(1), 8137 (2023). 10.1038/s41467-023-43870-3 38065945 10.1038/s41467-023-43870-3 PMC10709450

[157] S. Ge, A. Sun, X. Zhou, P. Niu, Y. Chen et al., Functionalized nanozyme microcapsules targeting deafness prevention *via* mitochondrial homeostasis remodeling. Adv. Mater. **37**(5), e2413371 (2025). 10.1002/adma.202413371 39663676 10.1002/adma.202413371

[158] H. Zhang, Q. Liang, Y. Ji, Q. Chen, W. Jiang et al., Facile fabrication of antioxidative and antibacterial hydrogel films to accelerate infected diabetic wound healing. Bioact. Mater. **53**, 386–403 (2025). 10.1016/j.bioactmat.2025.07.026 40735354 10.1016/j.bioactmat.2025.07.026 PMC12305319

[159] Y.-J. Wei, H. Chen, Z.-W. Zhou, C.-X. Liu, C.-X. Cai et al., Kill two birds with one stone: dual-metal MOF-nanozyme-decorated hydrogels with ROS-scavenging, oxygen-generating, and antibacterial abilities for accelerating infected diabetic wound healing. Small **20**(48), 2403679 (2024). 10.1002/smll.202403679 10.1002/smll.202403679 39240068

[160] D. Chao, Q. Dong, Z. Yu, D. Qi, M. Li et al., Specific nanodrug for diabetic chronic wounds based on antioxidase-mimicking MOF-818 nanozymes. J. Am. Chem. Soc. **144**(51), 23438–23447 (2022). 10.1021/jacs.2c09663 36512736 10.1021/jacs.2c09663

[161] J. Liu, Z. Chen, H. Liu, S. Qin, M. Li et al., Nickel-based metal-organic frameworks promote diabetic wound healing *via* scavenging reactive oxygen species and enhancing angiogenesis. Small **20**(10), 2305076 (2024). 10.1002/smll.202305076 10.1002/smll.202305076 37909382

[162] J. Liu, X. Han, T. Zhang, K. Tian, Z. Li et al., Reactive oxygen species (ROS) scavenging biomaterials for anti-inflammatory diseases: from mechanism to therapy. J. Hematol. Oncol. **16**(1), 116 (2023). 10.1186/s13045-023-01512-7 38037103 10.1186/s13045-023-01512-7 PMC10687997

[163] S.J. Forrester, D.S. Kikuchi, M.S. Hernandes, Q. Xu, K.K. Griendling, Reactive oxygen species in metabolic and inflammatory signaling. Circ. Res. **122**(6), 877–902 (2018). 10.1161/circresaha.117.311401 29700084 10.1161/CIRCRESAHA.117.311401 PMC5926825

[164] B. Yu, W. Sun, J. Lin, C. Fan, C. Wang et al., Using Cu-based metal–organic framework as a comprehensive and powerful antioxidant nanozyme for efficient osteoarthritis treatment. Adv. Sci. **11**(13), 2307798 (2024). 10.1002/advs.20230779 10.1002/advs.202307798 PMC10987124 38279574

[165] C. Zhu, K. Huang, T. Li, Y. Li, Y. Jin et al., Manganese dioxide coupled metal-organic framework as mitophagy regulator alleviates periodontitis through SIRT1-FOXO_3_^-^BNIP3 signaling axis. Biomaterials **319**, 123179 (2025). 10.1016/j.biomaterials.2025.123179 39983516 10.1016/j.biomaterials.2025.123179

[166] Y. Li, Y. Li, H. Chen, Y. Chen, Y. Ni et al., Self-assembled copper-amino acid nanoleaves for targeted treatment of deep-seated bacterial infections *via* chemodynamic therapy and cuproptosis-like death. Biomaterials **325**, 123566 (2026). 10.1016/j.biomaterials.2025.123566 40700980 10.1016/j.biomaterials.2025.123566

[167] Y. Xue, X. Chen, F. Wu, C. Chen, N. Lin et al., A self-oxidizing o-dihydroxybenzene-based covalent organic framework hydrogel with broad-spectrum antibacterial properties for promoting diabetic wound healing. Adv. Funct. Mater. **35**(42), 2505669 (2025). 10.1002/adfm.202505669

[168] Y. Wang, W. Guo, K. Zhang, Z. Liu, X. Dai et al., Biomimetic electrodynamic metal-organic framework nanosponges for augmented treatment of biofilm infections. Adv. Sci. **11**(46), 2408442 (2024). 10.1002/advs.202408442 10.1002/advs.202408442 PMC11633466 39422163

[169] M. Ge, W. Zhu, J. Mei, T. Hu, C. Yang et al., Piezoelectric-enhanced nanocatalysts trigger neutrophil N1 polarization against bacterial biofilm by disrupting redox homeostasis (adv. mater. 6/2025). Adv. Mater. **37**(6), 2570050 (2025). 10.1002/adma.202570050 10.1002/adma.202409633 39350533

[170] Y. Yu, T. Cui, C. Liu, W. Yang, B. Zhang, Tunable hierarchically porous gadolinium-based metal-organic frameworks for bacteria-targeting magnetic resonance imaging and *in situ* anti-bacterial therapy. Adv. Sci. **12**(15), 2415209 (2025). 10.1002/advs.202415209 10.1002/advs.202415209 PMC12005816 39976077

[171] W. Zhang, B. Wang, G. Xiang, T. Jiang, X. Zhao, Photodynamic alginate Zn-MOF thermosensitive hydrogel for accelerated healing of infected wounds. ACS Appl. Mater. Interfaces **15**(19), 22830–22842 (2023). 10.1021/acsami.2c23321 37129874 10.1021/acsami.2c23321

[172] F. Mo, C. Lin, J. Lu, D. Sun, Integrating artificial DNAzymes with natural enzymes on 2D MOF hybrid nanozymes for enhanced treatment of bacteria-infected wounds. Small **20**(21), 2307256 (2024). 10.1002/smll.202307256 10.1002/smll.202307256 38018326

[173] L. Wu, Y. Luo, C. Wang, S. Wu, Y. Zheng et al., Self-driven electron transfer biomimetic enzymatic catalysis of bismuth-doped PCN-222 MOF for rapid therapy of bacteria-infected wounds. ACS Nano **17**(2), 1448–1463 (2023). 10.1021/acsnano.2c10203 10.1021/acsnano.2c10203 36622022

[174] S.Z. Khairunnisa, O. Guselnikova, Y. Kang, P.S. Postnikov, R.R. Valiev et al., Hyperuniform mesoporous gold films coated with halogen-bonding metal–organic frameworks for selective Raman sensing of chlorinated hydrocarbons. ACS Nano **19**(30), 27890–27901 (2025). 10.1021/acsnano.5c09431 40704751 10.1021/acsnano.5c09431 PMC12333424

[175] Z. Zhang, J. Zhang, X. Wang, X. Zhang, W. Li et al., Catching like an *Octopus*: based on structural biomimetic designed MOF-on-MOF compatible extracted fiber for sensitive detection and risk assessment of organophosphorus pesticides. Small **21**(35), 2506627 (2025). 10.1002/smll.202506627 10.1002/smll.202506627 40635242

[176] J. Liu, C. Hu, X. Meng, Y. Sun, B. Zhao et al., Metal covalent organic frameworks-based laccase-like nanozyme for oxidative degradation and identification of phenolic pollutants. J. Hazard. Mater. **487**, 137142 (2025). 10.1016/j.jhazmat.2025.137142 39823869 10.1016/j.jhazmat.2025.137142

[177] S. Mu, L. Ji, Y. Rao, Z. Feng, J. Zhu et al., Os-single-atom anchored artificial enzymes with POD-mimetic activities as smartphone-based biosensor for the visual and multimodal detection of food additives. Small Methods **9**(9), e00976 (2025). 10.1002/smtd.202500976 40693310 10.1002/smtd.202500976

[178] N. Jin, F. Yang, X. Zhang, Y. Li, J. Lin, Sensitive detection of multiplex bacteria based on finger driven microfluidics and recombinase aided amplification. Biosens. Bioelectron. **287**, 117750 (2025). 10.1016/j.bios.2025.117750 40639142 10.1016/j.bios.2025.117750

[179] C. Zhao, S. Liu, W. Dang, Q. Liu, D. Yin et al., Enzyme-activated biosensor assisted by enzymatic rolling circle amplification for sensitive detection of APE1 and imaging *in vivo*. Anal. Chem. **97**(30), 16690–16697 (2025). 10.1021/acs.analchem.5c03866 40761079 10.1021/acs.analchem.5c03866

[180] J.-L. Zhang, S. Gao, Y. Yang, W.-B. Liang, M.-L. Lu et al., Ruthenium(II) complex-grafted conductive metal-organic frameworks with conductivity- and confinement-enhanced electrochemiluminescence for ultrasensitive biosensing application. Biosens. Bioelectron. **227**, 115157 (2023). 10.1016/j.bios.2023.115157 36841115 10.1016/j.bios.2023.115157

[181] X.-M. Shi, Z. Wang, M.-H. Chen, Q.-Q. Wu, F.-Z. Chen et al., Highly light-harvesting MOF-on-MOF heterostructure: cascading functionality to flexible photogating of organic photoelectrochemical transistor and bienzyme cascade detection. Anal. Chem. **96**(8), 3679–3685 (2024). 10.1021/acs.analchem.4c00173 38353671 10.1021/acs.analchem.4c00173

[182] H. Yang, J. Zheng, W. Wang, J. Lin, J. Wang et al., Zr-MOF carrier-enhanced dual-mode biosensing platforms for rapid and sensitive diagnosis of mpox. Adv. Sci. **11**(38), 2405848 (2024). 10.1002/advs.202405848 10.1002/advs.202405848 PMC11481339 39119886

[183] Z. Wu, J. Wang, X. Cheng, Y. Tang, L. Xia et al., A novel fluorescent sensor for highly sensitive detection of ascorbic acid in food based on inhibiting phosphatase-like activity of Zr-based MOF. Food Chem. **471**, 142837 (2025). 10.1016/j.foodchem.2025.142837 39808980 10.1016/j.foodchem.2025.142837

[184] H. Zhu, B. Liu, J. Pan, L. Xu, J. Liu et al., Redox interference-free bimodal paraoxon sensing enabled by an aggregation-induced emission nanozyme catalytically hydrolyzing phosphoesters specifically. Biosens. Bioelectron. **267**, 116756 (2025). 10.1016/j.bios.2024.116756 39244836 10.1016/j.bios.2024.116756

[185] B. Sun, V. Panferov, X. Guo, J. Xiong, S. Zhang et al., A novel triple-signal biosensor based on ZrFe-MOF@PtNPs for ultrasensitive aflatoxins detection. Biosens. Bioelectron. **267**, 116797 (2025). 10.1016/j.bios.2024.116797 39307032 10.1016/j.bios.2024.116797

[186] J. You, S. Yang, F. Chai, K. Zheng, M. Tian, Dual-mode smart sensing of sarcosine by integrating nanozyme-mediated electrochemical and smartphone-readable colorimetric assays. Biosens. Bioelectron. **295**, 118322 (2026). 10.1016/j.bios.2025.118322 41436348 10.1016/j.bios.2025.118322

[187] R. Ettlinger, U. Lächelt, R. Gref, P. Horcajada, T. Lammers et al., Toxicity of metal–organic framework nanoparticles: from essential analyses to potential applications. Chem. Soc. Rev. **51**(2), 464–484 (2022). 10.1039/d1cs00918d 34985082 10.1039/d1cs00918d

[188] C. Yang, H. Ming, B. Li, S. Liu, L. Chen et al., A pH and glutathione-responsive carbon monoxide-driven nano-herb delivery system for enhanced immunotherapy in colorectal cancer. J. Control. Release **376**, 659–677 (2024). 10.1016/j.jconrel.2024.10.043 39442888 10.1016/j.jconrel.2024.10.043

[189] M.A. Luzuriaga, R.P. Welch, M. Dharmarwardana, C.E. Benjamin, S. Li et al., Enhanced stability and controlled delivery of MOF-encapsulated vaccines and their immunogenic response *in vivo*. ACS Appl. Mater. Interfaces **11**(10), 9740–9746 (2019). 10.1021/acsami.8b20504 30776885 10.1021/acsami.8b20504

[190] X. Lu, W. Deng, S. Wang, S. Zhao, B. Zhu et al., PEGylated Elesclomol@Cu(Ⅱ)-based metal-organic framework with effective nanozyme performance and cuproptosis induction efficacy for enhanced PD-L1-based immunotherapy. Mater. Today Bio **29**, 101317 (2024). 10.1016/j.mtbio.2024.101317 10.1016/j.mtbio.2024.101317 PMC11565527 39554839

[191] H. Liang, F. Lin, Z. Zhang, B. Liu, S. Jiang et al., Multicopper laccase mimicking nanozymes with nucleotides as ligands. ACS Appl. Mater. Interfaces **9**(2), 1352–1360 (2017). 10.1021/acsami.6b15124 28004568 10.1021/acsami.6b15124

[192] C. Liu, L. Luo, L. Zeng, J. Xing, Y. Xia et al., Porous gold nanoshells on functional NH_2_-MOFs: facile synthesis and designable platforms for cancer multiple therapy. Small **14**(35), 1801851 (2018). 10.1002/smll.201801851 10.1002/smll.201801851 30058139

[193] Z. Liu, F. Wang, J. Ren, X. Qu, A series of MOF/Ce-based nanozymes with dual enzyme-like activity disrupting biofilms and hindering recolonization of bacteria. Biomaterials **208**, 21–31 (2019). 10.1016/j.biomaterials.2019.04.007 30986610 10.1016/j.biomaterials.2019.04.007

[194] X. Li, X. Li, D. Li, M. Zhao, H. Wu et al., Electrochemical biosensor for ultrasensitive exosomal miRNA analysis by cascade primer exchange reaction and MOF@Pt@MOF nanozyme. Biosens. Bioelectron. **168**, 112554 (2020). 10.1016/j.bios.2020.112554 32871496 10.1016/j.bios.2020.112554

[195] H. Yang, Z. Sun, X. Qin, H. Wu, H. Zhang et al., Ultrasmall Au nanoparticles modified 2D metalloporphyrinic metal-organic framework nanosheets with high peroxidase-like activity for colorimetric detection of organophosphorus pesticides. Food Chem. **376**, 131906 (2022). 10.1016/j.foodchem.2021.131906 34968912 10.1016/j.foodchem.2021.131906

[196] S. Li, Z. Zhou, Z. Tie, B. Wang, M. Ye et al., Data-informed discovery of hydrolytic nanozymes. Nat. Commun. **13**(1), 827 (2022). 10.1038/s41467-022-28344-2 35149676 10.1038/s41467-022-28344-2 PMC8837776

[197] Q. Shi, Y. Zhao, M. Liu, F. Shi, L. Chen et al., Engineering platelet membrane-coated bimetallic MOFs as biodegradable nanozymes for efficient antibacterial therapy. Small **20**(23), 2309366 (2024). 10.1002/smll.202309366 10.1002/smll.202309366 38150620

[198] Y. Bai, S. Nie, W. Gao, N. Li, P. Zhu et al., Enzyme-nanozyme cascade flow reactor synergy with deep learning for differentiation and point-of-care testing of multiple organophosphorus pesticides. Adv. Funct. Mater. **35**(17), 2419499 (2025). 10.1002/adfm.202419499

[199] S. Liu, R. Xin, X. Zhang, L. Han, Separable microneedle patch integrated with the dictamnine-loaded copper MOF nanozyme for atopic dermatitis treatment. ACS Appl. Mater. Interfaces **17**(18), 26386–26401 (2025). 10.1021/acsami.5c02334 40273362 10.1021/acsami.5c02334

[200] C. Yao, R. Zhang, Z. Xie, Y. Wu, X. Wu, A magnetically actuated MOF-based nanozyme for intensified induction of ferroptosis and immunogenic cell death *via* autophagy blockade. Small **21**(5), 2409026 (2025). 10.1002/smll.202409026 10.1002/smll.202409026 39659092

[201] R. Li, W. Zhao, Z. Han, N. Feng, T. Wu et al., Self-cascade nanozyme reactor as a cuproptosis inducer synergistic inhibition of cellular respiration boosting radioimmunotherapy. Small **20**(25), 2306263 (2024). 10.1002/smll.202306263 10.1002/smll.202306263 38221757

[202] K. Yu, X. Li, S. Zhan, H. Chai, X. Cao et al., Programmable bimetallic MOF nanozyme composite with microenvironment-adaptive cascade catalysis for wearable glucose biosensing and accelerated diabetic wound healing. Anal. Chem. **97**(39), 21668–21678 (2025). 10.1021/acs.analchem.5c04419 41003546 10.1021/acs.analchem.5c04419

[203] X. Zhao, Y. Chen, R. Niu, Y. Tang, Y. Chen et al., NIR plasmonic nanozymes: synergistic enhancement mechanism and multi-modal anti-infection applications of MXene/MOFs. Adv. Mater. **36**(8), 2307839 (2024). 10.1002/adma.202307839 10.1002/adma.202307839 37812814

[204] F. Mo, S. Zhong, T. You, J. Lu, D. Sun, Aptamer and DNAzyme-functionalized Cu-MOF hybrid nanozymes for the monitoring and management of bacteria-infected wounds. ACS Appl. Mater. Interfaces **15**(45), 52114–52127 (2023). 10.1021/acsami.3c10682 37921634 10.1021/acsami.3c10682

[205] C. Liu, X. Zhao, Z. Wang, Y. Zhao, R. Li et al., Metal-organic framework-modulated Fe(3)O(4) composite au nanoparticles for antibacterial wound healing *via* synergistic peroxidase-like nanozymatic catalysis. J. Nanobiotechnol. **21**(1), 427 (2023). 10.1186/s12951-023-02186-6 10.1186/s12951-023-02186-6 PMC10647143 37968680

[206] H. Sun, J. Dan, Y. Liang, M. Li, J. Zhuo et al., Dimensionality reduction boosts the peroxidase-like activity of bimetallic MOFs for enhanced multidrug-resistant bacteria eradication. Nanoscale **14**(32), 11693–11702 (2022). 10.1039/d2nr02828j 35912946 10.1039/d2nr02828j

[207] T. Zhang, M. Tang, S. Yang, H. Fa, Y. Wang et al., Development of a novel ternary MOF nanozyme-based smartphone-integrated colorimetric and microfluidic paper-based analytical device for trace glyphosate detection. Food Chem. **464**, 141780 (2025). 10.1016/j.foodchem.2024.141780 39486279 10.1016/j.foodchem.2024.141780

[208] J. Xin, C. Shu, Y. Fu, X. Yu, Z. Wang et al., MOF-confined ultrafine nanozymes with enhanced catalysis for sensitive colorimetric detection of glucose. Talanta **283**, 127152 (2025). 10.1016/j.talanta.2024.127152 39500180 10.1016/j.talanta.2024.127152

[209] D. Ma, N. Li, D. Zhu, F. Li, Heterocyclic effect boosted peroxidase-like activity of MIL(Fe) metal-organic framework for colorimetric assay and dye removal. J. Hazard. Mater. **480**, 136148 (2024). 10.1016/j.jhazmat.2024.136148 39405683 10.1016/j.jhazmat.2024.136148

[210] X. Guan, X. Ge, H. Dong, J. Wei, J. Ouyang et al., Ultrathin 2D Pd/Cu single-atom MOF nanozyme to synergistically overcome chemoresistance for multienzyme catalytic cancer therapy. Adv. Healthc. Mater. **12**(30), 2301853 (2023). 10.1002/adhm.202301853 10.1002/adhm.202301853 37625419

[211] Y. Wang, Y. Pan, S. Sproules, J. Liu, R.S. Forgan, Multifunctional Fe-doped MOF-808 nanocomposites for chemo/chemodynamic synergistic therapy. Small **22**(4), e12728 (2026). 10.1002/smll.202512728 41311285 10.1002/smll.202512728 PMC12809198

[212] Y. Yang, D. Zhu, Y. Liu, B. Jiang, W. Jiang et al., Platinum-carbon-integrated nanozymes for enhanced tumor photodynamic and photothermal therapy. Nanoscale **12**(25), 13548–13557 (2020). 10.1039/d0nr02800b 32555859 10.1039/d0nr02800b

[213] Q. You, K. Zhang, J. Liu, C. Liu, H. Wang et al., Persistent regulation of tumor hypoxia microenvironment *via* a bioinspired Pt-based oxygen nanogenerator for multimodal imaging-guided synergistic phototherapy. Adv. Sci. **7**(17), 1903341 (2020). 10.1002/advs.201903341 10.1002/advs.201903341 PMC7507529 32995114

[214] M. Zhou, J. Feng, Q. Mei, T. Li, Y. Zhang et al., A powerful tumor catalytic therapy by an enzyme-nanozyme cascade catalysis (ENCAT) system. Small **21**(8), 2409363 (2025). 10.1002/smll.202409363 10.1002/smll.202409363 39828616

[215] P. Wang, H. Wang, D. Xu, J. Hu, B. Geng et al., A cascade ROS nanoamplifier for enhanced sono-chemodynamic therapy of osteosarcoma. Adv. Healthc. Mater., e04957 (2026). 10.1002/adhm.202504957 10.1002/adhm.202504957 41489351

[216] K. Ren, Y. Li, Q. Liu, Rapid on-site colorimetric detection of arsenic(V) by NH_2_-MIL-88(Fe) nanozymes-based ultraviolet-visible spectroscopic and smartphone-assisted sensing platforms. Anal. Chim. Acta **1336**, 343523 (2025). 10.1016/j.aca.2024.343523 39788676 10.1016/j.aca.2024.343523

[217] P. Arul, S.-T. Huang, C. Nandhini, C.-H. Huang, N.S.K. Gowthaman et al., Development of a nanozyme-based electrochemical catalyst for real-time biomarker sensing of superoxide and nitric oxide anions released from living cells and exogenous donors. Biosens. Bioelectron. **261**, 116485 (2024). 10.1016/j.bios.2024.116485 38852323 10.1016/j.bios.2024.116485

[218] H. Chen, X. Zhang, W. Gao, B. Huang, L. Han, Reversible uncompetitive inhibition of metal-organic framework nanozymes: specific colorimetric assay of methidathion without enzymes. Chem. Commun. **60**(92), 13522–13525 (2024). 10.1039/d4cc04417g 10.1039/d4cc04417g 39373975

[219] S.-T. Wu, Z.-Y. Qiu, H.-Q. Su, Y. Cao, S.-Q. Gao et al., Design of Mn-based nanozymes with multiple enzyme-like activities for identification/quantification of glyphosate and green transformation of organophosphorus. Biosens. Bioelectron. **263**, 116580 (2024). 10.1016/j.bios.2024.116580 39033653 10.1016/j.bios.2024.116580

[220] S.S. Mohammad Ameen, K.M. Omer, Multifunctional MOF: Cold/hot adapted sustainable oxidase-like MOF nanozyme with ratiometric and color tonality for nitrite ions detection. Food Chem. **462**, 141027 (2025). 10.1016/j.foodchem.2024.141027 10.1016/j.foodchem.2024.141027 39213963

[221] Z. Gao, J. Guan, M. Wang, S. Liu, K. Chen et al., A novel laccase-like Cu-MOF for colorimetric differentiation and detection of phenolic compounds. Talanta **272**, 125840 (2024). 10.1016/j.talanta.2024.125840 38430865 10.1016/j.talanta.2024.125840

